# Redox‐Switchable Complexes Based on Nanographene‐NHCs

**DOI:** 10.1002/chem.202201384

**Published:** 2022-06-16

**Authors:** César Ruiz‐Zambrana, Rajeev K. Dubey, Macarena Poyatos, Aurelio Mateo‐Alonso, Eduardo Peris

**Affiliations:** ^1^ Institute of Advanced Materials (INAM). Centro de Innovación en Química Avanzada (ORFEO-CINQA). Universitat Jaume I. Av. Vicente Sos Baynat s/n. Castellón. 12071 Spain; ^2^ POLYMAT University of the Basque Country UPV/EHU Avenida de Tolosa 72 20018 Donostia-San Sebastian Spain; ^3^ Ikerbasque, Basque Foundation for Science 48009 Bilbao Spain

**Keywords:** mechanism, nanographene, N-heterocyclic carbene, redox-switchable, cycloaddition

## Abstract

A series of rhodium and iridium complexes with a N‐heterocyclic carbene (NHC) ligand decorated with a perylene‐diimide‐pyrene moiety are described. Electrochemical studies reveal that the complexes can undergo two successive one‐electron reduction events, associated to the reduction of the PDI moiety attached to the NHC ligand. The reduction of the ligand produces a significant increase on its electron‐donating character, as observed from the infrared spectroelectrochemical studies. The rhodium complex was tested in the [3+2] cycloaddition of diphenylcyclopropenone and methylphenylacetylene, where it displayed a redox‐switchable behavior. The neutral complex showed moderate activity, which was suppressed when the catalyst was reduced.

## Graphical abstract

**A nanographene‐functionalized** NHC ligand was prepared and coordinated to rhodium an iridium. The presence of a perylene‐diimide moiety in the ligand allows that the resulting complexes show redox‐switching properties in the [3+2] cycloaddition of diphenylcyclopropenone and methylphenylacetylene.

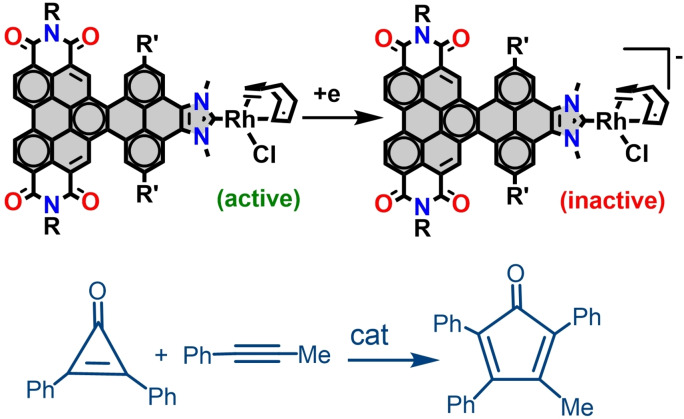

## Introduction

The synthesis of polycyclic aromatic hydrocarbons (PAHs) and nanographenes is attracting growing attention due to their potential as materials for organic electronics and photovoltaics.[^1^, ^2^, ^3^] The exchange of carbon atoms by heteroatoms (heteroatom‐doping) and the fusion of different heterocycles to the aromatic core has emerged as an efficient strategy for tailoring their optical and electronic properties, redox behavior, aromaticity and stability,[^1^, ^4^] hence the tunability of these nanographenes is of utmost importance to match properties with desired applications. The introduction of heteroatoms into the structure of the nanographene may allow enabling nanographene‐based ligands suitable for metal coordination, allowing the preparation of metal complexes with intriguing supramolecular properties.^[5]^ However, despite the recent advances in the synthesis of extended nanographenes,[^3^, ^6^] their use as ligands remains largely unexplored. The few metal complexes with nanographene‐containing ligands described so far have already shown great potential in supramolecular chemistry,[^5^, ^7^] and in homogeneous catalysis.^[8]^ In the field of N‐heterocyclic carbene (NHC) chemistry, some of us have incorporated extended polycyclic aromatic systems into the structure of NHC ligands for preparing families of NHC‐based metal complexes with enhanced catalytic properties,^[9]^ and supramolecular organometallic assemblies with interesting host‐guest chemistry properties.^[10]^ In some cases, we were able to construct nanosized NHC ligands connected to large nitrogen‐doped polycyclic aromatic hydrocarbons whose dimensions (polycyclic aromatic core >1 nm^[3]^) allowed their classification as nanographene‐functionalized NHCs.[^11^, ^12^] Still, further π‐extension of the backbone of the NHC remains challenging due to the lack of synthons suitable for the preparation of the imidazolium salts that are used as NHC‐precursors. With all the above it becomes obvious that finding synthetic approaches for introducing structural and functional modifications on both, nanographenes and NHC ligands, may create promising opportunities in the fields of nanoscience and molecular chemistry.

In two recent studies, we showed how the fusion of a naphthalene diimide (NDI) moiety on the backbone of an N‐heterocyclic carbene provided a ligand with redox‐switchable properties, which was used for the preparation of rhodium and iridium catalysts for the cycloisomerization of alkynoic acids,^[13]^ and in the preparation of gold(I) complexes for the hydroamination of alkynes.^[14]^ The functionalization of the NHC ligands with the NDI core, allowed three levels of electronic control of the catalyst, given that the ligand can operate either in its neutral form, or in the one‐ or two‐electron reduced forms of the NDI moiety, thus constituting a rare example of multi‐state redox switchable catalysis. We think that the scarcity of multi‐state switchable catalysts is rather surprising,^[15]^ given that multi‐state switchable molecules feature prominently in molecular machines^[16]^ and molecular electronics.^[17]^

**Scheme 1:**
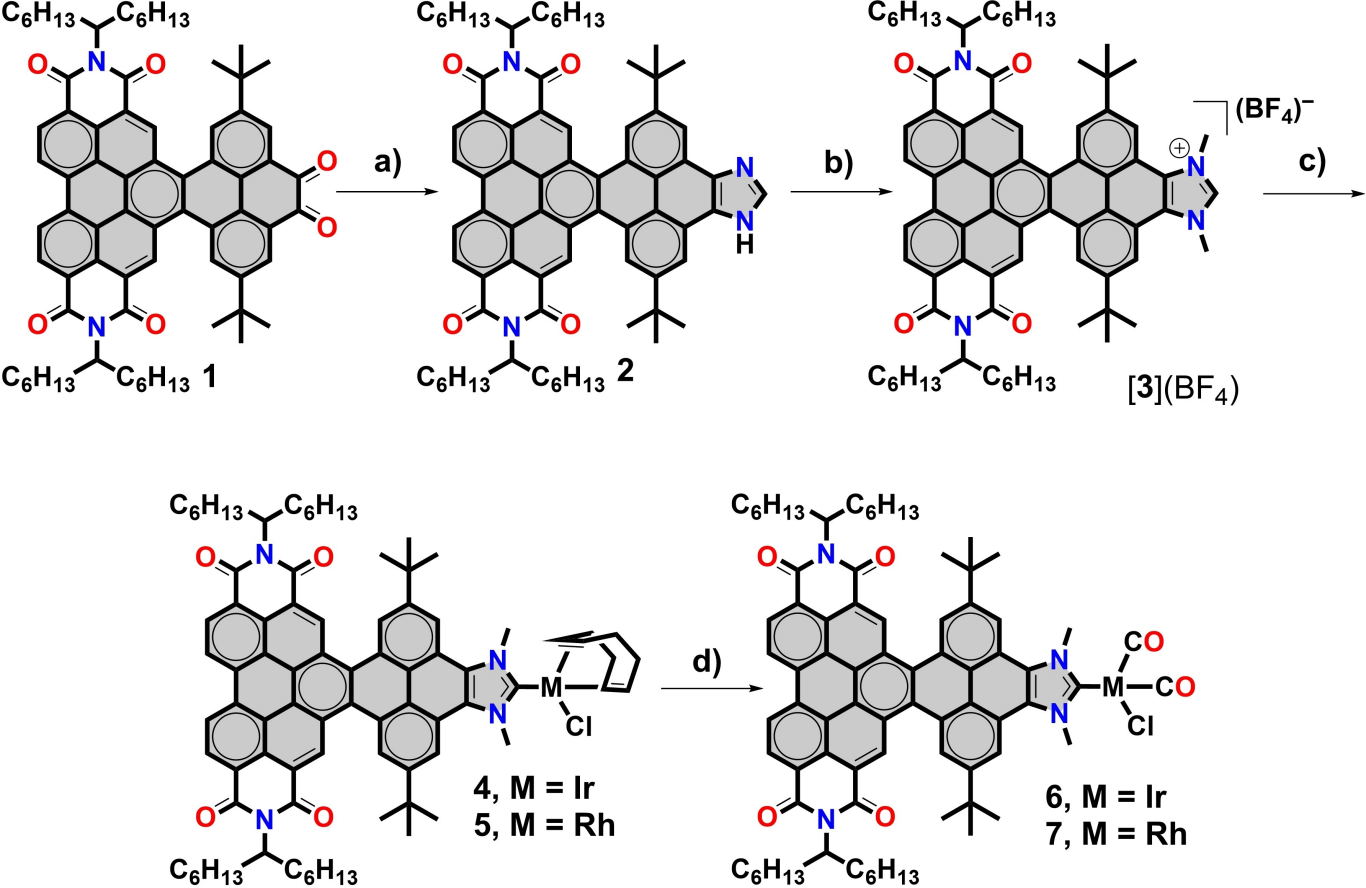
Preparation of nitrogen‐containing nanographene‐functionalized NHC complexes of rhodium and iridium. a) Formaldehyde and ammonium acetate, in acetic acid at 90 °C; b) NaHCO_3_ and CH_3_I in acetonitrile at 90 °C, followed by anion metathesis with [Et_3_O](BF_4_); c) tBuOK in dry THF followed by addition of [MCl(COD)]_2_, and d) CO bubbling in CH_2_Cl_2_ at 0 °C.

Herein, we report a series of perylene‐pyrene nanographene NHC iridium and rhodium complexes (**4**–**7**, Scheme 1) that exhibit multifunctional optical, electronic and catalytic properties associated with the presence of the extended perylene‐pyrene core. Given that the ligand contains a redox‐active PDI moiety, we also show that the electronic nature of the NHC ligand can be used to modify the catalytic activity of the metal complexes in the [3+2] cycloaddition of diphenyl‐cyclopropenone with methylphenylacetylene. As will be described below, our studies help to unveil important information regarding key mechanistic aspects of this catalytic reaction.

## Results and Discussion

The starting point for the synthesis of nanographene metal complexes **4**–**7** is PDI‐pyrene diketone **1**, which constitutes an excellent nm‐sized π‐scaffold for the construction of electron‐deficient nanographene N‐heterocyclic carbenes. Notably, this type of nanographenes are non‐planar because of the steric congestion generated by the inner hydrogen atoms at cove regions generated at the intersection between the pyrene and the perylene moieties.^[18]^ This lack of planarity endows the nanographenes with an optimal solubility for the preparation of metal carbenes. Dione **1** was obtained in six steps from PDI and pyrene chromophores (see details in the Supporting Information). The condensation of **1** with formaldehyde and ammonium acetate in acetic acid allowed the formation of the PDI‐pyrene‐imidazole **2** in quasi‐quantitative yield. The bisalkylation of **2** to form the corresponding N‐bis‐alkylated imidazolium salt was performed by treatment of **2** with NaHCO_3_ in acetonitrile followed by the addition of MeI. Further treatment of the resulting product with [Et_3_O](BF_4_) allowed anion metathesis to afford [**3**](BF_4_). The estimated distance between the C2‐H carbon of the imidazolium edge and one of the bay hydrogens at the perylene‐diimide moiety is of 1.5 nm, while the distance between the two nitrogen atoms of the PDI is of 1.1 nm. The imidazolium salt [**3**](BF_4_) showed to be an excellent NHC precursor, as it reacted with tBuOK in the presence of [MCl(COD)]_2_ (M=Rh or Ir) to form complexes **4** and **5** in 65 % and 45 % yield, respectively. All the synthetic steps to form complexes **4** and **5** can be viewed in Scheme 1. It is important to note that although many NHC metal complexes with fused polycyclic aromatic systems have been synthesized,[^9^, ^12^, ^19^, ^20^] to the best of our knowledge **4** and **5** are those with a larger number of aromatic rings and thus, with a larger extension of a π‐conjugated surface area attached to the backbone of the carbene.

All compounds **1**–**5** were characterized by ^1^H and ^13^C NMR spectroscopy. The ^1^H NMR spectra of **1**–**5** had in common the appearance of five resonances in the aromatic region, in agreement with the twofold symmetry of all molecules. The CDCl_3_^1^H NMR spectrum of the imidazolium salt [**3**](BF_4_) also displays a singlet at δ=9.74 assigned to the C2‐H proton of the imidazolium unit. The ^13^C NMR spectra of **4** and **5** show the diagnostic signals due to the carbene carbons at 188.1 and 192.2 ppm, respectively, the latter one being a doublet due to the Rh−C coupling (^1^ *J*_Rh‐C_=50 Hz).

In order to obtain information about the electron‐donating ability of this new PDI‐pyrene‐NHC ligand, the carbonyl complexes **6** and **7** were obtained by bubbling CO in a CH_2_Cl_2_ solution of **4** and **5** at 0 °C, as shown in Scheme 1. Both **6** and **7** were characterized by NMR and infrared (IR) spectroscopy. The IR spectrum (CH_2_Cl_2_) of **6** shows the signals due to the C−O stretching frequencies of the carbonyl ligands at 2071 and 1988 cm^−1^, while the rhodium complex **7** exhibited two bands at 2077 and 2006 cm^−1^. By using the well accepted correlation,^[21]^ and using the C−O stretching values of the iridium complex **6** as reference, the calculated Tolman Electronic Parameter (TEP) of this PDI‐pyrene‐NHC ligand is 2055 cm^−1^, therefore indicating that the ligand has similar electron‐donating abilities as our previously reported pyrene‐functionalized NHC ligands,[^19^, ^22^] but slightly less electron‐donating strength than our recently published NDI‐functionalized NHC ligand.^[13]^

**Figure 1:**
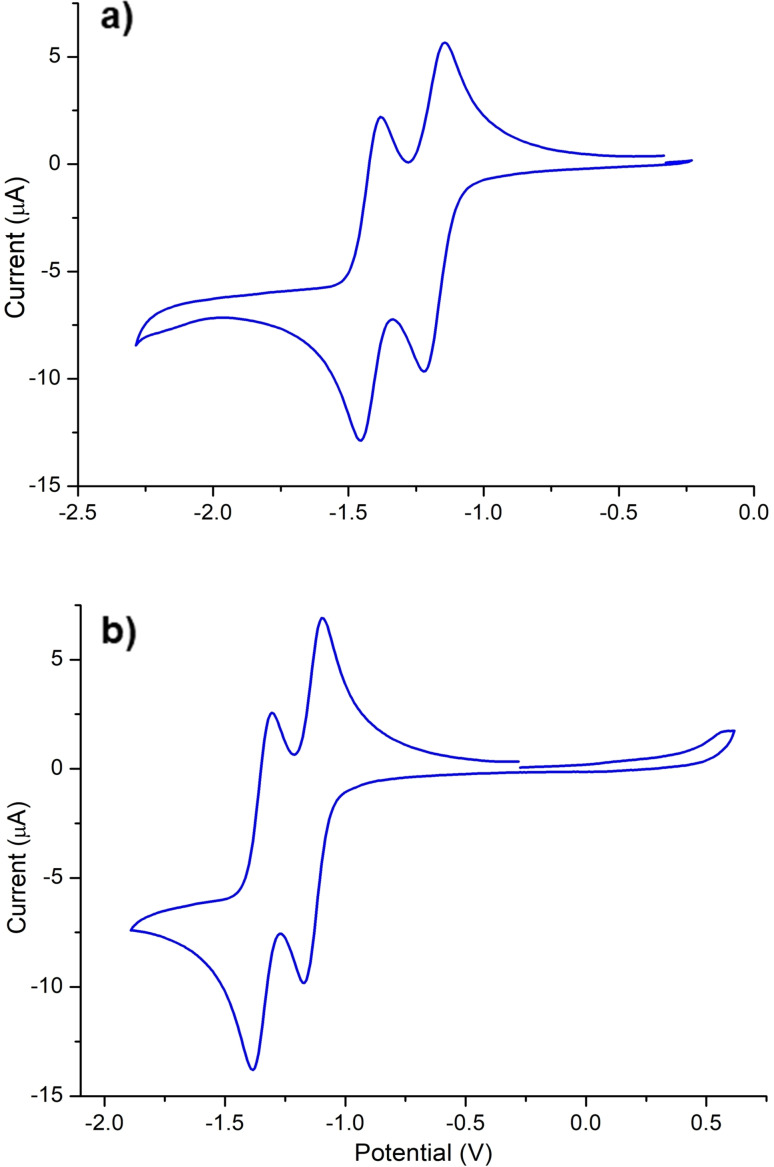
Cyclic Voltammograms of [**3**](BF_4_) (a) and **7** (b), in dry CH_2_Cl_2_ with 1 mM analyte and 0.1 M [N(*n*Bu)_4_][PF_6_]. Measurements performed at 100 mV s^−1^ and referenced vs. ferrocenium/ferrocene couple at 0 V.

Due to the presence of a redox‐active PDI moiety in this new NHC ligand, we considered important to perform cyclic voltammetry (CV) studies in order to explore the influence of the redox properties of the polyaromatic core upon coordination to the metal complex (Table 1). Figure 1 shows two representative cyclic voltammograms of the PDI‐pyrene‐imidazolium salt [**3**](BF_4_) and of the rhodium carbonyl complex **7**. The CV diagram for [**3**](BF_4_) reveals two separated reversible one‐electron reduction processes, which are consistent with the two reduction steps expected for a PDI moiety. The first process (E_1/2_=−1.18 V vs. Fc^+^/Fc) corresponds to the formation of a neutral radical species, while the second reduction (E_1/2_=−1.42 V vs. Fc^+^/Fc) is due to the formation of the doubly‐reduced negative species. These two redox values are significantly more negative than the values observed for the parent PDI core without the lateral substitution with a pyrene‐imidazolium fragment,^[23]^ probably because the presence of the electron‐rich pyrene moiety penalizes the reduction process. The CV diagram of the iridium and rhodium complexes **4** and **5** (see details in Supporting Information) show two reversible reduction waves at identical values, −1.21 and −1.42 V (vs. Fc^+^/Fc), and one irreversible oxidation wave at 0.54 V (for **4**) and 0.53 V (for **5**). The first reduction waves associated to one‐electron reduction of the PDI moiety are at a slightly more negative potential than the first reduction potential of the PDI‐functionalized imidazolium salt [**3**](BF_4_), thus indicating that the presence of the positive charge in [**3**](BF_4_) has small, but not negligible, influence on the reduction potential of the PDI moiety of the nanographene. The fact that **4** and **5** exhibit the same values for the two reduction potentials of the PDI core, indicates that the change of the metal has negligible influence on the electrochemical behavior of the PDI‐pyrene‐NHC ligand. Interestingly, the reduction potentials associated to the reduction of the PDI core of the carbonylated complexes **6** and **7** are significantly less negative (80–70 mV) than those shown by the related iridium and rhodium complexes with COD (**4** and **5**), strongly suggesting the coupling between the metal center and the PDI core of the molecule.

**Table 1:** Electrochemical properties of compounds [**3**](BF_4_)‐**7**.^[a]^

Compound	*E*_1/2_ [V]/ΔE [mV]	*E*’_1/2_ [V]/ΔE [mV]	*E*_p_^[a]^ [V]
[**3**](BF_4_)	−1.42/79	−1.18/79	–
**4**	−1.42/78	−1.21/102	0.54
**5**	−1.42/69	−1.21/72	0.53
**6**	−1.33/61	−1.14/106	–
**7**	−1.34/79	−1.13/71

[a] Cyclic voltammograms performed in dry CH_2_Cl_2_ with 1 mM analyte and 0.1 M [N(*n*Bu)_4_][PF_6_]. Measurements performed at 100 mV s^−1^ and referenced vs. ferrocenium/ferrocene.

**Figure 2:**
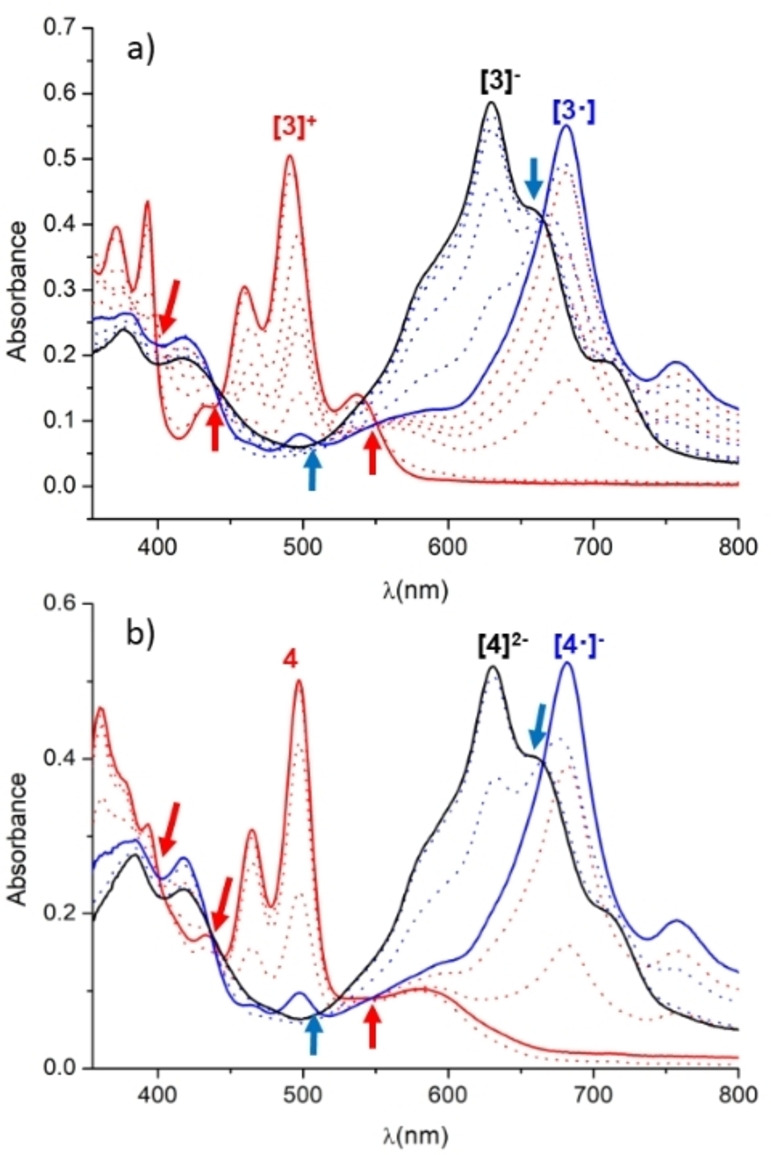
UV‐vis SEC monitoring reduction of a) [**3**](BF_4_) and b) **4**, in dry CH_2_Cl_2_ (0.1 M [N(*n*Bu)_4_][PF_6_]. The electrochemical reduction was performed applying progressively lower potentials with a Au working electrode, Pt counter‐electrode, and Ag wire pseudo‐reference electrode. The solid lines represent the spectra of the starting (red), singly‐reduced (blue) and doubly‐reduced (black) species. Isosbestic points are marked with arrows (red for first reduction, blue for second reduction).

In order to gain information about the nature and stability of the species formed upon one‐ and two‐electron reduction of the PDI‐pyrene‐imidazolium salt [**3**](BF_4_), and the PDI‐pyrene‐NHC‐MCl(COD) complexes **4** and **5**, we performed a series of spectroelectrochemical (SEC) experiments using an Optically Thin Transparent Layer Electrochemical (OTTLE) cell. The experiments were performed in CH_2_Cl_2_ by progressively applying more negative potentials while recording the UV‐Vis spectra of the species generated in the solution. As can be observed in Figure 2a, the UV‐vis spectrum of [**3**](BF_4_) shows two strong vibronically resolved bands with maxima at 372 and 490 nm, which are assigned to the absorption of the pyrene and PDI cores of the molecule, respectively. Upon application of increasing electrochemical potentials, the two bands assigned to [**3**]^**+**^ decrease, and two new bands at 378 and 681 nm, associated to the one‐electron reduced species [**3**⋅] appeared. The band at 691 nm is typically observed for PDI radical anions,[^24^, ^25^] thus strongly suggests that the reduction is produced at the PDI core. Further electrochemical reduction produces the appearance of a new band at 629 nm, which is indicative of the two‐electron reduction of the PDI core, and thus is attributed to the diamagnetic species [**3**]^−^ (PDI‐based dianions are diamagnetic,^[25]^ as is typical for most aromatic dianions^[26]^). An interesting observation that needs to be mentioned here, is that the analysis of the series of spectra obtained along this SEC experiment reveals that the transition between [**3**]^**+**^→[**3**⋅] and [**3**⋅]→[**3**]^−^ steps of the process display clear isosbestic points indicating that both transitions are produced without the appearance of further reaction intermediates, and that all three species detected in the experiment are stable under the conditions used to carry out the measurements. Similarly, SEC experiments were also carried with the rhodium and iridium complexes **4** and **5**. Figure 2b shows the series of absorption spectra obtained for the SEC experiments carried out using the iridium complex **4**. The UV‐vis spectrum of **4** shows two intense vibronically coupled bands with maxima at 360 and 497 nm, which are associated to the absorption of the pyrene and PDI moieties of the molecule, respectively. The application of the electrochemical potential progressively produces the appearance of two new set of bands with maxima at 417 and 682 nm, which are assigned to the one‐electron reduced complex [**4**⋅]^−^. Further reduction results in the disappearance of these two bands and the appearance of two new ones with maxima at 417 and 631 nm, which are assigned to the doubly reduced species [**4**]^2−^. As in the case of the experiments performed with [**3**](BF_4_), the UV‐vis bands emerging from the reduction of **4** indicate that two reductions are produced at the PDI core. Again, the transition between the three species (**4**, [**4**⋅]^−^ and [**4**]^2−^) occurred without detection of intermediates, as indicated by the presence of clear isosbestic points for each of the two steps. This observation also suggests that the three species involved in the reduction process are stable under the conditions used for the experiments. In order to explore the reversibility of the process, we carried out the electrochemical reduction to produce directly [**4**]^2−^, and then we applied positive potentials to progressively oxidize [**4**]^2−^ to [**4**⋅]^−^, and then [**4**⋅]^−^ to **4**. The experiment showed that the stepwise oxidation of [**4**]^2−^ produced the bands associated to [**4**⋅]^−^, and then to **4**, with the same exact intensities as the ones from the original solution of **4** used prior to its bulk two‐electron reduction. This experiment demonstrates not only the reversibility of the process, but also that all three species involved in the electrochemical experiment are stable under the conditions used to carry out the experiment. Due to their relevance in the catalytic experiments that will be discussed below, analogous SEC experiments were carried out for the rhodium complexes **5** and **7**, for which similar conclusions regarding the reversibility of the reduction processes could be extracted (see Figure S36 and S37 for more details).

**Figure 3:**
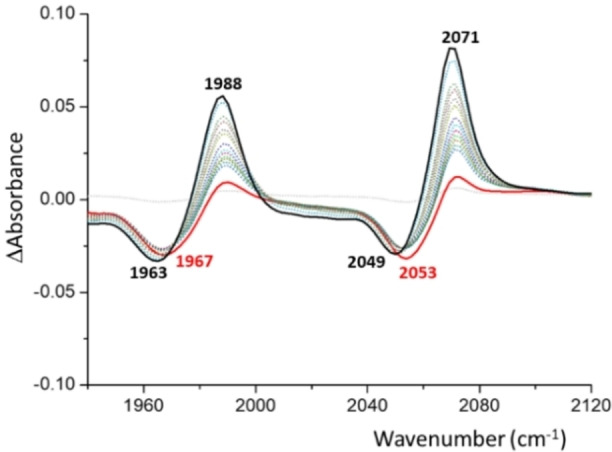
Infrared difference spectra resulting from the IR‐SEC reduction of **6** in dry CH_2_Cl_2_ (0.1 M [N(*n*Bu)_4_][PF_6_]). The electrochemical reduction was performed applying progressively lower potentials with an Au working electrode, Pt counter electrode, and Ag wire pseudo‐reference electrode.

IR‐SEC experiments on **6** were used to assess the changes in the electron‐donating character of the PDI‐pyrene‐NHC ligand upon reduction (Figure 3). In principle, we would expect that the reduction of the ligand should increase the electron‐donor ability of the ligand, and this should have an effect on the C−O stretching frequencies observed in the resulting IR spectrum of the product. The reduction of **6** is accompanied by a decrease of the intensity of the C−O stretching bands at 2071 and 1988 cm^−1^, and the appearance of two new ones at 2053 and 1967 cm^−1^, therefore producing and average Δ(CO) shift of −19,5 cm^−1^. This change may be assigned to the one‐electron reduction of **6** to form [**6**]^−^. As more negative potentials are applied, a new set of bands at 2049 and 1963 cm^−1^ progressively appear, which are assigned to the doubly reduced compound [**6**]^2−^. This two‐electron reduction produces an average Δ(CO) shift of −23,5 cm^−1^, therefore the TEP value is reduced from 2055 cm^−1^ for the neutral complex **6**, to 2035 cm^−1^ for [**6**]^2−^. To our knowledge, this is the lowest TEP value observed for a C2‐coordinated imidazole‐based NHC ligand.^[27]^ In fact, this TEP value reflects that this 2e‐reduced NHC has superior donating properties than the more strong‐donating mesoionic carbenes,^[28]^ and even than most cyclic (alkyl)(amino)carbenes (CAACS).^[29]^

**Scheme 2:**
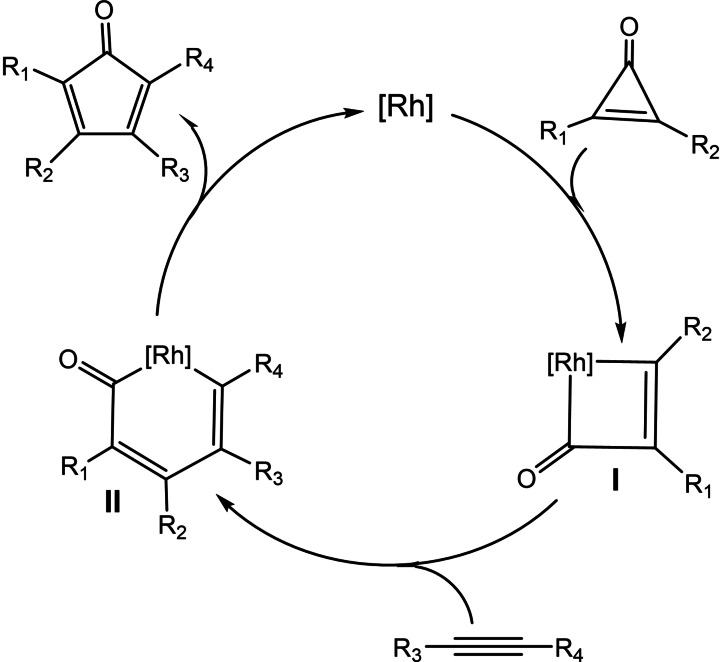
Catalytic cycle for the [3+2] cycloaddition of diphenylcyclopropenone and alkynes.

Considering the redox‐reversible character of the PDI‐pyrene‐NHC ligand, and once confirmed the modification of the electronic nature of the metal centers in the rhodium and iridium complexes upon reduction of the ligand, we decided to explore the redox‐switchable character of the ligand in one model catalytic reaction. Given that the reduction of the ligand has an important impact on its electron‐donating character, we chose to study the catalytic process for which the rate determining step (RDS) is either an oxidative addition or a reductive elimination. In principle, it is expected that strong electron‐donating ligands should facilitate catalytic processes whose RDS is an oxidative addition, while should show reduced activity in those catalytic processes for which the RDS involves a reductive elimination.^[30]^ The rhodium catalyzed [3+2] cycloaddition of diphenylcyclopropenone and alkynes to form cyclopentadienones was first reported by Wender and co‐workers in 2006.^[31]^ Since then, just a handful of rhodium complexes have been used to facilitate this process.[^29^, ^32^, ^33^] Cyclopentadienones constitute an important class of molecules with demonstrated value in many fields, such as ligands for transition metal catalysts,^[34]^ as components for de design of materials with photo‐ and electro‐luminescent properties,^[35]^ and as biosynthetic intermediates.^[36]^ In addition, due the their strong absorbing chromophoric properties, cyclopentadienones are utilized in the fabrication of LEDs and photovoltaics.^[37]^ The accepted cycle for the rhodium‐catalyzed reaction starts with the oxidative addition of the aryl‐carbon bond of the cyclopropenone to the rhodium center to form a rhodacyclobutenone (**I**), followed by the insertion of the alkyne forming a six‐membered rhodacycle (**II**), and final reductive elimination delivering the cyclopentadienone product (Scheme 2).^[38]^ We chose this reaction as a proof of concept for testing the activity of our complexes, because the catalytic cycle includes both an oxidative addition and a reductive elimination step. To the best of our knowledge, there are not clear reports indicating which of these two steps is the RDS of the process, an information that is crucial for designing effective catalysts for the process. Having a catalyst for which we can selectively switch the electron‐donating character of the ligand without altering its steric properties may provide a good opportunity to shed light on the process by performing relatively simple experiments.

For the study of the [3+2] cycloaddition of diphenylcyclopropenone and alkynes we used complexes **4**–**7** as prospective catalysts. The reactions were carried out by mixing diphenylcyclopropenone (0.27 mmol) and methylphenylacetylene (0.18 mmol) in deuterated toluene at 110 °C. We observed that the iridium complexes **4** and **6** were completely inactive in the reaction, regardless the amount of catalyst used, while the rhodium complexes **5** and **7** were moderately active. As can be observed in the data shown in Table 2, using a catalyst loading of 1 mol %, the COD‐containing complex **5** is able to produce 23 % of cyclopentadienone (**8**) after 6 h of reaction. Under the same reaction conditions, the carbonylated complex **7** is able to yield 24 % of the final product. If the reactions were allowed to react for longer times, we observed that the maximum amount of product formed was 29 % (for **5**), and 32 % (for **7**). This maximum amount of product was obtained after 9 h of reaction, and longer reaction times did not enhance the yield. When the reactions were performed using catalyst loadings of 2.5 and 5 mol % of **5**, we obtained maximum yields of 31 and 46 %, respectively, after 9 h of reaction.

**Table 2:** Activity of complexes **4**–**7** in the [3+2] cycloaddition of diphenylcyclopropenone with methylphenylacetylene.^[a]^

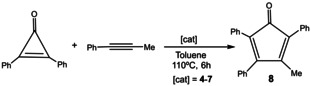				
Entry	Catalyst	Cat. load. [mol %]	Yield [%] (6 h)	Yield [%] (9 h)
1	**4**	1	0	0
2	**5**	1	23	29
3	**6**	1	0	0
4	**7**	1	24	32
5	**5**+[CoCp_2_]	1	0	0
6	**7**+[CoCp_2_]	1	0	0
7	**5**	2,5	25	30
8	**5**	5	39	46
9	**5**+ [CoCp_2_]	5	0	0

[a] Reactions carried out in toluene‐d_8_ at 110 °C, using 0.18 mmol of methylphenylacetylene and 0.27 mmol of diphenylcyclopropenone. Yields determined by ^1^H NMR using 1,3,5‐trimethoxybenzene as integration standard. The reactions carried out in the presence of cobaltocene were performed adding 1.5 equivalents of cobaltocene with respect to catalyst.

We next reproduced the reactions using **5** and **7** as catalysts but adding one equivalent of cobaltocene. With a redox potential of −1.33 V (vs. Fc^+^/Fc),^[39]^ cobaltocene is a suitable reagent for the one‐electron reduction of **5** and **7**. As can be seen from the data shown in Table 2, catalysts **5** and **7** were completely inactive in the presence of cobaltocene. This simple experiment suggests that the RDS of the catalytic process is the reductive elimination from the rhodacycle **II** to release the cyclopentadienone, since increasing the electron‐donating character of the ligand upon its reduction is expected to reduce the ability of the catalyst to undergo the reductive elimination step.

The observation of the suppression of the catalytic activity of the reduced catalyst could also be ascribed to the decomposition of the rhodium complex upon addition of cobaltocene. This possibility could not be discarded even though the CV and the spectroelectrochemical studies showed that both [**5**⋅]^−^ and [**7**⋅]^−^ are rather stable species at room temperature. In order to discard this possibility, we decided to perform more detailed catalytic studies. For these studies we took the cyclooctadiene rhodium complex **5** as model catalyst. By calculating the reaction rates at different concentrations of **5**, we determined that the reaction is half‐order with respect to the catalyst. Half‐order behavior is an indication of either a rapid equilibrium between species of different nuclearity (e. g., monomer‐dimer),^[40]^ or the rapid and reversible equilibrium with an off‐cycle non‐active species.^[41]^

**Figure 4:**
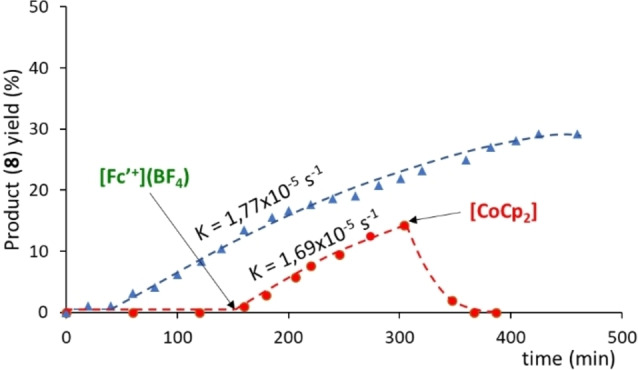
Time‐dependent reaction profile of the reaction of [3+2] cycloaddition of diphenylcyclopropenone and methylphenylacetylene using 2.5 mol % of **5**. The reactions were performed in toluene‐d^8^ at 110 °C. The reaction was monitored by ^1^H NMR using 1,3,5‐trimethoxybenzene as integration standard. The red plot (red circles) was carried out starting with **5**+cobaltocene and further addition of [Fe(η^5^‐C_5_H_4_COCH_3_)Cp](BF_4_) ([Fc’^+^](BF_4_)) at the indicated point. The blue plot (blue triangles), is the control experiment carried out using catalyst **5** without any additives.

**Scheme 3:**
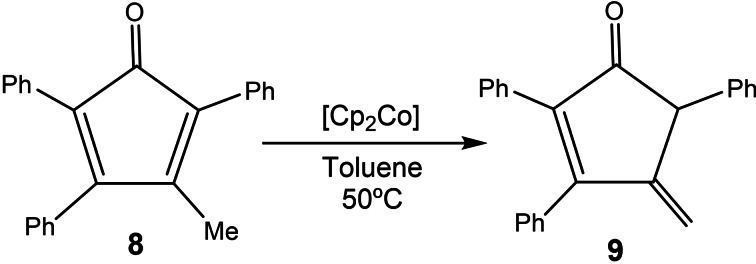
Isomerization of 3‐methyl‐2,4,5‐triphenylcyclopenta‐2,4‐dien‐1‐one (**8**) to cyclopentenone, 4‐methylene‐2,3,5‐triphenylcyclopent‐2‐en‐1‐one (**9**) catalyzed by [CoCp_2_] at room temperature.

Next, we performed an experiment aiming to determine if the inactive one‐electron‐reduced catalyst could be reactivated after being re‐oxidized with a suitable oxidant. As can be seen in the time‐dependent reaction profile shown in Figure 4, the reaction carried out with **5** in the presence of cobaltocene (which generates [**5**⋅]^−^) does not produce any product during the first two hours of reaction. Then, acetylferrocenium tetrafluoroborate was added in order to re‐oxidize the catalyst back to its active form, **5**, and we allowed the reaction to evolve for another 2.5 h. During this period of time, the catalyst recovered its activity and produced 15 % of product. We also performed a control experiment in which we followed the reaction without addition of any redox additives (see blue plot in Figure 4). In the resulting reaction profile, we observed that the reaction initiated after an induction time of one hour. A quick analysis of the two plots displayed in Figure 4 allows to observe that the profile of the control reaction is perfectly parallel to the profile in which the catalyst was re‐activated. More interesting is the fact that the activity of the catalyst is fully restored after the addition of [Fe(η^5^‐C_5_H_4_COCH_3_)Cp](BF_4_), as can be observed by comparing the corresponding rate constants (see Figure 4). This experiment strongly suggests that the addition of cobaltocene does not decompose the catalyst, but rather transforms it into a dormant form. This observation constitutes an additional support that the RDS of the cycloaddition reaction is the reductive elimination step. We wanted to know if we could further suppress the activity of the catalyst after the addition of acetylferrocenium tetrafluoroborate, and for that reason we then added cobaltocene and let the reaction evolve for another 2.5 h. As can be observed from the red plot shown in Figure 4, addition of cobaltocene made that the product yield dropped down to 0 % after one hour of reaction. This is explained because concomitant with the disappearance of the product, we observed the appearance of a new product, **9**, which results from the isomerization of **8**,^[33]^ as depicted in Scheme 3. In an independent experiment, we reacted **8** in the presence of 1 mol % of cobaltocene and observed that the isomerization of **8** was quantitative at 50 °C in just five minutes, thus indicating that the rhodium complex does not participate in this reaction.

**Figure 5:**
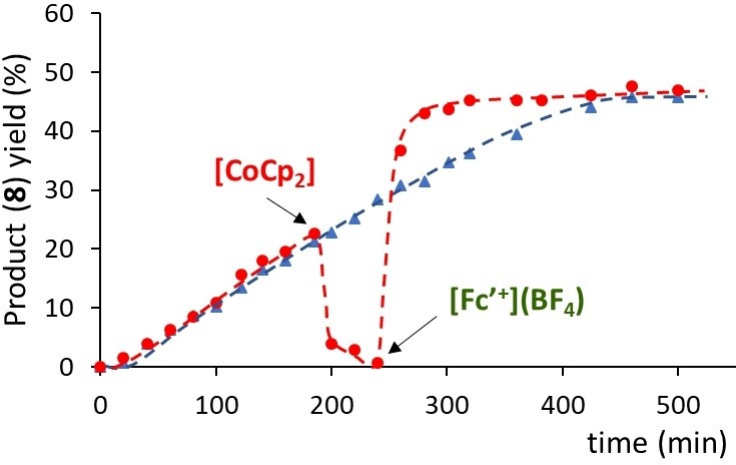
Time‐dependent reaction profile of the reaction of [3+2] cycloaddition of diphenylcyclopropenone and methylphenylacetylene using 5 mol % of **5**. The reactions were performed in toluene at 110 °C. The red plot (red circles) was carried out by adding cobaltocene and [Fe(η^5^‐C_5_H_4_COCH_3_)Cp](BF_4_) ([Fc’^+^](BF_4_)) at the indicated points. The blue plot (blue triangles), is the control experiment carried out using catalyst **5** without any additives.

We also performed an experiment in which the reaction between diphenylcyclopropenone and methylphenylacetylene was allowed to proceed for three hours in the presence of 5 mol % of **5** (Figure 5). This produced **8** in 23 % yield. Then, we added one equivalent of cobaltocene and observed the rapid disappearance of **8**, with the concomitant formation of the isomerization product, **9**. When all compound **8** was converted into **9**, we added acetyl ferrocenium tetrafluoroborate and observed that the activity of the catalyst was drastically recovered, and the reaction evolved until a maximum production of **8** of 50 % yield. At this point the conversion was complete (both starting reagents were consumed), although we did not detect the formation of any further products than **8** (50 %) and **9** (23 %). An issue that remains unclear for us is why the reactivation of the catalyst with acetyl ferrocenium tetrafluoroborate makes that the reaction becomes significantly faster than the reaction carried out with the catalyst in the absence of any external additive. A plausible explanation is that the presence of acetyl ferrocenium inhibits the formation of the inactive off‐cycle species, but this is an issue that we will study in more detail in our future research.

## Conclusion

In summary, by starting from a pyrene‐diketone fused with a PDI core, we obtained a N‐doped‐nanographene‐functionalized imidazolium salt that was suitable for the preparation of a series of nanographene‐functionalized‐NHC complexes of rhodium and iridium. This new NHC ligand can be regarded as the largest nanographene‐functionalized mono‐NHC ligand ever reported. It is worth mentioning that the PDI‐pyrene scaffold is expected to be non‐planar due to the steric congestion generated by the inner hydrogen atoms at the two cove regions,^[18]^ thus providing an interesting flexible structure from which helical conformations may arise. The preparation of this ligand and its coordination to rhodium and iridium does not only constitute a synthetic challenge for accessing to aesthetically beautiful fan‐shaped structures, but also enlarges the toolbox of synthetic methods for the bottom‐up approach of metal‐containing molecules for which size and dimensionality are of particular interest.

The reduction of the PDI moiety of the ligand produces a substantial increase of the electron‐donating abilities of the NHC, as demonstrated by the IR spectroelectrochemical studies of a carbonyl complex bearing the nanographene‐NHC ligand. In fact, the doubly reduced ligand constitutes one of the most electron‐donating NHCs that has been described to date. As a proof of concept, the rhodium complexes were tested in the [3+2] cycloaddition of diphenylcyclopropenone and methylphenylacetylene to form cyclopentadienone, where it was shown that the activity of both the COD‐ and CO‐containing complexes featured moderate activity. We also observed that the activity of these two catalysts was suppressed in the presence of cobaltocene. This result sheds light into the mechanism of the catalytic process, because the one‐electron reduction of the catalyst suppresses the ability of the complex to undergo the final reductive elimination of the cyclopentadienone product, therefore suggesting that this is the RDS of the cycle. These results underscore the utility of redox‐switchable catalysts to unveil key information about the mechanisms of catalytic reactions.

Also of importance is the fact that the PDI‐pyrene diketone **1**, which we used as starting material for the preparation of our nanographene‐NHC‐based metal complexes, constitutes itself a molecule with large potential catalytic applications, as 1,2‐diketone functionalities at the edge of graphenes are known to establish catalytic activity in the oxidative dehydrogenation of alkanes to alkenes,^[42]^ a process for which, to the best of our knowledge, catalysts with redox‐tunable properties have not been described.

Finally, we think that this nanographene‐functionalized ligand may have important applications in other research fields, such as supramolecular chemistry, where it is easy to imagine that this type of ligands may provide intriguing new structures, such as nanosized metallotweezers or even new examples of the novel type of mechanically interlocked molecules, the clippanes.^[43]^

## Conflict of interest

The authors declare no conflict of interest.

1

## Supporting information

As a service to our authors and readers, this journal provides supporting information supplied by the authors. Such materials are peer reviewed and may be re‐organized for online delivery, but are not copy‐edited or typeset. Technical support issues arising from supporting information (other than missing files) should be addressed to the authors.

Supporting Information

## Data Availability

The data that support the findings of this study are available from the corresponding author upon reasonable request.
