# Supplementary material for: Redox‐Switchable Complexes Based on Nanographene‐NHCs
Source: Chemistry. 2022 Jun 16;28(44):e202201384. doi: 10.1002/chem.202201384 (PMC9400984; doi:10.1002/chem.202201384)
Supplement: Supplementary file 1 — Supporting Information [file CHEM-28-0-s001.pdf]

Chemistry–A European Journal

Supporting Information

Redox-Switchable Complexes Based on Nanographene-NHCs

César Ruiz-Zambrana, Rajeev K. Dubey, Macarena Poyatos,* Aurelio Mateo-Alonso,* and Eduardo Peris*

**General considerations**	**S1**
**1. Synthesis of PBI-pyrene-diketone 1**	**S2-S5**
**2. Synthesis and characterization of the ligand precursor**	**S6-S7**
**3. Synthesis and characterization of the complexes**	**S8-S10**
**4. Spectroscopic data**	**S11-S21**
4.2. ^{1}H and ^{13}C spectra of **D** in $CDCl_3$	S11
4.3. ^{1}H and ^{13}C spectra of **E** in $CDCl_3$	S12
4.4. ^{1}H and ^{13}C spectra of **1** in $CDCl_3$	S13
4.5. ^{1}H and ^{13}C spectra of **2** in $CDCl_3$	S14
4.6. ^{1}H and ^{13}C spectra of **3** in $CDCl_3$	S15
4.7. ^{1}H, ^{13}C, HSQC and HMBC spectra of **4** in $CDCl_3$	S16
4.8. ^{1}H and ^{13}C spectra of **5** in $CDCl_3$	S18
4.9. ^{1}H, ^{13}C and HMBC spectra of **6** in $CDCl_3$ High-resolution mass spectra	S19
4.10. ^{1}H, ^{13}C and HMBC spectra of **7** in $CDCl_3$	S20
**5. HR MALDI-TOF and HR ESI-Q-TOF mass spectra**	**S22-S23**
**6. Electrochemical studies**	**S24-S28**
6.1. Electrochemical measurements	S24
6.2. Spectroelectrochemical measurements	S25
**7. Photophysical analysis**	**S29-S32**
**8. Catalytic studies**	**S33-S38**
8.1. Cycloaddition of diphenylcyclopropenone with methylphenylacetylene	S33
8.2. Redox switching experiments	S35
8.3. Isomerization of cyclopentadienone	S37
**9. References**	S39

General considerations. Anhydrous solvents were dried using a solvent purification system (SPS M BRAUN) or purchased and degassed prior to use by purging them with dry nitrogen. N,N'-Bis(1-hexylheptyl)-perylene bisimide[1] (**A**) and N,N´-bis(1-hexylheptyl)-1-bromo-perylene-3,4,9,10-tetracarboxy bisimide[2] (**B**) were prepared according to the literature. All the other reagents were used as received form the commercial suppliers. The NMR spectra were recorded on a Bruker 400 or 300 MHz, using $CDCl_3$ as solvent. The chemical shift values are given in ppm and *J* values in Hz. Signals marked with an asterisk (*) in the NMR spectra correspond to Apiezon brand H grease.[3] Electrospray mass spectra (ESI-MS) were recorded on a Waters TQD Triple Quadrupole Mass Spectrometer. High-resolution mass spectra of [**3**](BF_4) and **5** were performed using a Waters SYNAPT XS High-Definition Mass Spectrometer equipped with an electrospray ionization (ESI) source. High-resolution mass spectra of compounds **D**, **E** and **1** were recorded by Dr. Javier Calvo on an UltrafleXtreme III MALDI tandem mass spectrometer (Bruker) in reflector acquisition operation mode and the samples were prepared in THF. The matrix and salts used are indicated below each spectrum. UV-Visible absorption spectra were recorded on a Varian Cary 300 BIO spectrophotometer using dry and degassed dichloromethane under ambient conditions. Emission spectra were recorded on a modular Horiba FluoroLog-3 spectrofluorometer employing dry and degassed dichloromethane. Quantum yields were measured using a Hamamatsu integrating sphere at excitation wavelength of 470 nm.

1. Synthesis and characterization of PBI-pyrene-diketone 1

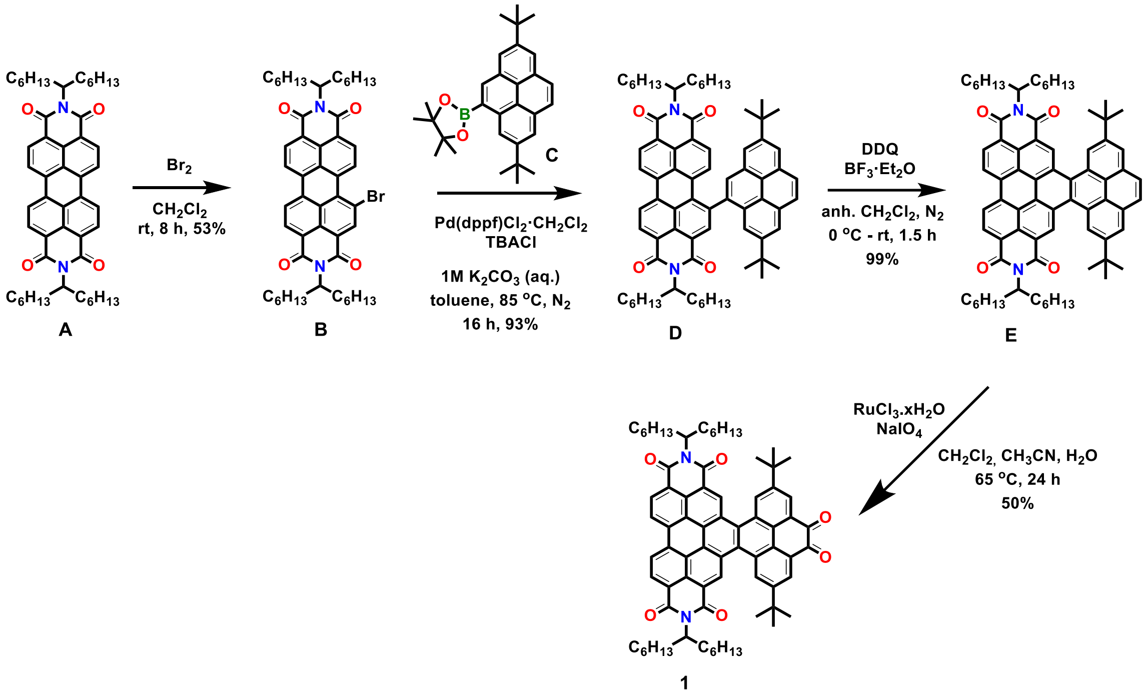

Scheme S1. Synthesis of PBI-pyrene-diketone **1**

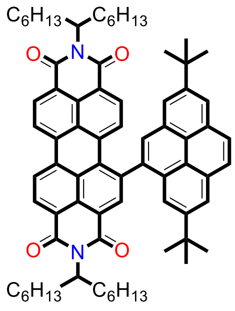

Compound D. A 500 mL round-bottomed flask was charged with 1-bromo-PBI **B**[2] (2.00 g, 2.40 mmol, 1 eq.), pyrene derivative **C**[4] (1.16 g, 2.64 mmol, 1.1 eq.), tetrabutylammonium chloride (0.067 g, 0.24 mmol, 0.1 eq.), and Pd(dppf)Cl_2·CH_2Cl_2 (0.20 g, 0.24 mmol, 0.1 eq.). Subsequently, toluene (150 mL) and 1M aqueous solution of K_2CO_3 (100 mL) was added. The reaction mixture was deaerated using vacuum and backfilled with nitrogen three times. Thereafter, the flask was sealed using a rubber septum and a N_2 balloon was placed. The reaction temperature was raised to 85 ^{o}C and the reaction was stirred for 16 h under an inert atmosphere. After being cooled to room temperature, the organic phase was separated using a separatory funnel and washed with water (2 x 200 mL). The organic phase was collected, and toluene was evaporated under reduced pressure. The solid residue was chromatographed on silica, eluting with 2:1 hexane-CH_2Cl_2. The product was isolated as dark-red crystalline solid in 93% yield (2.39 g). The ^{13}C NMR spectra shows the presence of two isomers in solution.**^{1}H NMR** (400 MHz, $CDCl_3$): δ 8.92–8.73 (m, 3H, CH_{Ar}), 8.73–8.63 (m, 2H, CH_{Ar}), 8.37–8.30 (m, 2H, CH_{Ar}), 8.27 (s, 1H, CH_{Ar}), 8.17–8.06 (m, 3H, CH_{Ar}), 8.06-8.01 (m, 1H, CH_{Ar}), 7.82 (br s, 1H, CH_{Ar}), 7.65–7.54 (m, 1H, CH_{Ar}), 5.28–4.92 (m, 2H, C$H(C_6H_{13})_2$), 2.37–2.22 (m, 2H, CH(C$H_2CH_2CH_2CH_2CH_2CH_3)_2$), 2.22–1.99 (m, 2H,

$CH(C\mathit{H}_2CH_2CH_2CH_2CH_2CH_3)_2)$, 1.95–1.80 (m, 2H, $CH(C\mathit{H}_2CH_2CH_2CH_2CH_2CH_3)_2)$, 1.80–1.66 (m, 2H, $CH(C\mathit{H}_2CH_2CH_2CH_2CH_2CH_3)_2)$, 1.62 (s, 9H, $C(C\mathit{H}_3)_3)$,1.57 (s, 9H, $C(C\mathit{H}_3)_3)$, 1.44–1.06 (m, 32H, $CH(CH_2C\mathit{H}_2C\mathit{H}_2C\mathit{H}_2C\mathit{H}_2CH_3)_2)$, 0.92– 0.71 (m, 12H, $CH(CH_2CH_2CH_2CH_2CH_2C\mathit{H}_3)_2))$.. **$^{13}C\{^1H\}$ NMR** (126 MHz, $CDCl_3$): δ 165.1 (*C*=O), 165.0 (*C*=O), 164.8 (*C*=O), 164.6 (*C*=O) 164.0 (*C*=O), 163.9 (*C*=O), 163.8 (*C*=O), 163.4 (*C*=O), 149.5 (C_{Ar}), 148.9 (C_{Ar}), 140.0 (C_{Ar}), 139.9 (C_{Ar}), 138.3 (C_{Ar}), 137.6 (C_{Ar}), 135.2 (C_{Ar}), 134.9 (C_{Ar}), 134.9 (C_{Ar}), 134.5 (C_{Ar}), 134.0 (C_{Ar}), 134.0 (C_{Ar}), 131.6 (C_{Ar}), 131.2 (C_{Ar}), 130.9 (C_{Ar}), 130.5 (C_{Ar}), 129.3 (C_{Ar}), 129.2 (C_{Ar}), 129.1 (C_{Ar}), 129.0 (C_{Ar}), 128.2 (C_{Ar}), 128.0 (C_{Ar}), 127.8 (C_{Ar}), 127.2 (C_{Ar}), 126.8 (C_{Ar}), 126.8 (C_{Ar}), 124.1 (C_{Ar}), 124.1 (C_{Ar}), 123.8 (C_{Ar}), 123.8 (C_{Ar}), 123.4 (C_{Ar}), 123.3 (C_{Ar}), 123.1 (C_{Ar}), 123.0 (C_{Ar}), 122.2 (C_{Ar}), 120.1 (C_{Ar}), 54.9 ($\mathit{C}H(C_6H_{13})_2)$, 54.7 ($\mathit{C}H(C_6H_{13})_2)$, 35.5 ($\mathit{C}(CH_3)_3)$, 35.0 ($\mathit{C}(CH_3)_3)$ 32.5 ($CH(\mathit{C}H_2CH_2CH_2CH_2CH_2CH_3)_2)$, 32.4 ($CH(\mathit{C}H_2CH_2CH_2CH_2CH_2CH_3)_2)$, 32.1 ($\mathit{C}(CH_3)_3)$, 31.9 ($CH(\mathit{C}H_2\mathit{C}H_2CH_2CH_2CH_2CH_3)_2)$, 31.9 ($CH(CH_2\mathit{C}H_2CH_2CH_2CH_2CH_3)_2)$, 31.5 ($\mathit{C}(CH_3)_3)$, 29.4 ($CH(CH_2CH_2\mathit{C}H_2CH_2CH_2CH_3)_2)$, 29.3 ($CH(CH_2CH_2\mathit{C}H_2CH_2CH_2CH_3)_2)$, 27.1 ($CH(CH_2CH_2CH_2\mathit{C}H_2CH_2CH_3)_2)$, 26.9 ($CH(CH_2CH_2CH_2\mathit{C}H_2CH_2CH_3)_2)$, 22.7 ($CH(CH_2CH_2CH_2CH_2\mathit{C}H_2CH_3)_2)$, 22.7 ($CH(CH_2CH_2CH_2CH_2\mathit{C}H_2CH_3)_2)$, 14.2 ($CH(CH_2CH_2CH_2CH_2CH_2\mathit{C}H_3)_2$, 14.2 ($CH(CH_2CH_2CH_2CH_2CH_2\mathit{C}H_3)_2$. **HRMS** (MALDI-TOF): $[M+Ag]^+$ Calculated for $C_{74}H_{86}N_2O_4Ag$, 1173.5629; found, 1173.5621.

Compound E. Compound **D** (2.39 g, 2.24 mmol, 1 eq.) was dissolved in anhydrous CH_2Cl_2 (100 mL) in an oven dried 250 mL round-bottom flask. Subsequently, boron trifluoride diethyl etherate ($BF_3{\cdot}Et_2O$) (1.10 mL, 1.27 g, 8.96 mmol, 4 eq.) was added. The reaction mixture was deaerated using vacuum and backfilled with nitrogen three times. Thereafter, the flask was sealed using a rubber septum. The solution was cooled to 0 °C in an ice bath for about 20 minutes and DDQ (1.02 g, 4.48 mmol, 2 eq.) was added. The reaction mixture was stirred at room temperature for 1.5 h under an inert atmosphere. After ensuring the completion of the reaction by TLC analysis, reaction was quenched by the addition of a saturated aqueous solution of $NaHCO_3$ (100 mL). The CH_2Cl_2 solution was extracted using a separatory funnel and washed with water (2 x 100 mL). Finally, the organic phase was collected and concentrated using rotary evaporation. The solid residue was washed with methanol and

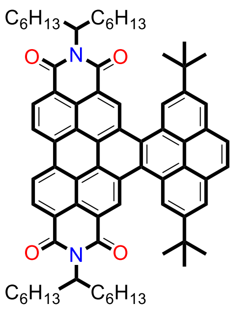

dried to obtain the pure product **E** (2.37 g, 99%) as dark-red amorphous solid. **^{1}H NMR** (500 MHz, $CDCl_3$): δ 10.51 (br s, 1H, $C\mathit{H}_{Ar}$), 10.46 (br s, 1H, $C\mathit{H}_{Ar}$), 9.38 (d, J = 5.0 Hz, 2H, $C\mathit{H}_{Ar}$), 9.10 (br s, 4H, $C\mathit{H}_{Ar}$), 8.40 (s, 2H, $C\mathit{H}_{Ar}$), 8.19 (s, 2H, $C\mathit{H}_{Ar}$), 5.44–5.32 (m, 2H, $C\mathit{H}(C_6H_{13})_2$), 2.45–2.31 (m, 4H, $CH(C\mathit{H}_2CH_2CH_2CH_2CH_2CH_3)_2$), 2.00–1.88 (m, 4H, $CH(C\mathit{H}_2CH_2CH_2CH_2CH_2CH_3)_2$), 1.70 (s, 18H, $C(C\mathit{H}_3)_3$), 1.51–1.19 (m, 32H, $CH(CH_2C\mathit{H}_2C\mathit{H}_2C\mathit{H}_2C\mathit{H}_2CH_3)_2$), 0.83 (t, $^{3}J_{H\text{-}H}$ = 8Hz, 12H, $CH(CH_2CH_2CH_2CH_2CH_2C\mathit{H}_3)_2$)). **^{13}C{^{1}H} NMR** (126 MHz, $CDCl_3$): δ 165.7 (C=O), 165.6 (C=O), 164.7 (C=O), 164.4 (C=O), 149.6 (C_{Ar}), 135.2 (C_{Ar}), 134.4 (C_{Ar}), 133.8 (C_{Ar}), 131.4 (C_{Ar}), 129.9 (C_{Ar}), 129.6 (C_{Ar}), 128.9 (C_{Ar}), 128.0 (C_{Ar}), 127.6 (C_{Ar}), 127.4 (C_{Ar}), 127.4 (C_{Ar}), 127.3 (C_{Ar}), 124.7 (C_{Ar}), 124.7 (C_{Ar}), 124.1 (C_{Ar}), 123.8 (C_{Ar}), 123.3 (C_{Ar}), 55.0 ($\mathit{C}H(C_6H_{13})_2$), 54.8 ($\mathit{C}H(C_6H_{13})_2$), 35.7 ($\mathit{C}(CH_3)_3$), 32.7 ($CH(\mathit{C}H_2CH_2CH_2CH_2CH_2CH_3)_2$), 32.6 ($CH(CH_2\mathit{C}H_2CH_2CH_2CH_2CH_3)_2$), 32.0 ($\mathit{C}(CH_3)_3$), 29.4 ($CH(CH_2CH_2\mathit{C}H_2CH_2CH_2CH_3)_2$), 27.1($CH(CH_2CH_2CH_2\mathit{C}H_2CH_2CH_3)_2$), 22.8 ($CH(CH_2CH_2CH_2CH_2\mathit{C}H_2CH_3)_2$), 14.2 ($CH(CH_2CH_2CH_2CH_2CH_2\mathit{C}H_3)_2$). **HRMS** (MALDI-TOF): $[M+Ag]^{+}$ Calculated for $C_{74}H_{84}N_2O_4Ag$, 1171.5473; found, 1171.5491.

PBI-pyrene-diketone 1. A mixture of compound **E** (1.50 g, 1.41 mmol, 1 eq.), $RuCl_3xH_2O$ (0.11 g, 0.54 mmol, 0.38 eq.), and $NaIO_4$ (1.35 g, 6.31 mmol, 4.5 eq.) was taken in a 1L round-bottomed flask. Subsequently, CH_2Cl_2 (120 mL), acetonitrile (120 mL), and distilled water (120 mL) were added. The resultant mixture was stirred at 65 °C for 24 h and then allowed to cool to room temperature. The organic phase was collected using a separatory funnel and washed with water (2 x 200 mL). The solvents were removed by evaporation under reduced pressure. The solid residue was purified by silica-gel column chromatography (1:1 CH_2Cl_2-hexane) to achieve the product (0.77 g, 50%) as a red crystalline solid. Important to notice that the unreacted starting compound **E** (0.22 g, 15%) was recovered from the column. **^{1}H NMR** (500 MHz, $CDCl_3$): δ 10.29-10.14 (m, 2H, $C\mathit{H}_{Ar}$), 9.41 (d, J = 5.0 Hz, 2H, $C\mathit{H}_{Ar}$), 9.21-9.10 (m, 2H, $C\mathit{H}_{Ar}$), 9.10-9.5 (m, 2H, $C\mathit{H}_{Ar}$), 8.74 (s, 2H, $C\mathit{H}_{Ar}$), 5.42–5.20 (m, 2H, $C\mathit{H}(C_6H_{13})_2$), 2.42–2.30 (m, 4H, $CH(C\mathit{H}_2CH_2CH_2CH_2CH_2CH_3)_2$), 1.98–1.86 (m, 4H, $CH(C\mathit{H}_2CH_2CH_2CH_2CH_2CH_3)_2$), 1.59 (s, 18H, $C(C\mathit{H}_3)_3$), 1.47–1.17 (m, 32H, $CH(CH_2C\mathit{H}_2C\mathit{H}_2C\mathit{H}_2C\mathit{H}_2CH_3)_2$), 0.81 (t, $^{3}J_{H\text{-}H}$ = 8 Hz, 12H, $CH(CH_2CH_2CH_2CH_2CH_2C\mathit{H}_3)_2$). **^{13}C{^{1}H} NMR** (126 MHz, $CDCl_3$): δ

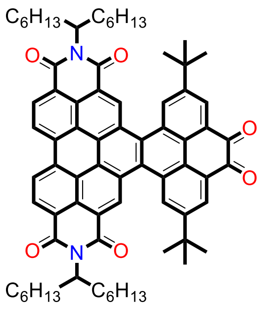

180.7 (C=$O_{diketone}$), 165.4 (C=O),165.4 (C=O), 164.4 (C=O), 164.1 (C=O), 152.2 (C_{Ar}), 135.1 (C_{Ar}), 134.1 (C_{Ar}), 133.9 (C_{Ar}), 133.2 (C_{Ar}), 130.3 (C_{Ar}), 130.1 (C_{Ar}), 129.6 (C_{Ar}), 128.9 (C_{Ar}), 128.9 (C_{Ar}), 128.2 (C_{Ar}), 128.0 (C_{Ar}), 127.4 (C_{Ar}), 126.7 (C_{Ar}), 125.4 (C_{Ar}), 124.6 (C_{Ar}), 123.7 (C_{Ar}), 55.1 (CH$(C_6H_{13})_2$), 35.8 ($C(CH_3)_3$), 32.6 ($CH(\mathit{C}H_2CH_2CH_2CH_2CH_2CH_3)_2$), 31.9 ($CH(CH_2\mathit{C}H_2CH_2CH_2CH_2CH_3)_2$), 31.3 ($C(CH_3)_3$), 29.4 ($CH(CH_2CH_2\mathit{C}H_2CH_2CH_2CH_3)_2$), 27.1($CH(CH_2CH_2CH_2\mathit{C}H_2CH_2CH_3)_2$), 22.7 ($CH(CH_2CH_2CH_2CH_2\mathit{C}H_2CH_3)_2$), 14.2 ($CH(CH_2CH_2CH_2CH_2CH_2\mathit{C}H_3)_2$). **HRMS** (MALDI-TOF): $[M+Ag]^+$ Calculated for $C_{74}H_{82}N_2O_6Ag$, 1201.5215; found, 1201.5267.

2. Synthesis and characterization of the ligand precursor

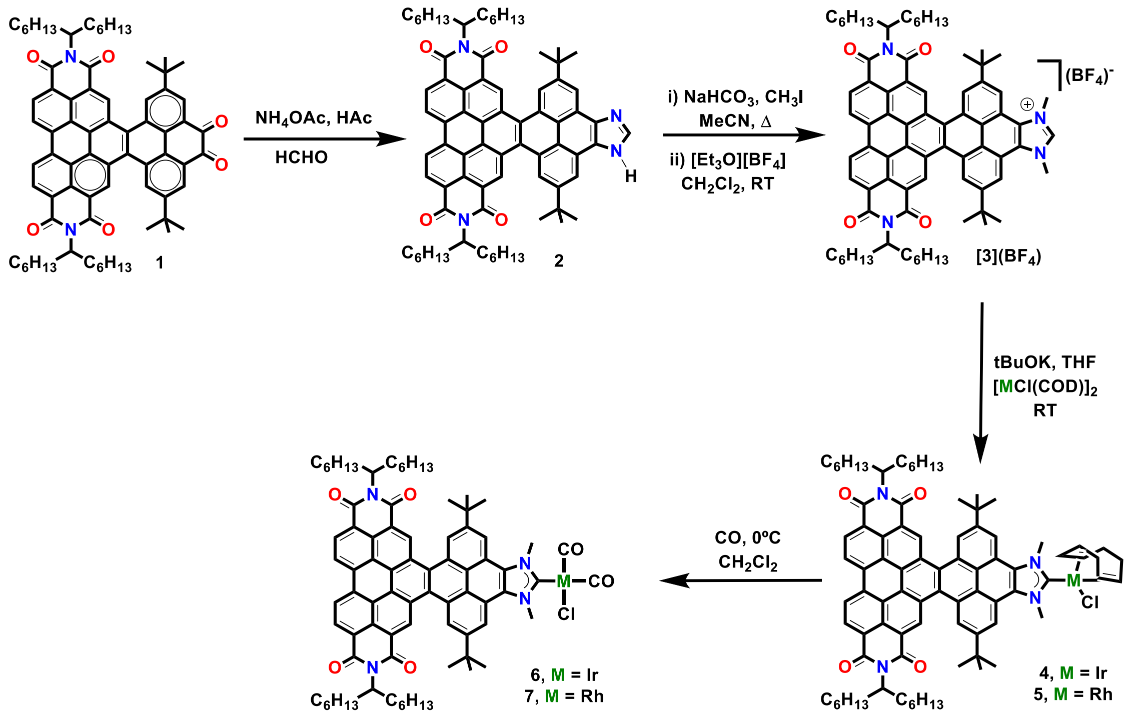

Scheme S2. Synthesis of the ligand precursor and the metal complexes

Compound 2. A mixture of diketone **1** (150 mg, 0.14 mmol, 1 eq.), ammonium acetate (220 mg, 2.80 mmol, 20 eq.) and acetic acid (5 mL) was stirred at 90 ºC until a complete solution of the solids (10 min approximately). After this time, formaldehyde (13 µL, 1.168 mmol, 1.2 eq.) was added to the reaction and the resulting dark solution was refluxed overnight. After cooling at room temperature, water (10 mL) was added to the mixture and the suspension was neutralized with NH_4OH (28-30%). The so-formed solid was collected by filtration, washed with water, and dried under vacuum. Compound **2** (150 mg, 99%) was isolated as a dark maroon solid. **^{1}H NMR** (400 MHz, $CDCl_3$): δ 10.58-10.33 (m, 2H, CH_{Ar}), 9.40 (d, J = 5.0 Hz, 2H, CH_{Ar}), 9.22-8.67 (m, 6H, CH_{Ar}), 8.43 (s, 1H, NC*H*N), 5.42-5.24 (m, 2H, C*H*$(C_6H_{13})_2$), 2.49-2.29 (m, 4H, CH$(CH_2CH_2CH_2CH_2CH_2CH_3)_2$), 2.04-1.86 (m, 4H, CH$(CH_2CH_2CH_2CH_2CH_2CH_3)_2$), 1.66 (s, 18H, C$(CH_3)_3$), 1.50-1.14 (m, 32H, CH$(CH_2CH_2CH_2CH_2CH_2CH_3)_2$), 0.93-0.71 (m, 12H, CH$(CH_2CH_2CH_2CH_2CH_2CH_3)_2$). **^{13}C{^{1}H} NMR** (100 MHz, $CDCl_3$): δ 165.6 (*C*=O),165.4 (*C*=O), 164.6 (*C*=O), 163.8 (*C*=O), 149.9 (C_{Ar}), 138.3 (N*C*HN), 135.3 (C_{Ar}), 134.5 (C_{Ar}), 134.2 (C_{Ar}), 133.8 (C_{Ar}), 130.2 (C_{Ar}), 129.9 (C_{Ar}) 129.6 (C_{Ar}), 128.9 (C_{Ar}), 128.2 (C_{Ar}), 127.5 (C_{Ar}), 127.2 (C_{Ar}), 126.8 (C_{Ar}), 126.5 (C_{Ar}), 124.8 (C_{Ar}), 124.6 (C_{Ar}),

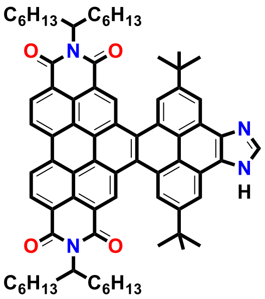

123.2 (C_{Ar}) 121.9 (C_{Ar}), 54.9 ($CH(C_6H_{13})_2$), 35.8 ($C(CH_3)_3$), 32.6 ($CH(CH_2CH_2CH_2CH_2CH_2CH_3)_2$), 31.9 ($CH(CH_2CH_2CH_2CH_2CH_2CH_3)_2$), 31.9 ($C(CH_3)_3$), 29.4 ($CH(CH_2CH_2CH_2CH_2CH_2CH_3)_2$), 27.1 ($CH(CH_2CH_2CH_2CH_2CH_2CH_3)_2$), 22.7 ($CH(CH_2CH_2CH_2CH_2CH_2CH_3)_2$), 14.2 ($CH(CH_2CH_2CH_2CH_2CH_2CH_3)_2$).

Imidazolium salt [3](BF$_4$). A mixture of **2** (150 mg, 0.14 mmol, 1 eq.), $NaHCO_3$ (24 mg, 0.28 mmol, 2 eq.) and acetonitrile (3 mL) was placed together in a Schlenk tube fitted with a Teflon cap. The solution was stirred at 90ºC for 1h. Then, the reaction was allowed to reach room temperature and CH_3I (53 µL, 0.84 mmol, 6 eq.) was added. The resulting solution was heated at 90ºC overnight. After removal of the volatiles, the crude solid was placed in a Schlenk flask containing

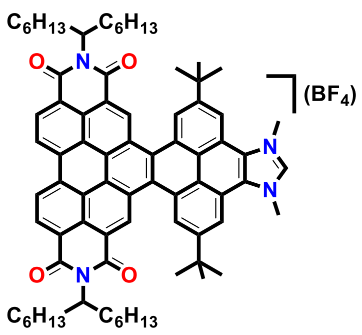

$(Et_3O)(BF_4)$ (27 mg, 0.14 mmol, 1 eq.). Dry and previously deoxygenated CH_2Cl_2 (10 mL) was added. The resulting mixture was stirred at room temperature for 45 min and a colour change from dark maroon to bright orange was observed. After this time, the solvent was removed under reduced pressure, giving the desired imidazolium salt [**3**](BF_4) (141 mg, 85 %).**^{1}H NMR** (300 MHz, $CDCl_3$): δ 10.45-10.32 (m, 2H, CH_{Ar}), 9.74 (s, 1H, NC*H*N), 9.45 (s, 1H, CH_{Ar}), 9.42 (s, 1H, CH_{Ar}), 9.27 (br. s, 2H, CH_{Ar}), 9.21-9.12 (m, 2H, CH_{Ar}), 9.05 (s, 2H, CH_{Ar}), 5.42-5.29 (m, 2H, $CH(C_6H_{13})_2$), 4.95 (s, 6H, NCH_3), 2.47-2.30 (m, 4H, $CH(CH_2CH_2CH_2CH_2CH_2CH_3)_2$), 2.01-1.85 (m, 4H, $CH(CH_2CH_2CH_2CH_2CH_2CH_3)_2$), 1.74 (s, 18H, $C(CH_3)_3$), 1.46-1.18 (m, 32 H, $CH(CH_2CH_2CH_2CH_2CH_2CH_3)_2$), 0.82 (t, $^3J_{H-H}$ = 8 Hz, 12H, $CH(CH_2CH_2CH_2CH_2CH_2CH_3)_2$). **^{13}C{^{1}H} NMR** (75 MHz, $CDCl_3$): δ 165.5 (*C*=O), 165.4 (*C*=O), 164.4 (*C*=O), 164.1 (*C*=O), 150.9 (C_{Ar}), 142.9 (N*C*HN), 134.3 (C_{Ar}), 133.8 (C_{Ar}), 133.5 (C_{Ar}), 130.0 (C_{Ar}), 129.4 (C_{Ar}) 129.1 (C_{Ar}), 129.1 (C_{Ar}), 128.8 (C_{Ar}), 127.9 (C_{Ar}), 127.5 (C_{Ar}), 126.7 (C_{Ar}), 125.4 (C_{Ar}), 124.6 (C_{Ar}), 123.6 (C_{Ar}), 122.8 (C_{Ar}), 120.7 (C_{Ar}), 117.5 (C_{Ar}), 55.0 ($CH(C_6H_{13})_2$), 39.0 (NCH_3), 36.0 ($C(CH_3)_3$), 32.6 ($CH(CH_2CH_2CH_2CH_2CH_2CH_3)_2$), 31.9 ($CH(CH_2CH_2CH_2CH_2CH_2CH_3)_2$), 31.8 ($C(CH_3)_3$), 29.4 ($CH(CH_2CH_2CH_2CH_2CH_2CH_3)_2$), 27.1 ($CH(CH_2CH_2CH_2CH_2CH_2CH_3)_2$), 22.7 ($CH(CH_2CH_2CH_2CH_2CH_2CH_3)_2$), 14.2 ($CH(CH_2CH_2CH_2CH_2CH_2CH_3)_2$. **HRMS**: 1133.6893 [M – (BF_4)]$^+$. (Calcd. for [M – (BF_4)]$^+$: 1133.6884).

3. Synthesis and characterization of the complexes

General procedure for the preparation of complexes 4 and 5. A mixture of salt [**3**](BF_4) (1 eq.), *t*BuOK (2 eq.) and the corresponding metal precursor [MCl(cod)]$_2$ (M = Rh or Ir) (0.5 eq.) were placed together in a Schlenk flask. Dry THF was added and the resulting mixture was stirred at room temperature overnight. The solvent was then removed under reduced pressure. The crude product was purified by column chromatography using CH_2Cl_2 as eluent.

Iridium complex 4. Complex **4** was obtained by reacting [**3**](BF_4) (50 mg, 0.04 mmol), [IrCl(cod)]$_2$ (13.4 mg, 0.02 mmol) and *t*BuOK (9.2 mg, 0.08 mmol) in THF (5 mL). After purification by column chromatography, complex **4** (39 mg, 65%) was isolated as a dark maroon solid. **^{1}H NMR** (300 MHz, $CDCl_3$): δ 10.50-10.34 (m, 2H, CH_{Ar}), 9.42 (s, 1H, CH_{Ar}), 9.39 (s, 1H, CH_{Ar}), 9.20-9.03 (m, 6H, CH_{Ar}), 5.42-5.29 (m, 2H, CH(C_6H_{13})$_2$), 5.10 (s, 6H, NCH_3), 4.92-4.82 (m, 2H, CH_{cod}), 3.29-3.17 (m, 2H, CH_{cod}), 2.51-2.30 (m, 8H, CH_2 $_{cod}$ and CH(CH_2CH$_2$CH$_2$CH$_2$CH$_2$CH$_3$)$_2$), 2.03-1.86 (m, 8H, CH_2 $_{cod}$ and CH(CH_2CH$_2$CH$_2$CH$_2$CH$_2$CH$_3$)$_2$), 1.71 (s, 18H, C(CH_3)$_3$), 1.45-1.15 (m, 32H, CH(CH$_2$CH_2CH_2CH_2CH_2CH$_3$)$_2$), 0.87-0.80 (m, 12H, CH(CH$_2$CH$_2$CH$_2$CH$_2$CH$_2$CH_3)$_2$). **^{13}C{^{1}H} NMR** (75 MHz, $CDCl_3$): δ 188.1 (Ir-$C_{carbene}$), 165.5 (*C*=O), 165.5 (*C*=O), 164.5 (*C*=O), 164.2 (*C*=O), 149.7 (C_{Ar}), 134.9 (C_{Ar}), 134.2 (C_{Ar}), 133.8 (C_{Ar}), 129.8 (C_{Ar}), 129.6 (C_{Ar}) 129.3 (C_{Ar}), 129.1 (C_{Ar}), 128.3 (C_{Ar}), 127.5 (C_{Ar}), 127.0 (C_{Ar}), 126.9 (C_{Ar}), 125.0 (C_{Ar}), 124.6 (C_{Ar}), 123.4 (C_{Ar}), 121.6 (C_{Ar}), 121.6 (C_{Ar}), 117.3 (C_{Ar}), 86.2 (CH$_{cod}$), 55.0 (CH(C_6H_{13})$_2$), 52.6 (CH$_{cod}$), 40.3 (NCH$_3$), 35.9 (C(CH$_3$)$_3$), 33.8 (CH$_2$ $_{cod}$), 32.6 (CH(CH$_2$CH$_2$CH$_2$CH$_2$CH$_2$CH$_3$)$_2$), 31.9 (CH(CH$_2$$CH_2CH_2CH_2CH_2CH_3$)$_2$), 31.8 ($C$(CH$_3$)$_3$), 29.8 (CH(CH$_2CH_2$$CH_2CH_2CH_2CH_3$)$_2$), 29.4 ($CH_2$ $_{cod}$) 27.1 (CH(CH$_2$CH$_2$CH$_2$$CH_2CH_2CH_3$)$_2$), 22.7 (CH(CH$_2CH_2CH_2CH_2$$CH_2CH_3$)$_2$), 14.2 (CH(CH$_2CH_2CH_2CH_2CH_2$$CH_3$)$_2$).

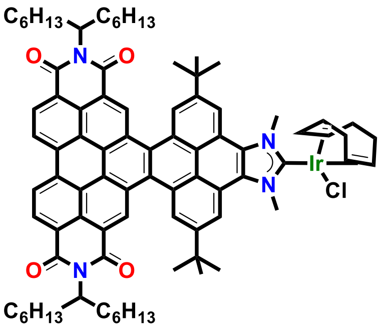

Rhodium complex 5. Complex **5** was obtained by reacting [**3**](BF_4) (50 mg, 0.04 mmol), [RhCl(cod)]$_2$ (9.9 mg, 0.02 mmol) and *t*BuOK (9.2 mg, 0.08 mmol) in THF (5 mL). After purification by column chromatography using CH_2Cl_2 as eluent, complex **5** (25 mg, 45%) was isolated as a dark maroon solid. **^{1}H NMR** (300 MHz, $CDCl_3$): δ 10.49-10.34 (m, 2H, CH_{Ar}), 9.42 (s, 1H, CH_{Ar}), 9.39 (s, 1H, CH_{Ar}), 9.22-9.09 (m, 4H, CH_{Ar}), 9.05 (s, 2H,

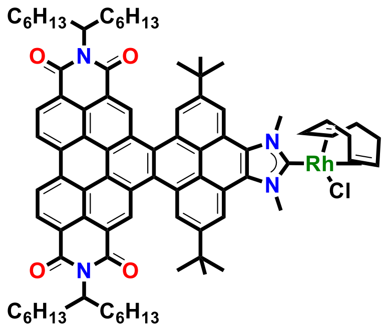

CH_{Ar}), 5.43-5.32 (m, 2H, CH(C_6H_{13})$_2$), 5.32-5.28 (m, 2H, CH_{cod}), 5.24 (s, 6H, NCH_3), 3.66-3.57 (m, 2H, CH_{cod}), 2.69-2.50 (m, 4H, CH_2 $_{cod}$), 2.50-2.29 (m, 4H, CH(CH_2CH$_2$CH$_2$CH$_2$CH$_2$CH$_3$)$_2$), 2.20-2.07 (m, 4H, CH_2 $_{cod}$), 2.02-1.85 (m, 4H, CH(CH_2CH$_2$CH$_2$CH$_2$CH$_2$CH$_3$)$_2$), 1.71 (s, 18H, C(CH_3)$_3$), 1.44-1.18 (m, 32H, CH(CH$_2$CH_2CH_2CH_2CH_2CH$_3$)$_2$), 0.90-0.76 (m, 12H, CH(CH$_2$CH$_2$CH$_2$CH$_2$CH$_2$CH_3)$_2$). **^{13}C{^{1}H} NMR** (100 MHz, CDCl$_3$): δ 192.2 (d, Rh-$C_{carbene}$, $^1J_{Rh\text{-}C}$ = 50 Hz), 165.5 (*C*=O), 165.5 (*C*=O), 164.5 (*C*=O), 164.3 (*C*=O), 149.7 (C_{Ar}), 134.9 (C_{Ar}), 134.1 (C_{Ar}), 133.8 (C_{Ar}), 129.6 (C_{Ar}), 129.5 (C_{Ar}) 129.1 (C_{Ar}), 128.8 (C_{Ar}), 128.3 (C_{Ar}), 127.5 (C_{Ar}), 126.9 (C_{Ar}), 126.9 (C_{Ar}), 125.0 (C_{Ar}), 124.6 (C_{Ar}), 123.4 (C_{Ar}), 121.5 (C_{Ar}), 121.5 (C_{Ar}), 117.3 (C_{Ar}), 99.9 (d, Rh-CH$_{cod}$, $^1J_{Rh\text{-}C}$ = 5.9 Hz), 68.95 (d, Rh-CH$_{cod}$, $^1J_{Rh\text{-}C}$ = 10.0 Hz), 55.0 (CH(C_6H_{13})$_2$), 40.6 (NCH$_3$), 35.9 (C(CH$_3$)$_3$), 33.2 (CH$_2$ $_{cod}$), 32.6 (CH(CH$_2$CH$_2$CH$_2$CH$_2$CH$_2$CH$_3$)$_2$), 31.9 (CH(CH$_2$$CH_2CH_2CH_2CH_2CH_3$)$_2$), 31.8 (C($CH_3$)$_3$), 29.4 (CH($CH_2CH_2$$CH_2CH_2CH_2CH_3$)$_2$), 29.2 ($CH_2$ $_{cod}$) 27.1 (CH(CH$_2$CH$_2$CH$_2$$CH_2CH_2CH_3$)$_2$), 22.7 (CH(CH$_2CH_2CH_2CH_2$$CH_2CH_3$)$_2$), 14.2 (CH(CH$_2CH_2CH_2CH_2CH_2$$CH_3$)$_2$). **HRMS**: 1385.7126 [M – (Cl) + (MeCN)]$^+$. (Calcd. for [M – (Cl) + (MeCN)]$^+$: 1185.7175).

General procedure for the preparation of the carbonyl derivatives 6 and 7. CO gas (1 atm, 10mL/min) was passed through a solution of complexes **4** and **5** in CH_2Cl_2 (10 mL) at 0ºC for 45 minutes. An immediate colour change from dark maroon to bright red was observed. The solvent was removed under reduced pressure, giving the desired carbonyl derivatives **6** and **7**, respectively, in high yields.

Iridium complex 6. Complex **6** was obtained following the general procedure. It was isolated as a red solid in 94% yield. **^{1}H NMR** (300 MHz, CDCl$_3$): δ 10.46-10.35 (m, 2H, CH_{Ar}), 9.43 (s, 1H, CH_{Ar}), 9.40 (s, 1H, CH_{Ar}), 9.14 (br. s, 4H, CH_{Ar}), 9.06 (s, 2H, CH_{Ar}), 5.43-5.27 (m, 2H, CH(C_6H_{13})$_2$), 4.98 (s, 6H, NCH_3), 2.48-2.30 (m, 4H, CH(CH_2CH$_2$CH$_2$CH$_2$CH$_2$CH$_3$)$_2$), 2.02-1.87 (m, 4H, CH(CH_2CH$_2$CH$_2$CH$_2$CH$_2$CH$_3$)$_2$), 1.72 (s, 18H, C(CH_3)$_3$), 1.46-1.16 (m, 32H, CH(CH$_2$CH_2CH_2CH_2CH_2CH$_3$)$_2$), 0.94-0.75 (m, 12H, CH(CH$_2$CH$_2$CH$_2$CH$_2$CH$_2$CH_3)$_2$). **^{13}C{^{1}H} NMR** (75 MHz, CDCl$_3$): δ 181.1 (Ir-C_{CO}),

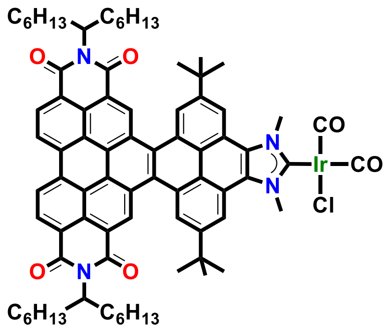

180.4 (Ir-$C_{carbene}$), 168.0 (Ir-C=O), 165.5 (C=O), 165.5 (C=O), 164.5 (C=O), 164.3 (C=O), 150.0 (C_{Ar}), 134.7 (C_{Ar}), 133.9 (C_{Ar}), 133.8 (C_{Ar}), 130.0 (C_{Ar}), 129.4 (C_{Ar}) 129.4 (C_{Ar}), 128.5 (C_{Ar}), 127.7 (C_{Ar}), 127.5 (C_{Ar}), 126.8 (C_{Ar}), 125.2 (C_{Ar}), 124.6 (C_{Ar}), 123.5 (C_{Ar}), 122.2 (C_{Ar}), 122.1 (C_{Ar}), 121.5(C_{Ar}), 117.4 (C_{Ar}), 55.0 (CH($C_6H_{13})_2$), 41.1 (NCH$_3$), 35.9 (C(CH$_3$)$_3$), 32.6 (CH(CH$_2$CH$_2$CH$_2$CH$_2$CH$_2$CH$_3$)$_2$), 31.9 (CH(CH$_2$$CH_2CH_2CH_2CH_2CH_3$)$_2$), 31.8 (C($CH_3$)$_3$), 29.4 ((CH($CH_2CH_2$$CH_2CH_2CH_2CH_3$)$_2$), 27.1 (CH(CH$_2CH_2CH_2$$CH_2CH_2CH_3$)$_2$), 22.7 (CH(CH$_2CH_2CH_2CH_2$$CH_2CH_3$)$_2$), 14.2 (CH(CH$_2CH_2CH_2CH_2CH_2$$CH_3$)$_2$). **IR** ($CH_2Cl_2$): 1988 ν(Ir-CO) and 2071 ν(Ir-CO) cm^{-1}.

Rhodium complex 7. Complex **7** was obtained following the general procedure. It was isolated as a red solid in 90% yield. **^{1}H NMR** (300 MHz, CDCl$_3$): δ 10.51-10.31 (m, 2H, CH_{Ar}), 9.43 (s, 1H, CH_{Ar}), 9.40 (s, 1H, CH_{Ar}), 9.19-9.09 (m, 4H, CH_{Ar}), 9.06 (s, 2H, CH_{Ar}), 5.43-5.27 (m, 2H, CH($C_6H_{13})_2$), 4.90 (s, 6H, NCH_3), 2.49-2.31 (m, 4H, CH(CH_2CH$_2$CH$_2$CH$_2$CH$_2$CH$_3$)$_2$), 2.01-1.89 (m, 4H, CH(CH_2CH$_2$CH$_2$CH$_2$CH$_2$CH$_3$)$_2$), 1.73 (s, 18H, C(CH_3)$_3$), 1.47-1.15 (m, 32H, CH(CH$_2$CH_2CH_2CH_2CH_2CH$_3$)$_2$), 0.96-0.72 (m, 12H, CH(CH$_2$CH$_2$CH$_2$CH$_2$CH$_2$CH_3)$_2$). **^{13}C{^{1}H} NMR** (75 MHz, CDCl$_3$): δ 187.3 (d, Rh-C_{CO}, $^1J_{Rh\text{-}C}$ = 53 Hz), 181.5 (d, Rh-C_{CO}, $^1J_{Rh\text{-}C}$ = 76 Hz), 180.7 (d, Rh-$C_{carbene}$, $^1J_{Rh\text{-}C}$ = 41 Hz) 165.5 (C=O), 165.4 (C=O), 164.5 (C=O), 164.4 (C=O), 150.0 (C_{Ar}), 134.8 (C_{Ar}), 134.2 (C_{Ar}), 133.8 (C_{Ar}), 130.0 (C_{Ar}), 129.5 (C_{Ar}), 129.2 (C_{Ar}), 128.5 (C_{Ar}), 128.4 (C_{Ar}), 127.5 (C_{Ar}), 126.9 (C_{Ar}), 125.2 (C_{Ar}), 124.6 (C_{Ar}), 123.4 (C_{Ar}), 122.6 (C_{Ar}), 129.9 (C_{Ar}), 121.4 (C_{Ar}), 117.4 (C_{Ar}), 55.0 (CH($C_6H_{13})_2$), 41.1 (NCH$_3$), 35.9 (C(CH$_3$)$_3$), 32.6 (CH(CH$_2$CH$_2$CH$_2$CH$_2$CH$_2$CH$_3$)$_2$), 31.9 (CH(CH$_2$$CH_2CH_2CH_2CH_2CH_3$)$_2$), 31.8 (C($CH_3$)$_3$), 9.4 (CH($CH_2CH_2$$CH_2CH_2CH_2CH_3$)$_2$), 27.1 (CH(CH$_2CH_2CH_2$$CH_2CH_2CH_3$)$_2$), 22.7 (CH(CH$_2CH_2CH_2CH_2$$CH_2CH_3$)$_2$), 14.2 (CH(CH$_2CH_2CH_2CH_2CH_2$$CH_3$)$_2$). **IR** ($CH_2Cl_2$): 2006 ν(Rh-CO) and 2077 ν(Rh-CO) cm^{-1}.

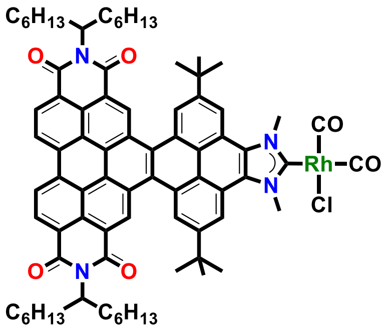

4. Spectroscopic data

4.1. ^{1}H and ^{13}C spectra of D in $CDCl_3$

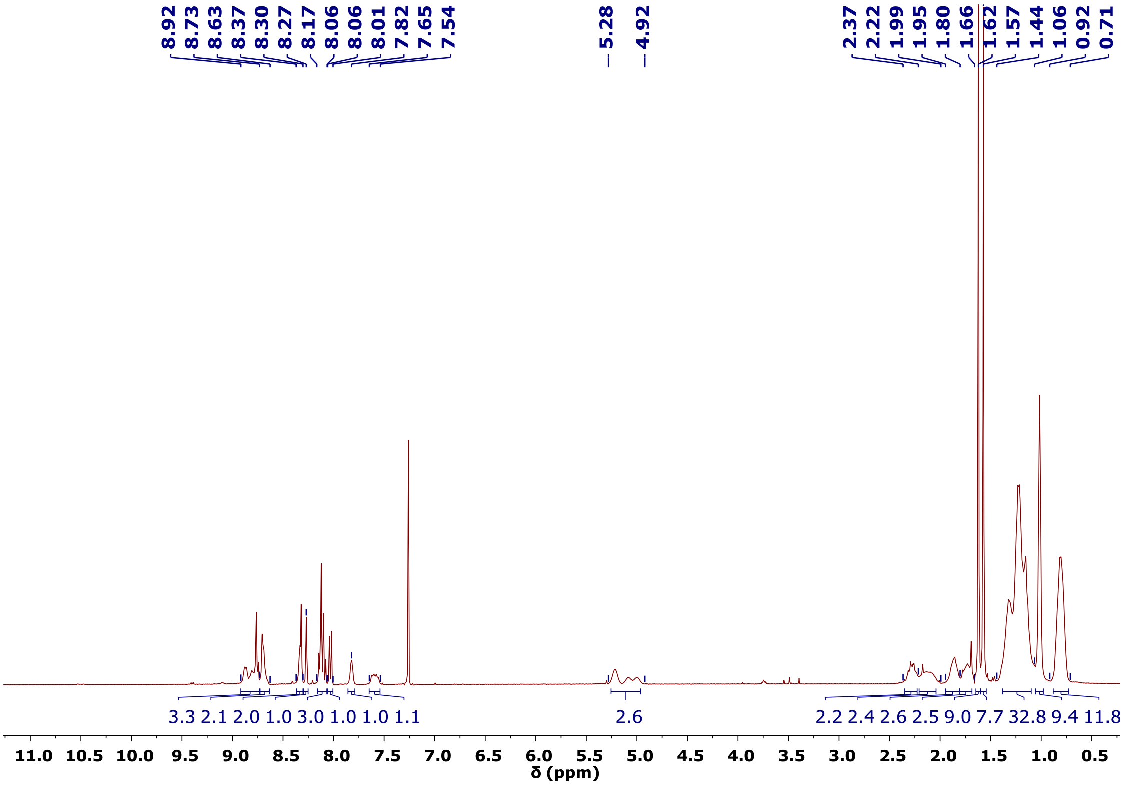

Figure S1. ^{1}H NMR spectrum (400 MHz, $CDCl_3$) of **D**

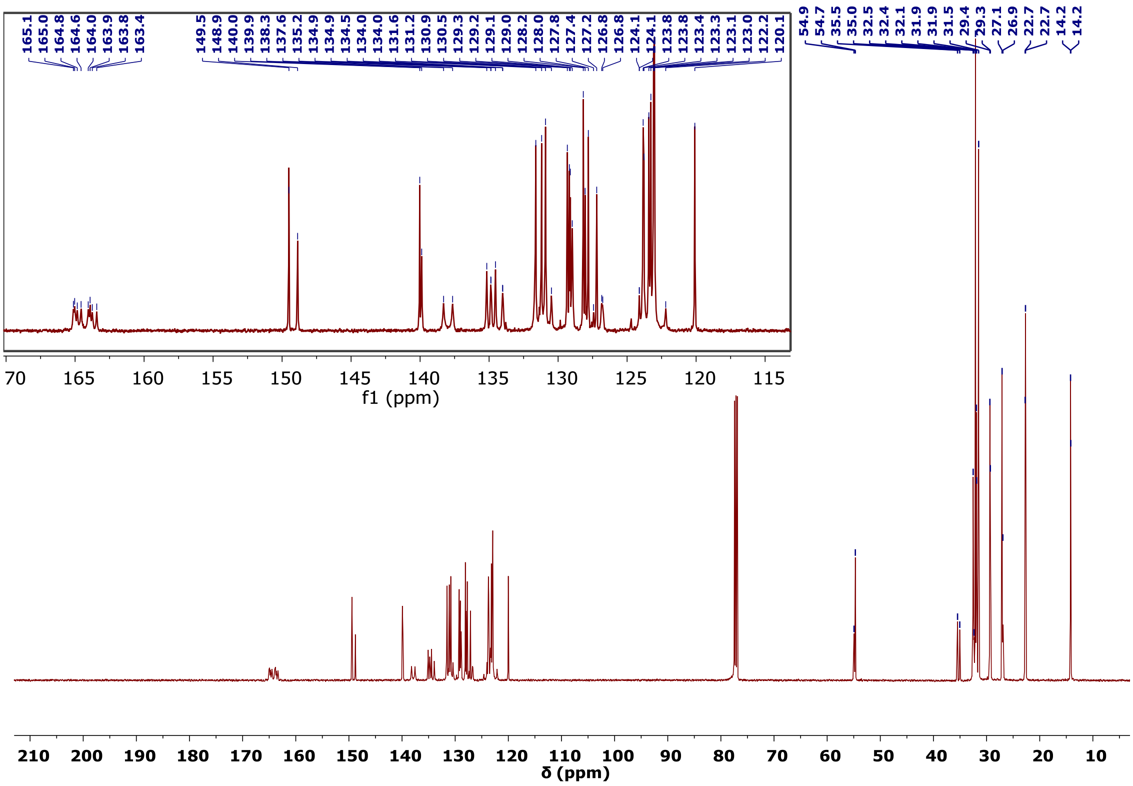

Figure S2. $^{13}C\{^1H\}$ spectrum (126 MHz, $CDCl_3$) of **D**

4.2. ^{1}H and ^{13}C spectra of E in $CDCl_3$

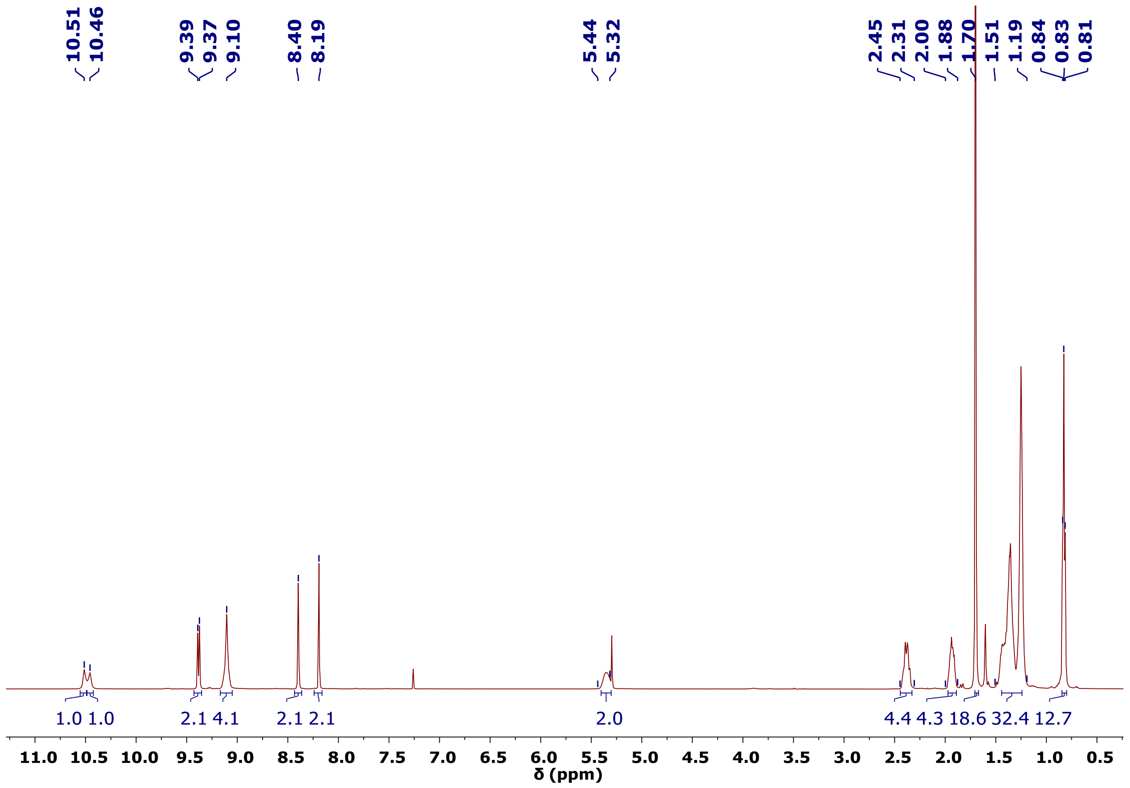

Figure S3. ^{1}H NMR spectrum (500 MHz, $CDCl_3$) of **E**

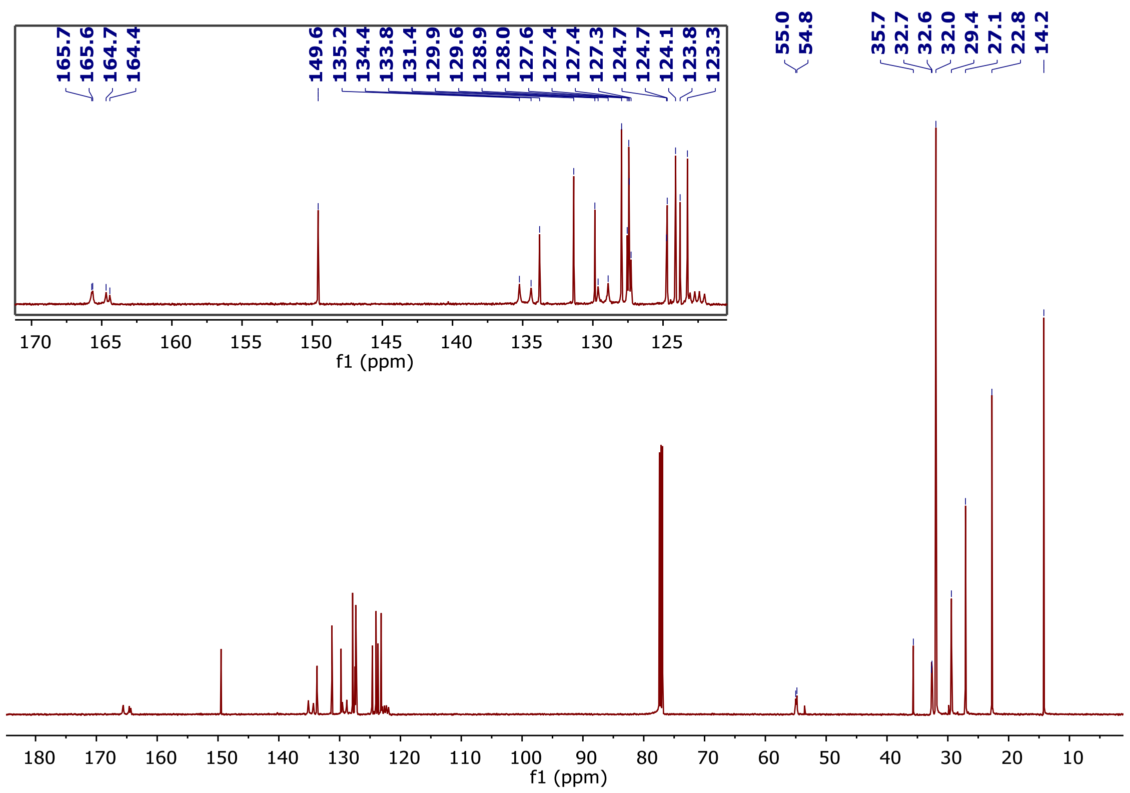

Figure S4. $^{13}C\{^{1}H\}$ spectrum (126 MHz, $CDCl_3$) of **E**

4.3. ^{1}H and ^{13}C spectra of 1 in $CDCl_3$

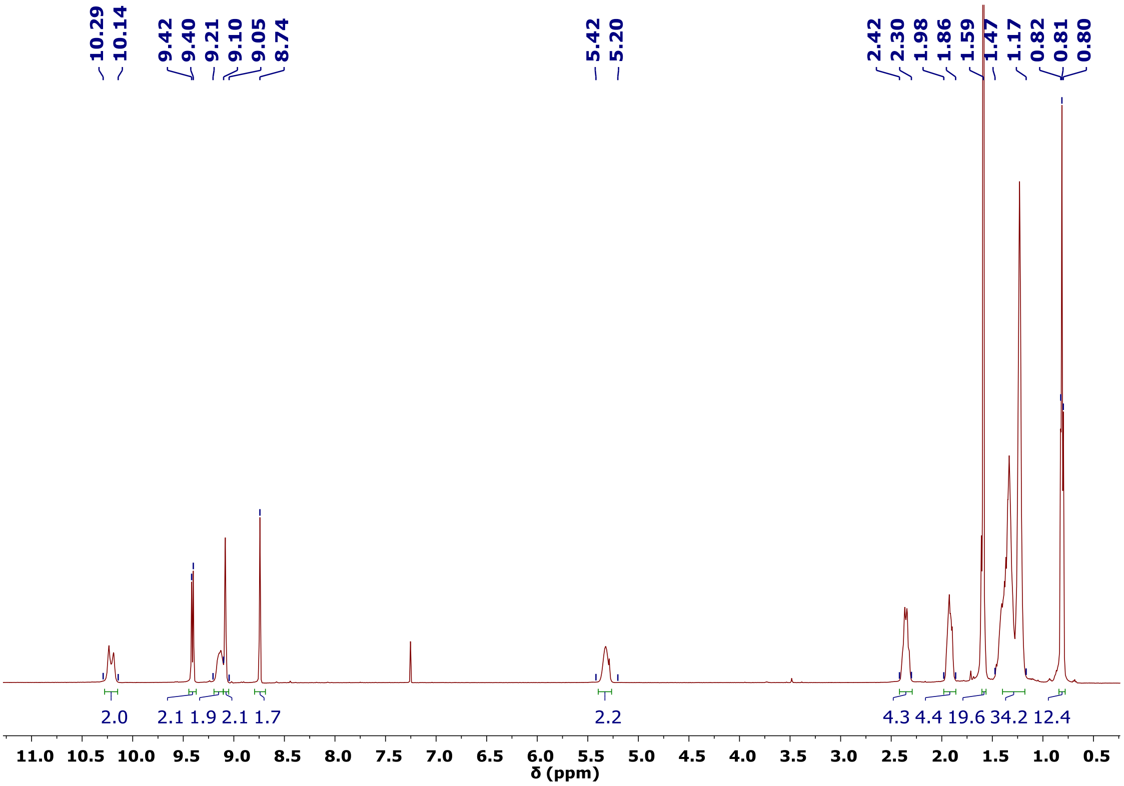

Figure S5. ^{1}H NMR spectrum (500 MHz, $CDCl_3$) of **1**

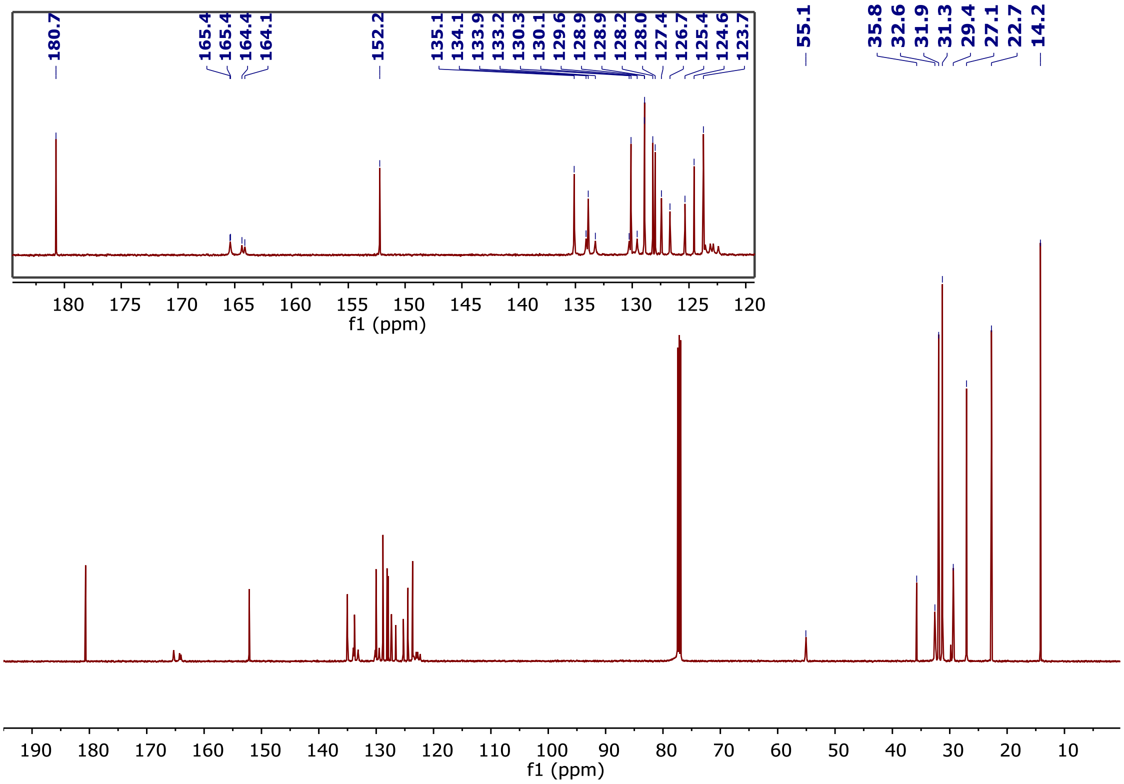

Figure S6. ^{13}C{^{1}H} spectrum (126 MHz, $CDCl_3$) of **1**

4.4. ^{1}H and ^{13}C spectra of 2 in $CDCl_3$

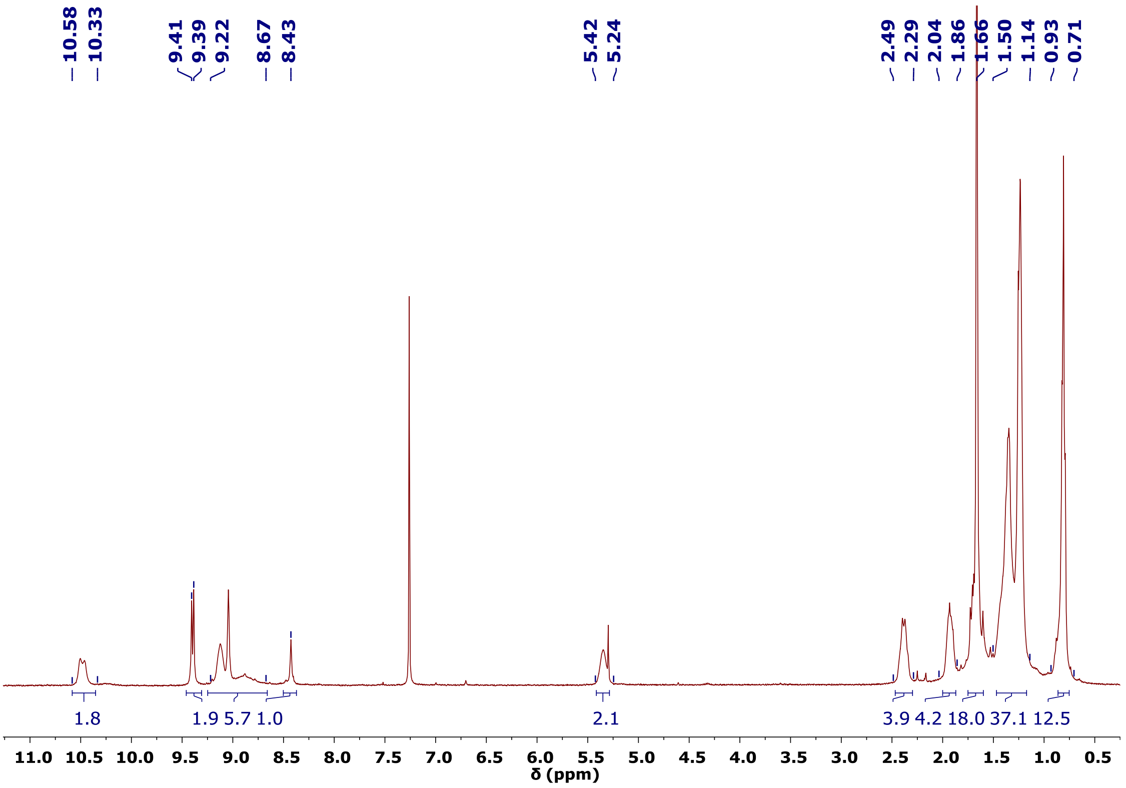

Figure S7. ^{1}H NMR spectrum (400 MHz, $CDCl_3$) of **2**

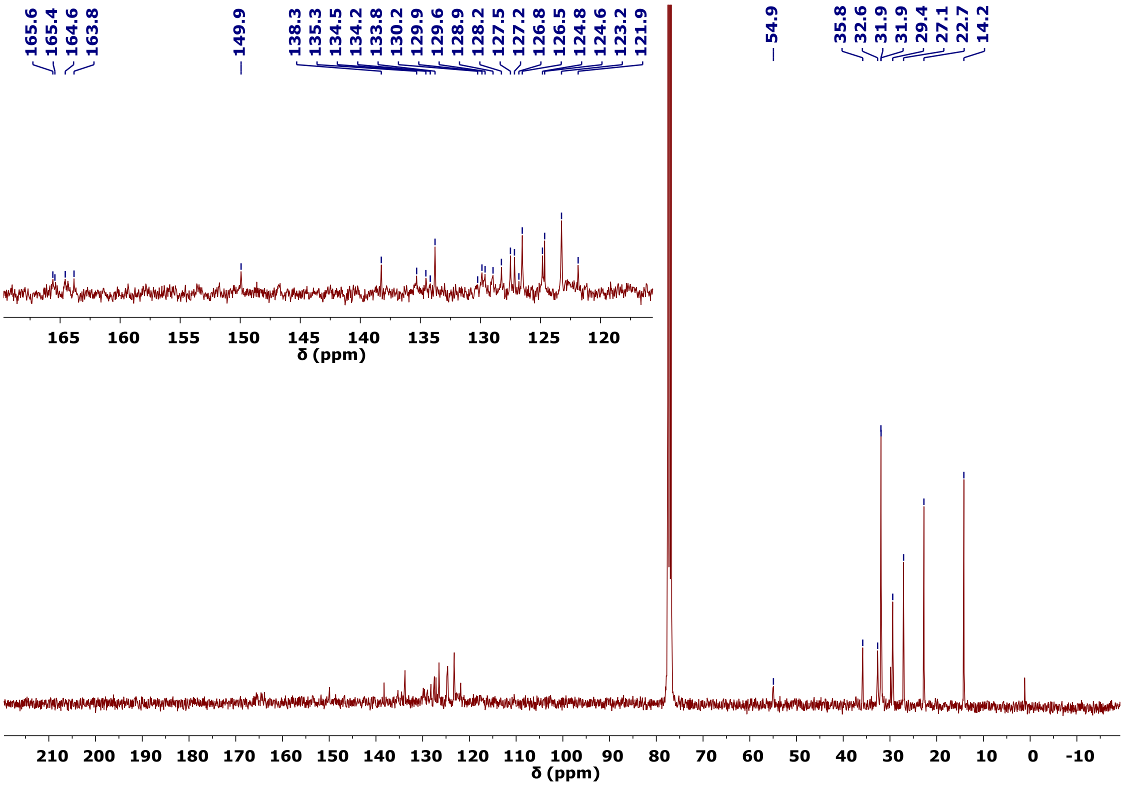

Figure S8. $^{13}C\{^{1}H\}$ spectrum (100 MHz, $CDCl_3$) of **2**

4.5. ^{1}H and ^{13}C spectra of 3 in $CDCl_3$

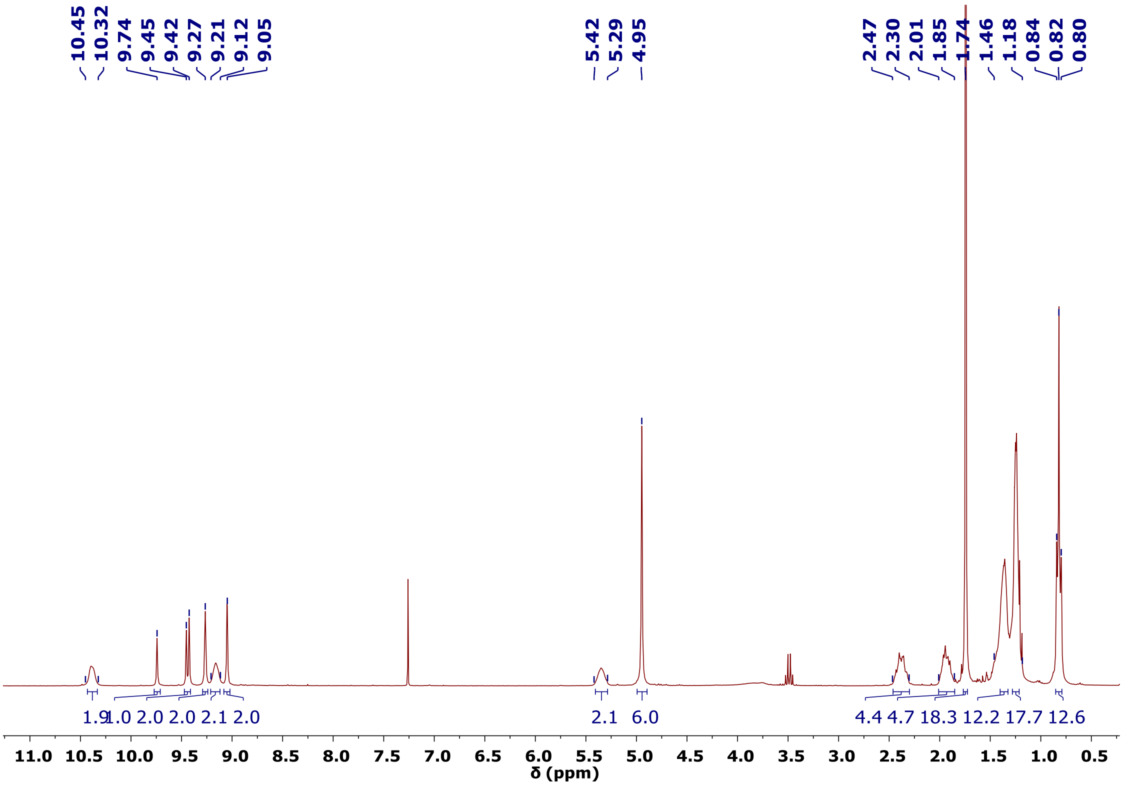

Figure S9. ^{1}H NMR spectrum (300 MHz, $CDCl_3$) of **3**

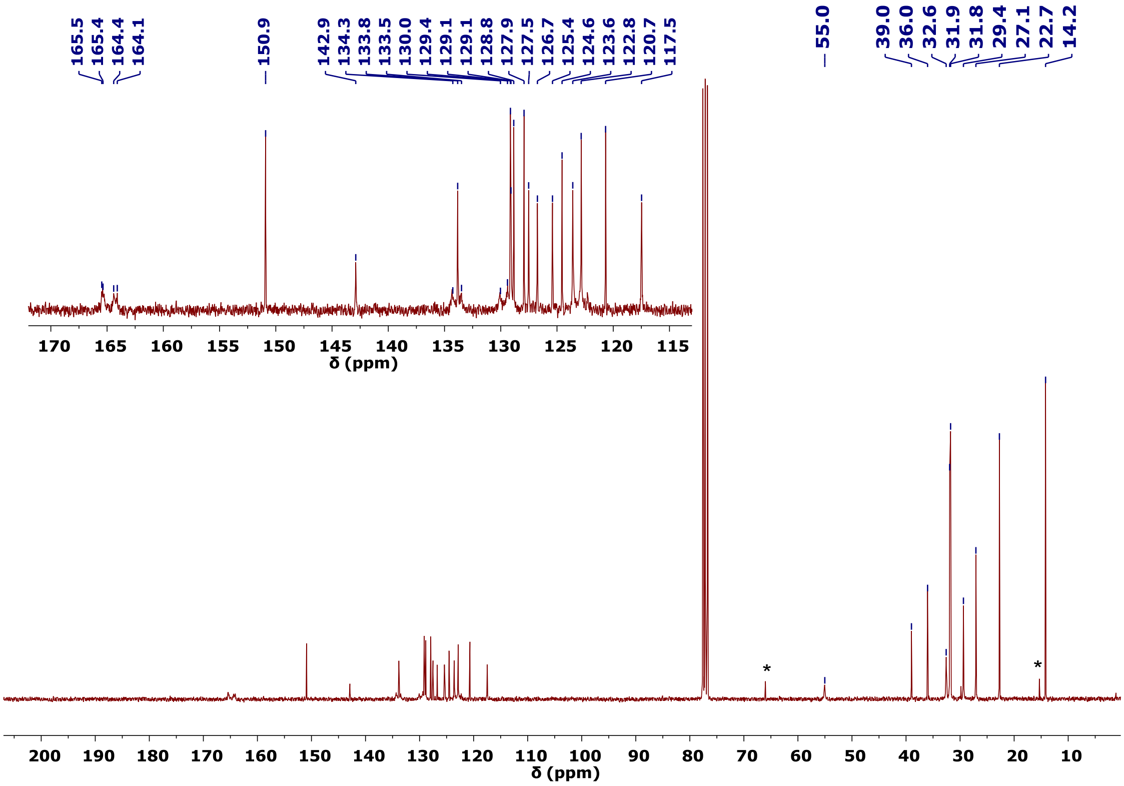

Figure S10. $^{13}C\{^{1}H\}$ spectrum (75 MHz, $CDCl_3$) of **3**

4.6. ^{1}H, ^{13}C, HSQC and HMBC spectra of 4 in $CDCl_3$

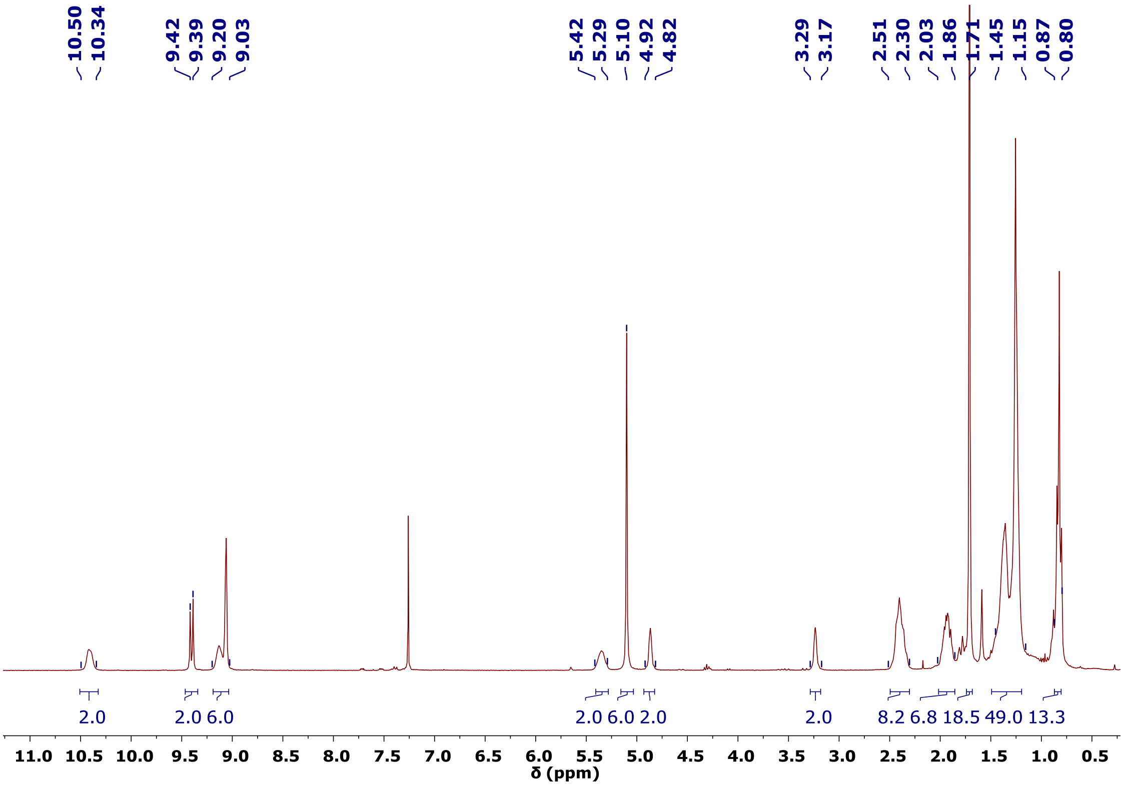

Figure S11. ^{1}H NMR spectrum (300 MHz, $CDCl_3$) of **4**

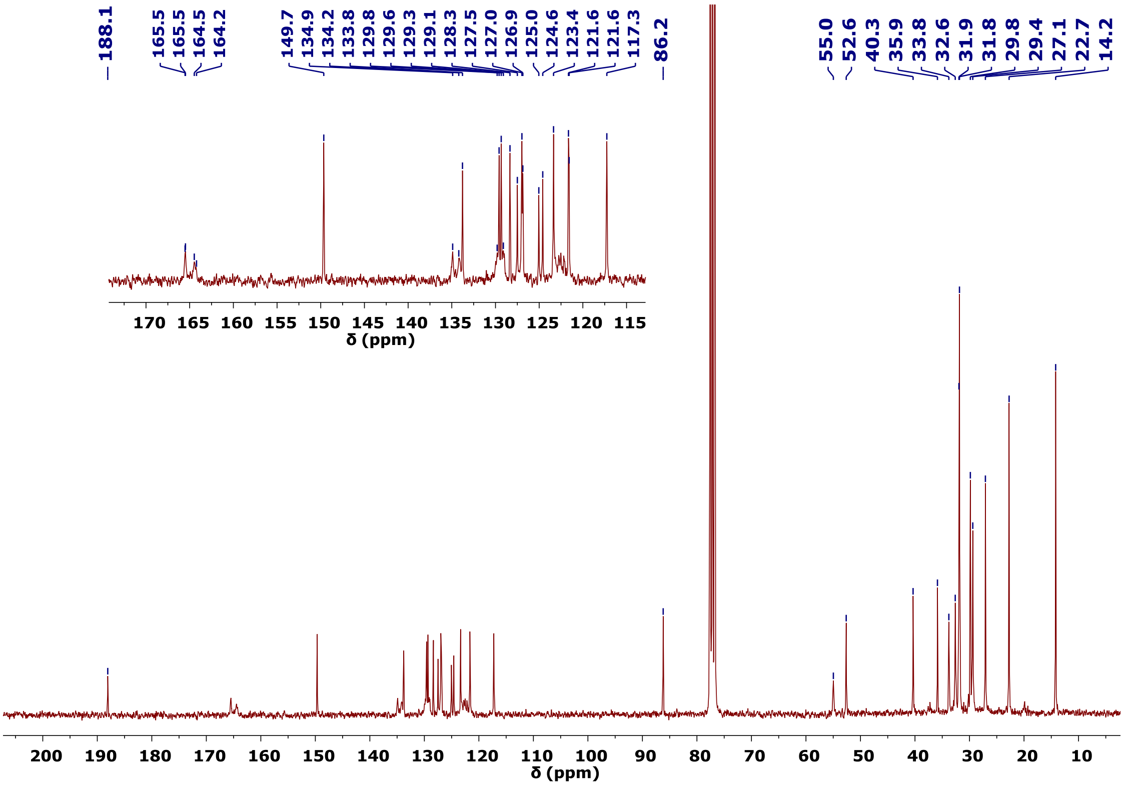

Figure S12. ^{13}C{^{1}H} spectrum (75 MHz, $CDCl_3$) of **4**

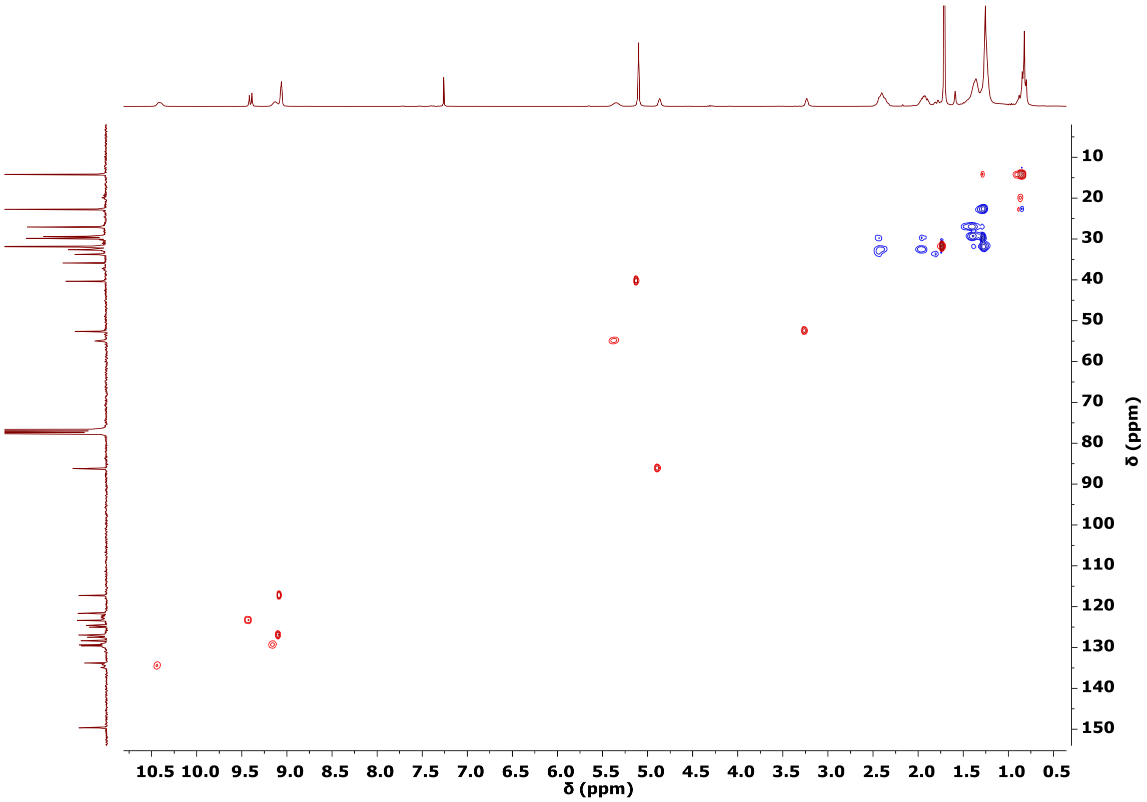

Figure S13. ^{1}H-^{13}C HSQC spectrum (300 MHz, $CDCl_3$) of **4**

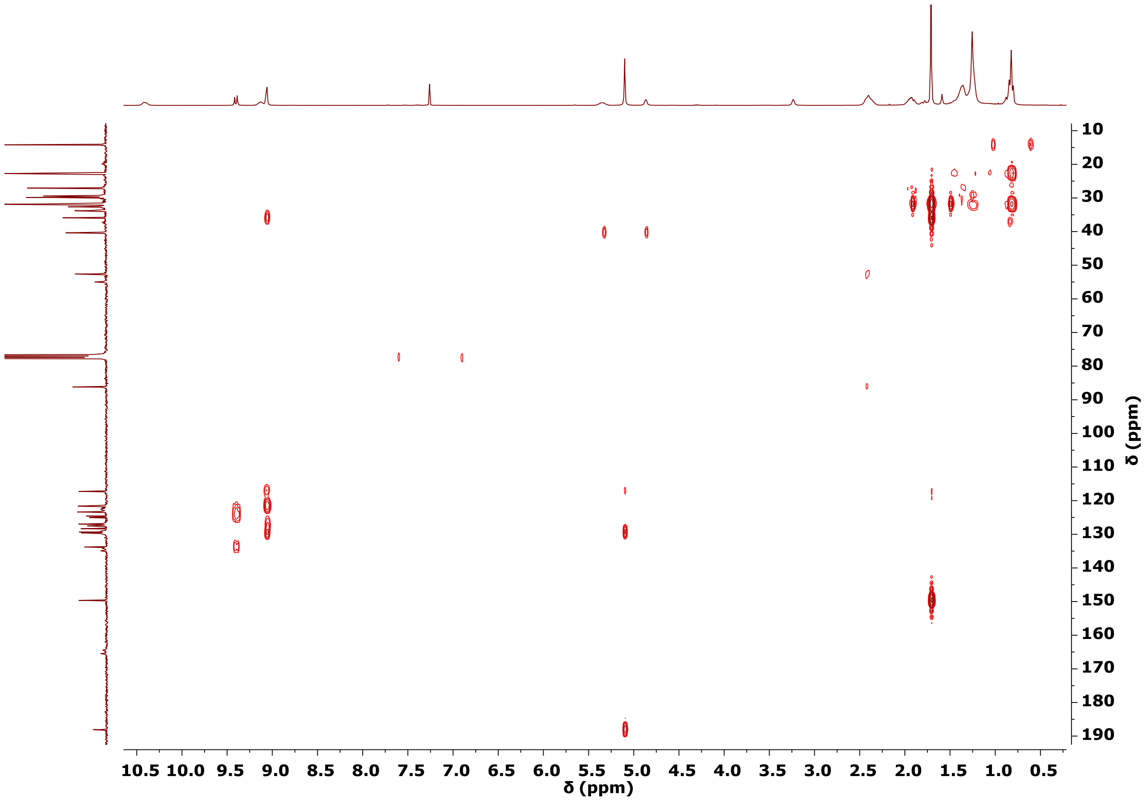

Figure S14. ^{1}H-^{13}C HMBC spectrum (300 MHz, $CDCl_3$) of **4**

4.7. ^{1}H and ^{13}C spectra of 5 in $CDCl_3$

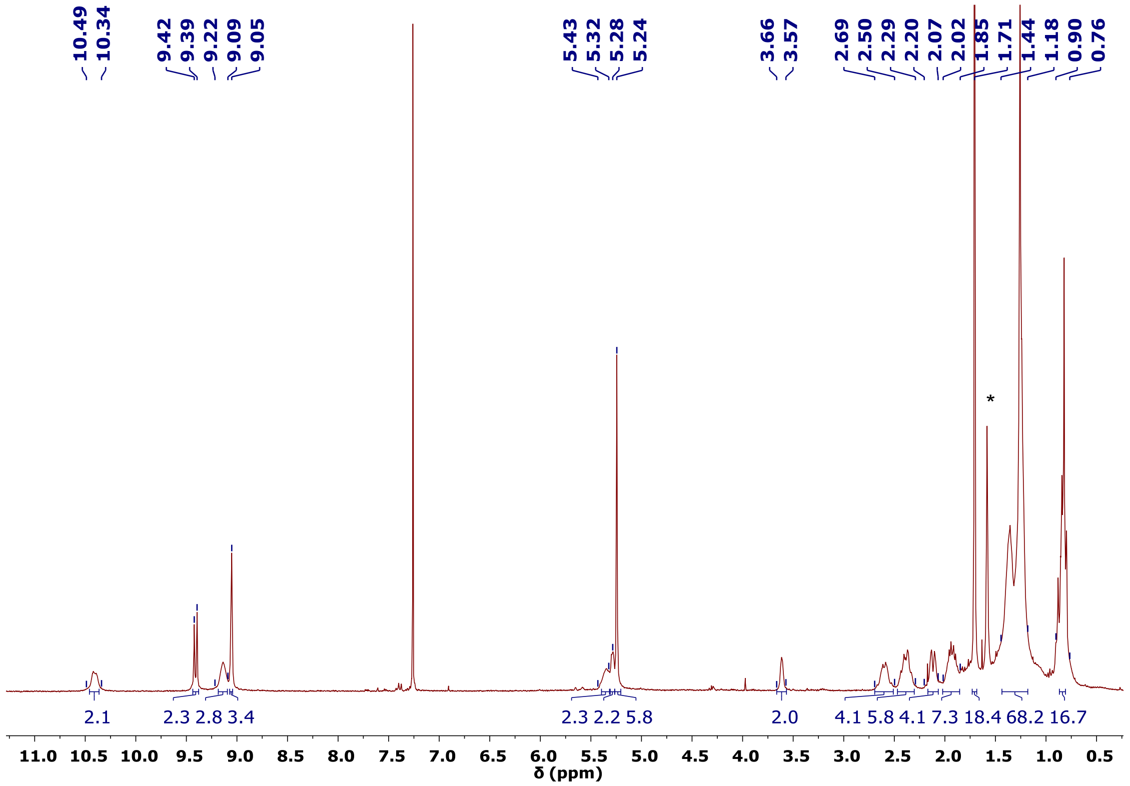

Figure S15. ^{1}H NMR spectrum (300 MHz, $CDCl_3$) of **5**

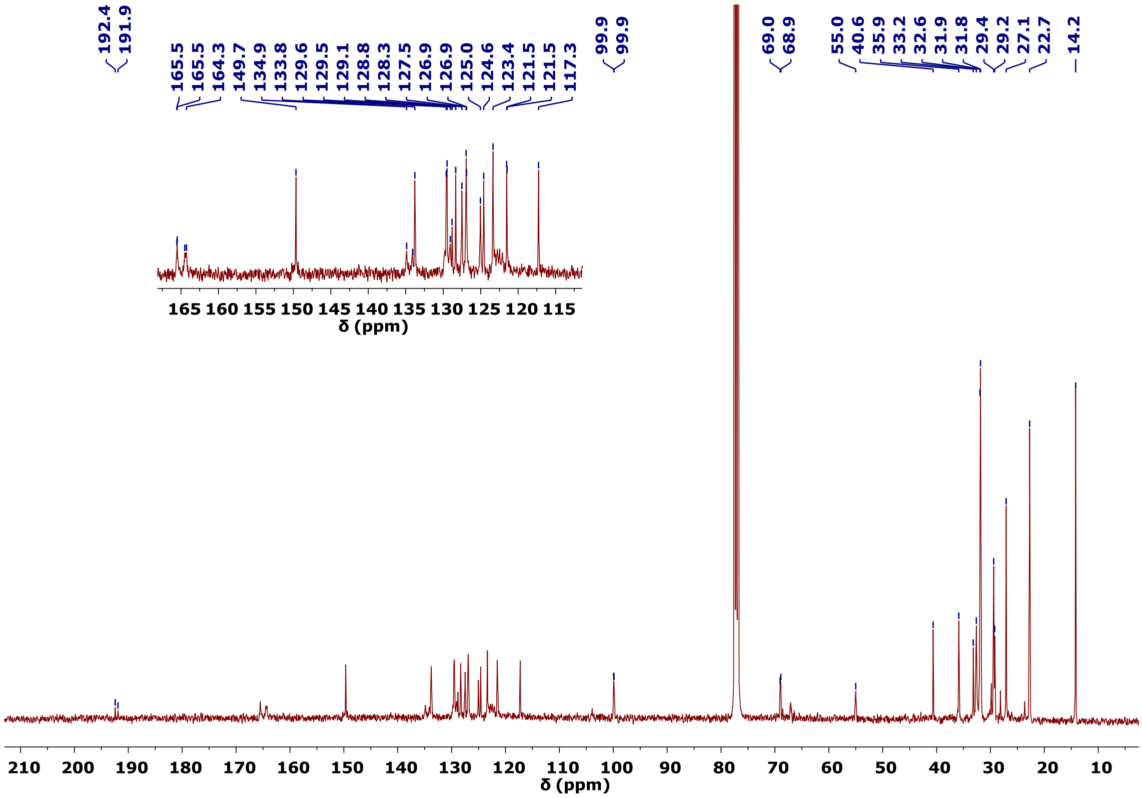

Figure S16. $^{13}C\{^{1}H\}$ spectrum (100 MHz, $CDCl_3$) of **5**

4.8. ^{1}H, ^{13}C and HMBC spectra of 6 in CDCl$_3$

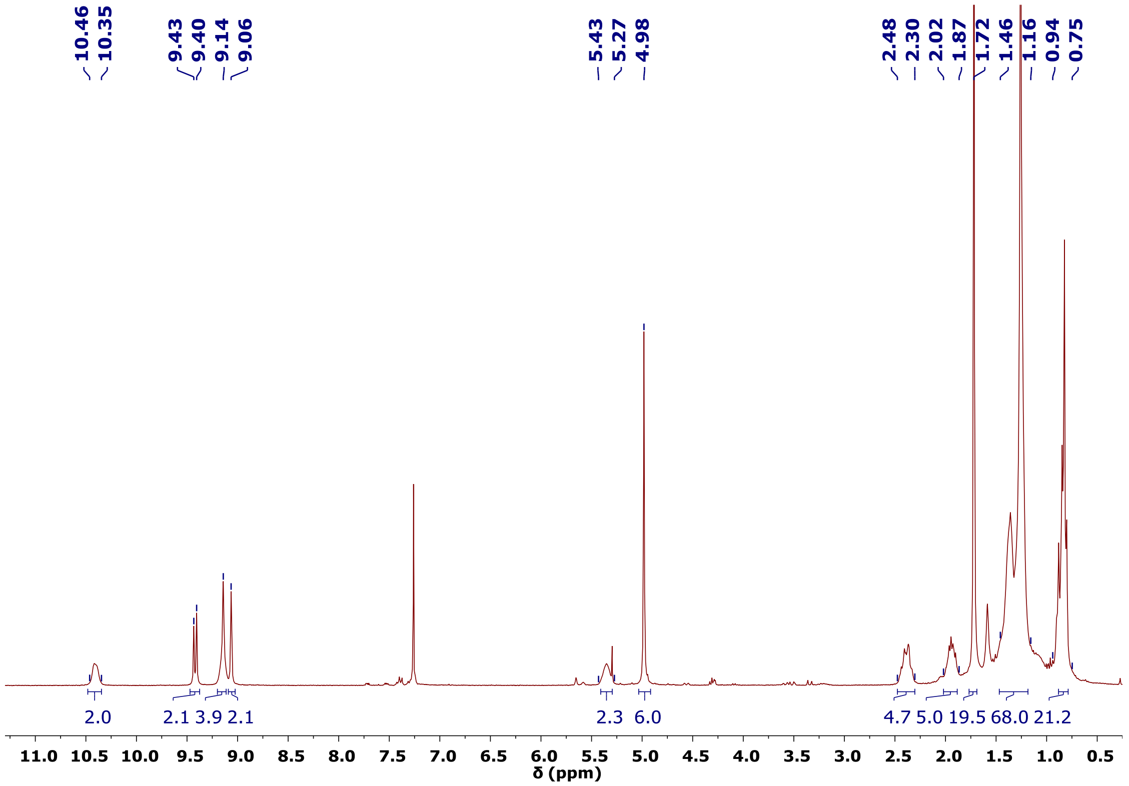

Figure S17. ^{1}H NMR spectrum (300 MHz, CDCl$_3$) of **6**

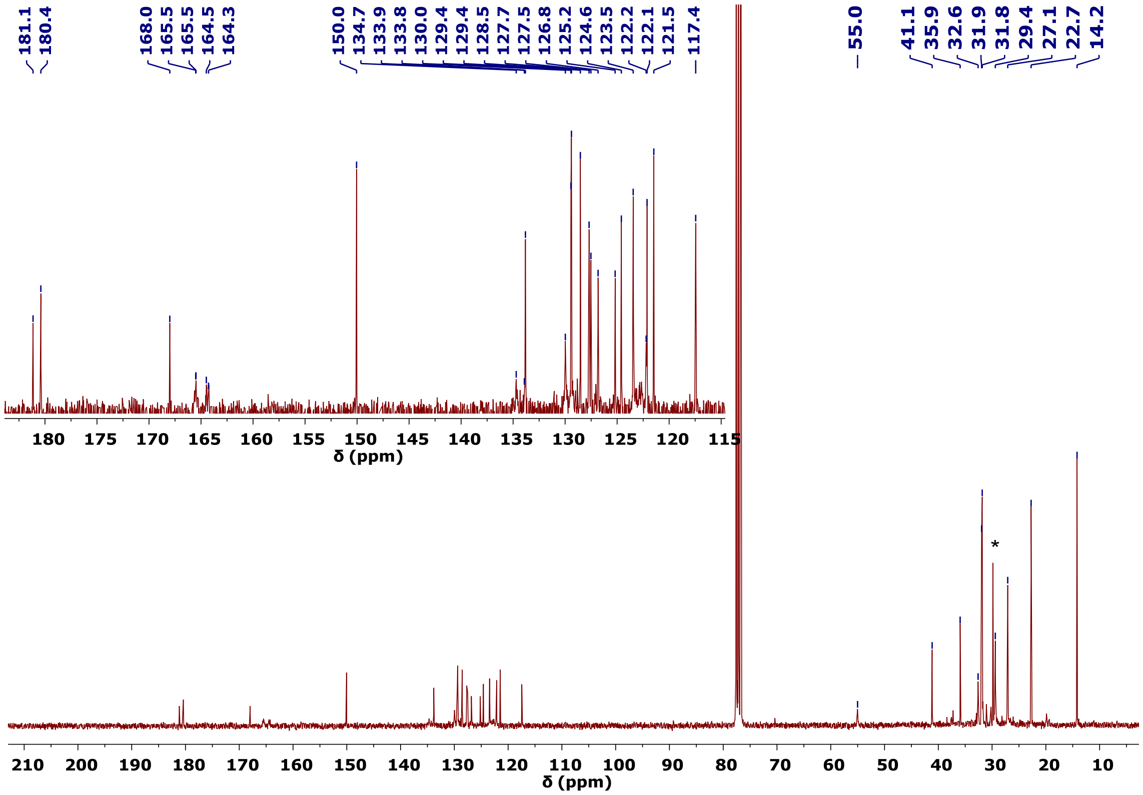

Figure S18. ^{13}C{^{1}H} spectrum (75 MHz, CDCl$_3$) of **6**

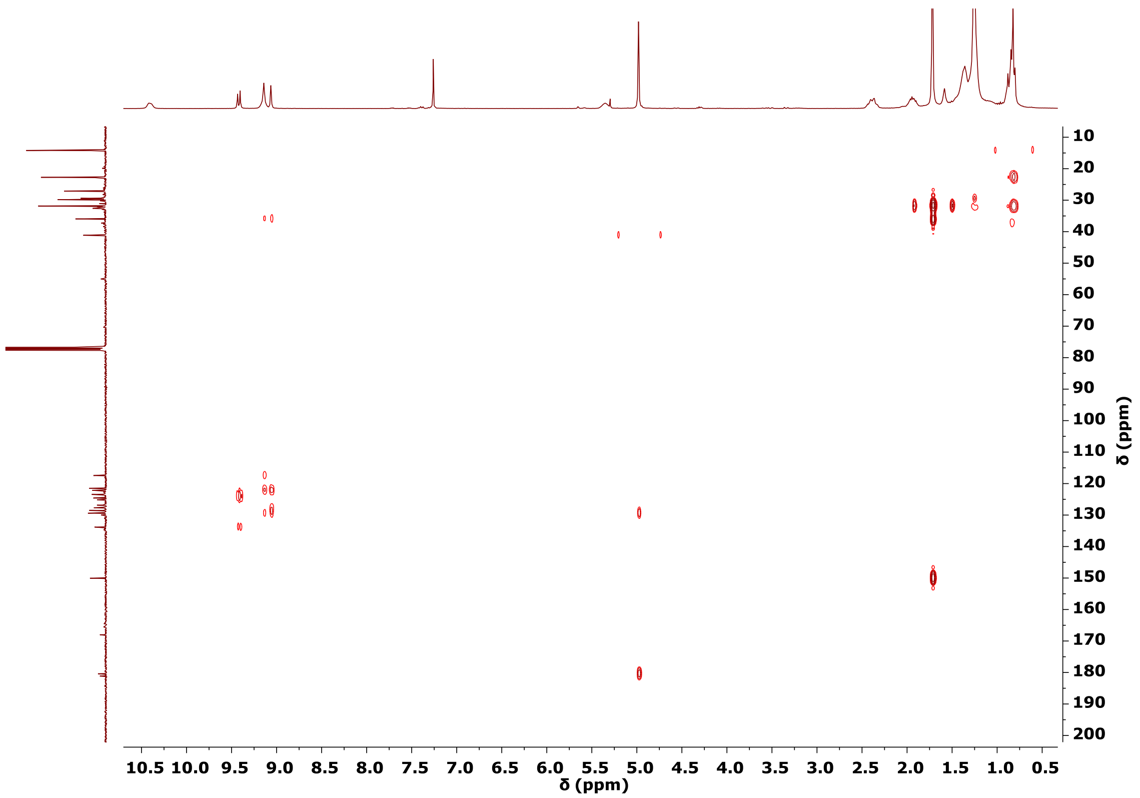

Figure S19. ^{1}H-^{13}C HMBC spectrum (300 MHz, $CDCl_3$) of **6**

4.9. ^{1}H, ^{13}C and HMBC spectra of 7 in $CDCl_3$

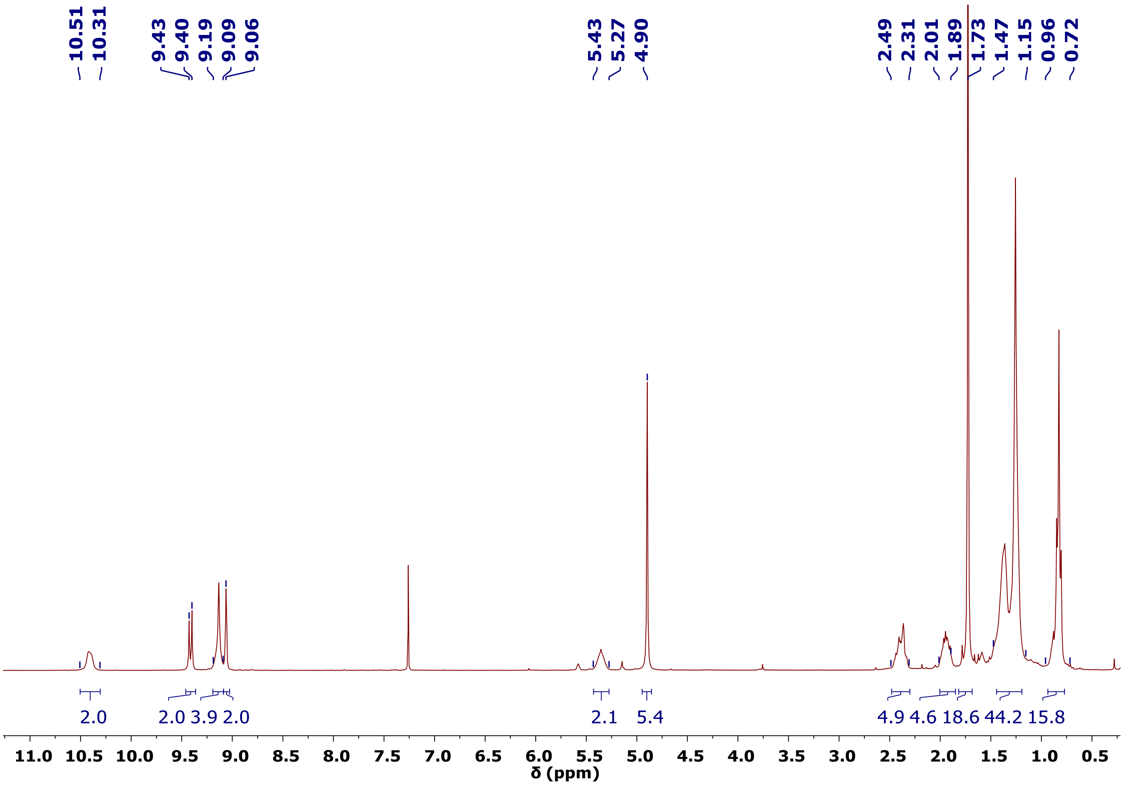

Figure S20. ^{1}H NMR spectrum (300 MHz, $CDCl_3$) of **7**

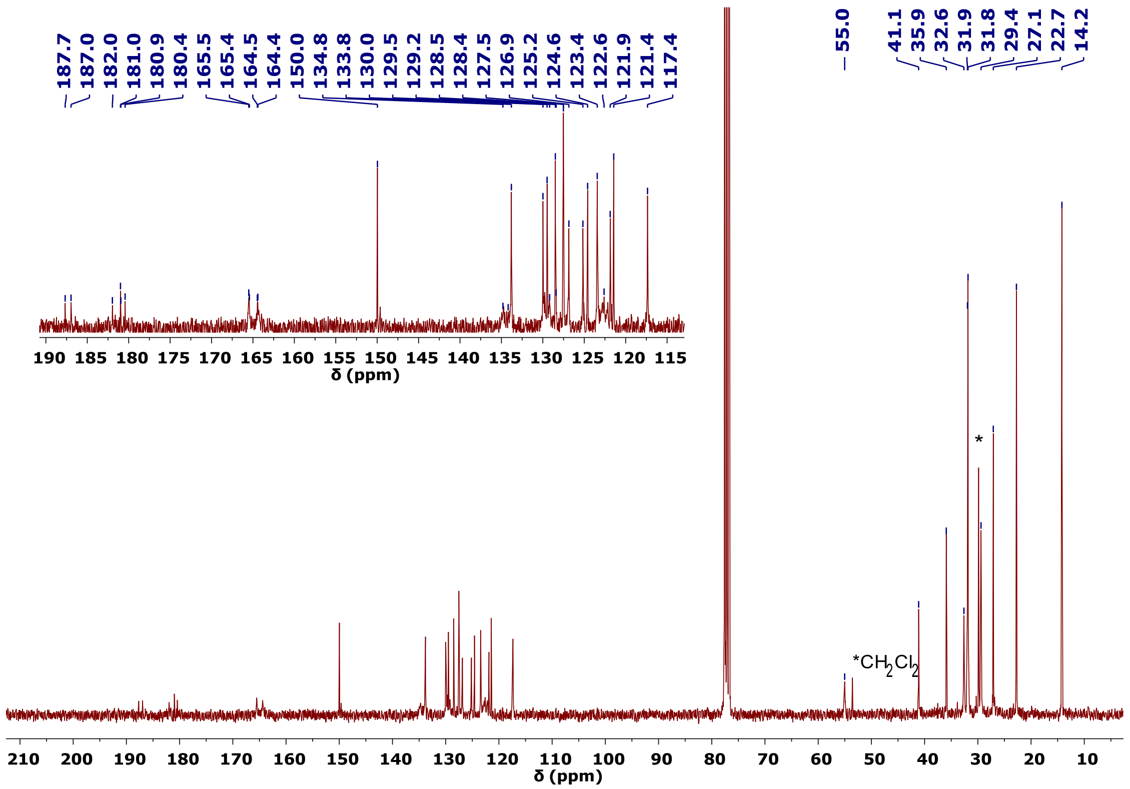

Figure S21. $^{13}C\{^{1}H\}$ spectrum (75 MHz, $CDCl_3$) of **7**

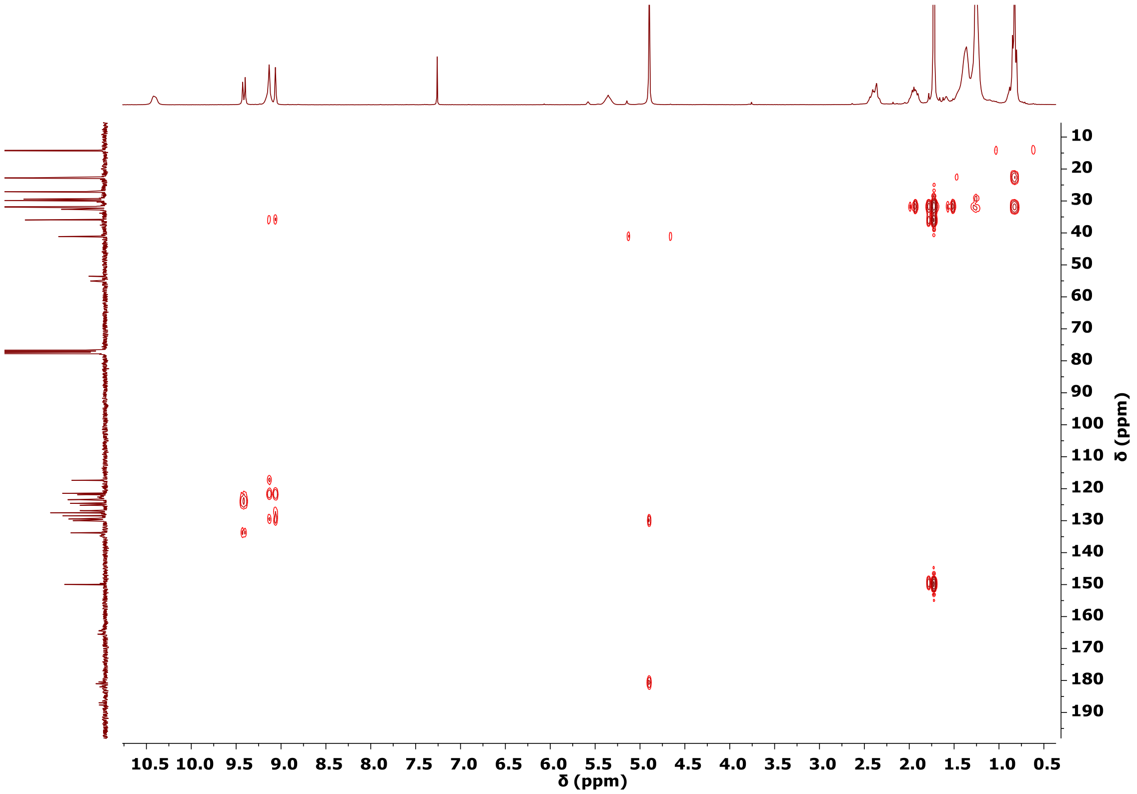

Figure S22. ^{1}H-^{13}C HMBC spectrum (300 MHz, $CDCl_3$) of **7**

5. HR MALDI-TOF mass spectra

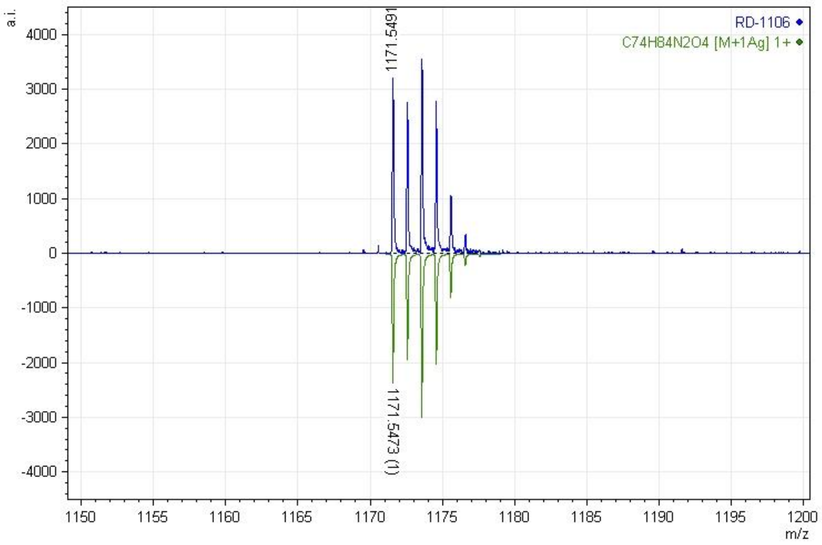

Figure S23. HR-MS of **D** (MALDI-TOF m/z – Reflector mode; Matrix: 10 mg/mL DCTB + 1 mg/mL silver trifluoroacetate).

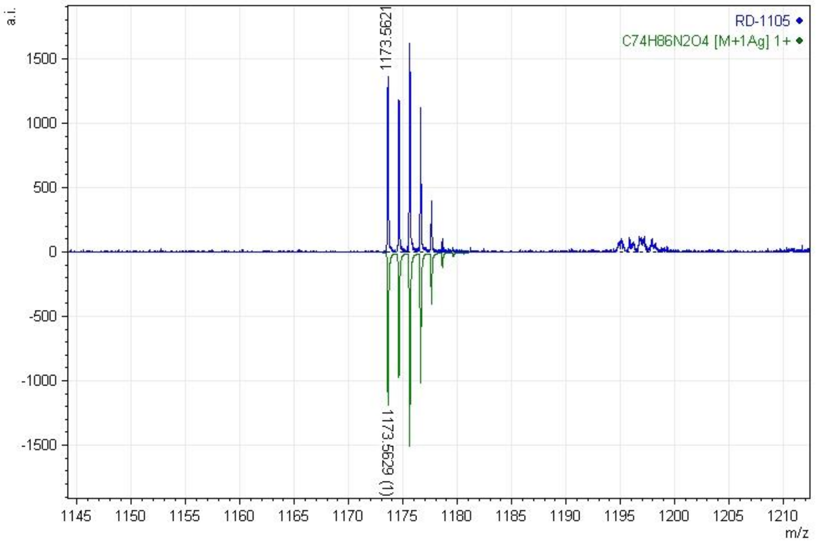

Figure S24. HR-MS of **E** (MALDI-TOF m/z – Reflector mode; Matrix: 10 mg/mL DCTB + 1 mg/mL silver trifluoroacetate).

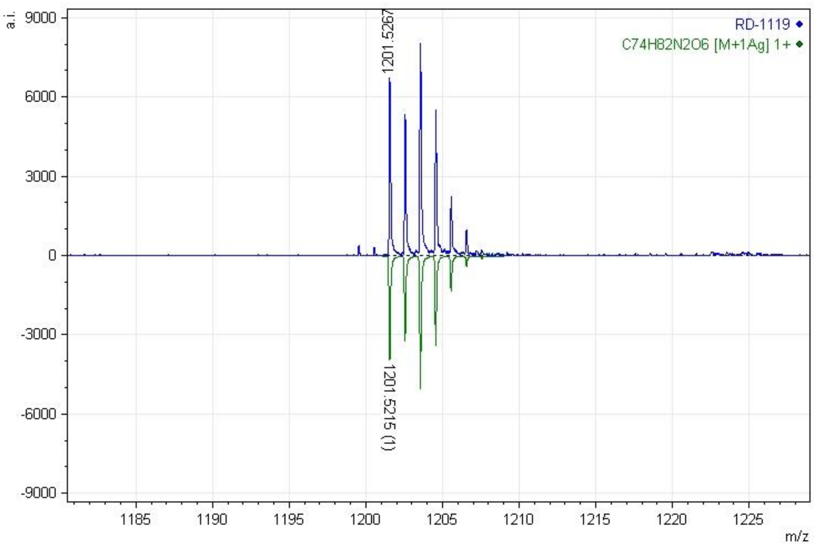

Figure S25. HR-MS of **1** (MALDI-TOF m/z– Reflector mode; Matrix: 10 mg/mL DCTB + 1 mg/mL silver trifluoroacetate).

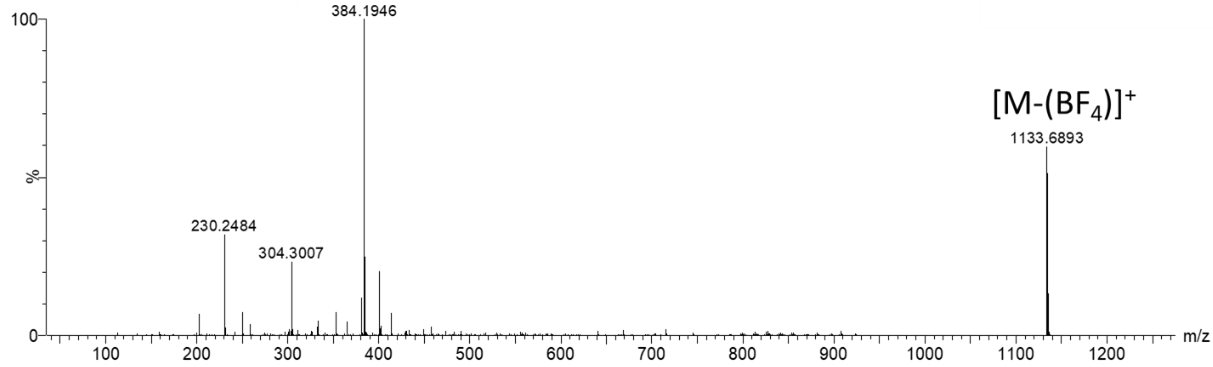

Figure S26. ESI Q-TOF mass spectrum of [**3**](BF_4) in acetonitrile.

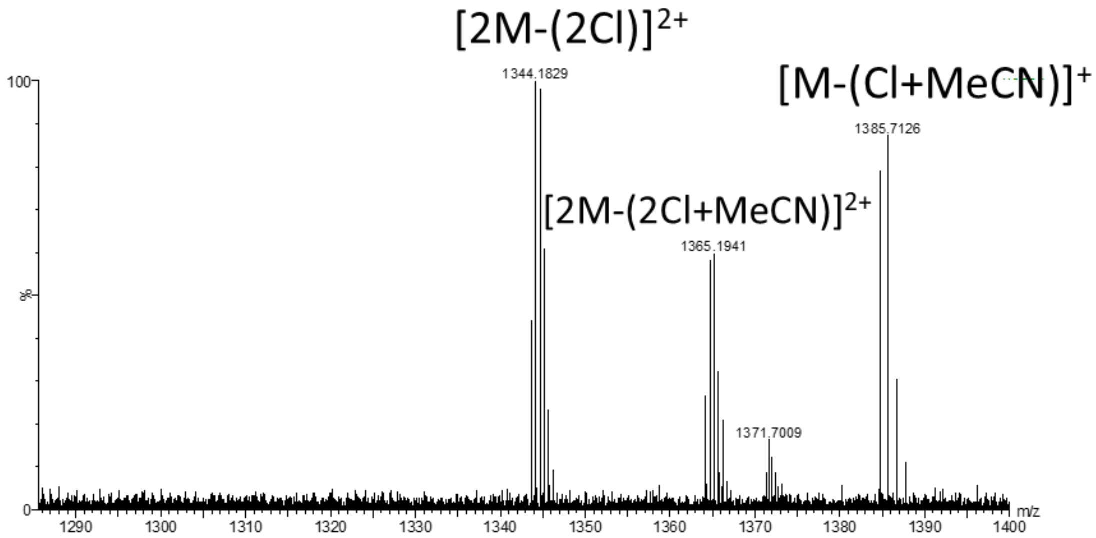

Figure S27. ESI Q-TOF mass spectrum of **5** in acetonitrile.

6. Electrochemical studies

6.1. Electrochemical measurements

Electrochemical studies were carried out by using an Autolab Potentiostat, Model PGSTAT101 controlled with NOVA 2.1.4 software. In all experiments, $[N(nBu)_4][PF_6]$ (0.1 M in dry and deoxygenated CH_2Cl_2) was used as the supporting electrolyte with an analyte concentration of 1 mM. Cyclic voltammetry was performed in a cell, under Ar atmosphere and with disk glassy carbon working electrode, platinum counter electrode, and a silver wire pseudoreference electrode. Measurements were performed at 100 mV s^{-1} scan rate. All scans were referenced to the ferrocenium/ferrocene (Fc^+/Fc) couple at 0 V. Ohmic drop was minimized by minimizing the distance between the working and reference electrodes. The residual ohmic drop was estimated by positive feedback and compensated at 95%.

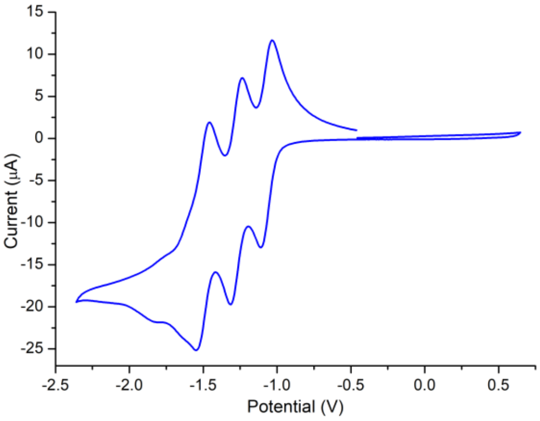

Figure S28. Cyclic voltammogram of **1**

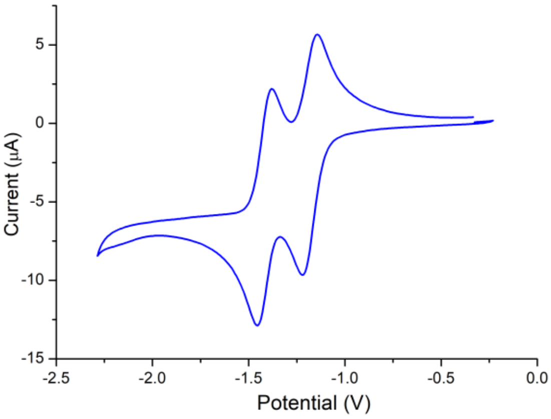

Figure S29. Cyclic voltammogram of [**3**](BF_4)

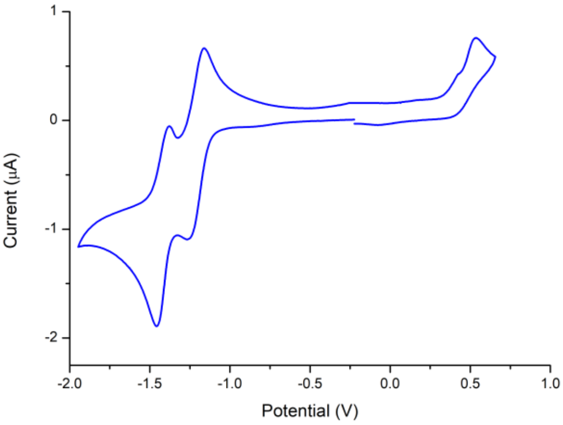

Figure S30. Cyclic voltammogram of **4**

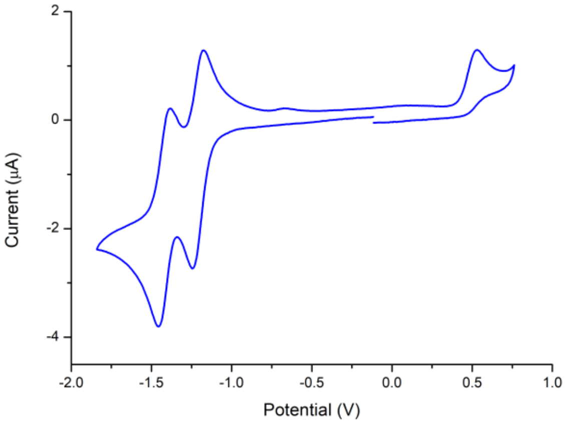

Figure S31. Cyclic voltammogram of **5**

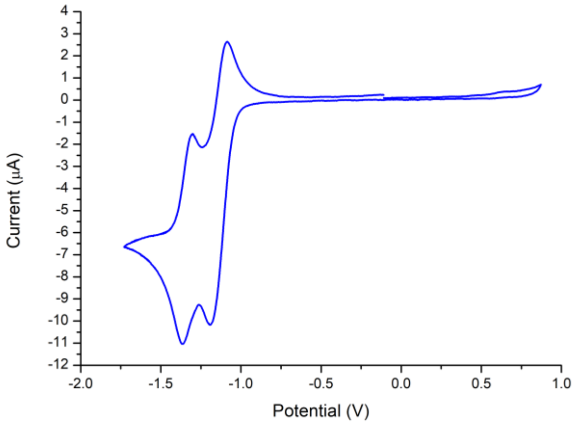

Figure S32. Cyclic voltammogram of **6**

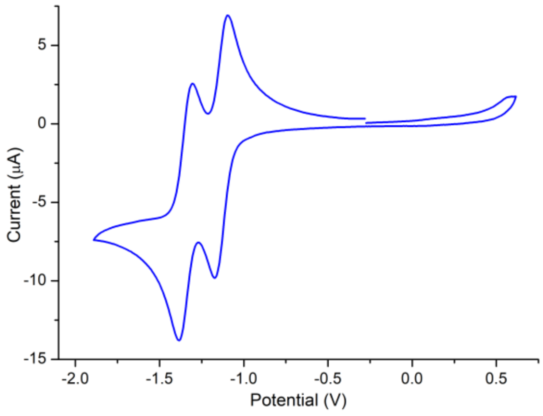

Figure S33. Cyclic voltammogram of **7**

Table S1. Electrochemical properties of compounds **1**, [**3**](BF_4), **4**, **5**, **6** and **7**.[a]

Compound	$E^4_{1/2}$ (V)/ΔE	$E^3_{1/2}$ (V)/ΔE (mV)	$E^2_{1/2}$ (V)/ΔE (mV)	$E^1_{1/2}$ (V)/ΔE (mV)	E_p^a (V)
**1**	-1.81/87	-1.50/95	-1.27/79	-1.07/71	-
[**3**](BF_4)			-1.42/79	-1.18/79	-
**4**			-1.42/78	-1.21/102	0.54
**5**			-1.42/69	-1.21/72	0.53
**6**			-1.33/61	-1.14/106	-
**7**			-1.34/79	-1.13/71	-

[a]Cyclic voltammograms performed in dry CH_2Cl_2 with 1 mM analyte and 0.1 M [N(*n*Bu)$_4$][PF_6]. Measurements performed at 100 mVs^{-1} and referenced *vs* ferrocenium/ferrocene.

6.2. Spectroelectrochemical measurements

The SEC cell contained a masked Au-minigrid working electrode (32 wires/cm), a Pt-gauze auxiliary electrode, and an Ag-wire pseudo-reference electrode and had CaF_2 windows. In each experiment, electrochemical Spectroelectrochemical (SEC) measurements were performed using a gastight, optically transparent thin-layer solution cell fabricated by Prof. Hartl at the University of Reading (Reading, U.K.), as described previously.[5] reduction of the species of interest ([Analyte] = 3 mM for IR experiments and 0.25 mM for UV-vis experiments, [N(*n*Bu)$_4$][PF_6] = 100 mM in CH_2Cl_2 under inert atmosphere) was monitored by the appropriate spectroscopy for a period of 2−5 min. First, the potential of the cell was swept negatively starting at the open circuit potential,

recording a thin-layer cyclic voltammogram (5 mV/s) to identify the potential window of interest. Then, fresh analyte solution was introduced in the cell and the potential was varied within range of interest in 50 mV steps. The electrolysis step did not exceed 30 s. After each step, an IR or UV-vis spectrum was collected. Diffusion and mixing of the redox products generated at the working and auxiliary electrodes in the cell were reasonably suppressed within the total experimental time (no more than 5 min for one complete measurement).

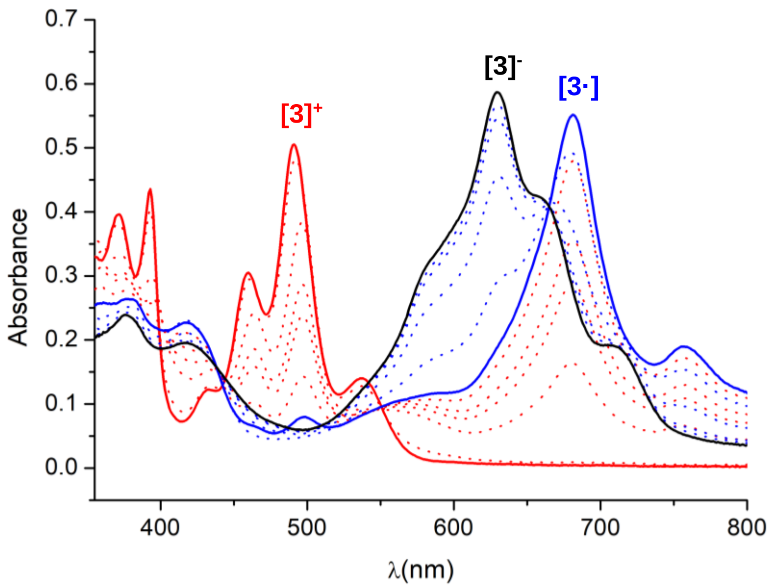

Figure S34. UV-vis SEC monitoring reduction of **3** in CH_2Cl_2 (0.1 M [N(*n*Bu)$_4$][PF$_6$]). The solid lines represent the spectra of the starting (red), singly-reduced (blue) and doubly-reduced (black) species.

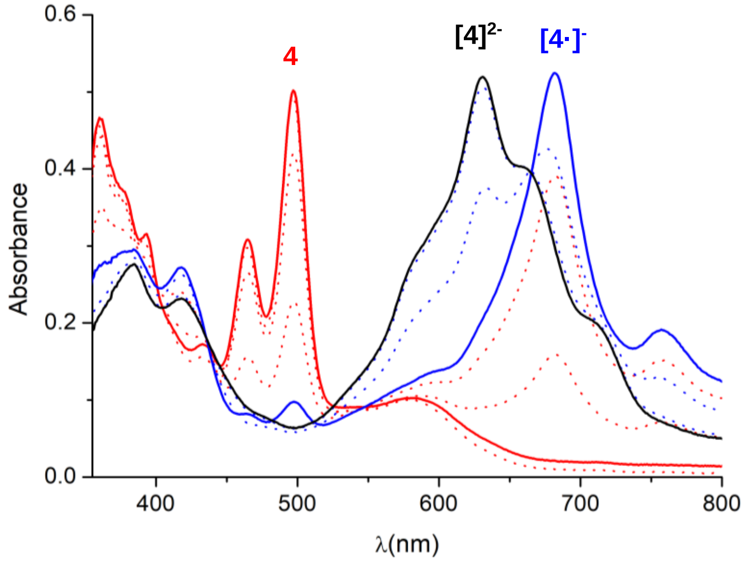

Figure S35. UV-vis SEC monitoring reduction of **4** in CH_2Cl_2 (0.1 M [N(*n*Bu)$_4$][PF_6]). The solid lines represent the spectra of the starting (red), singly-reduced (blue) and doubly-reduced (black) species

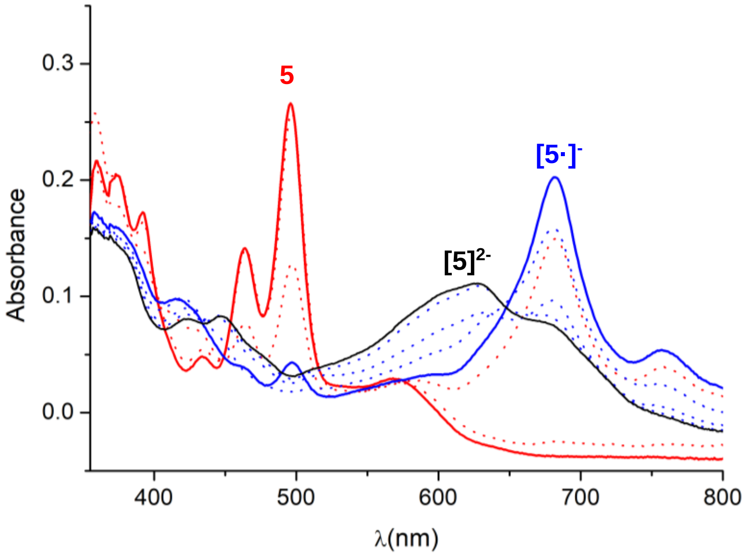

Figure S36. UV-vis SEC monitoring reduction of **5** in CH_2Cl_2 (0.1 M [N(*n*Bu)$_4$][PF_6]). The solid lines represent the spectra of the starting (red), singly-reduced (blue) and doubly-reduced (black) species

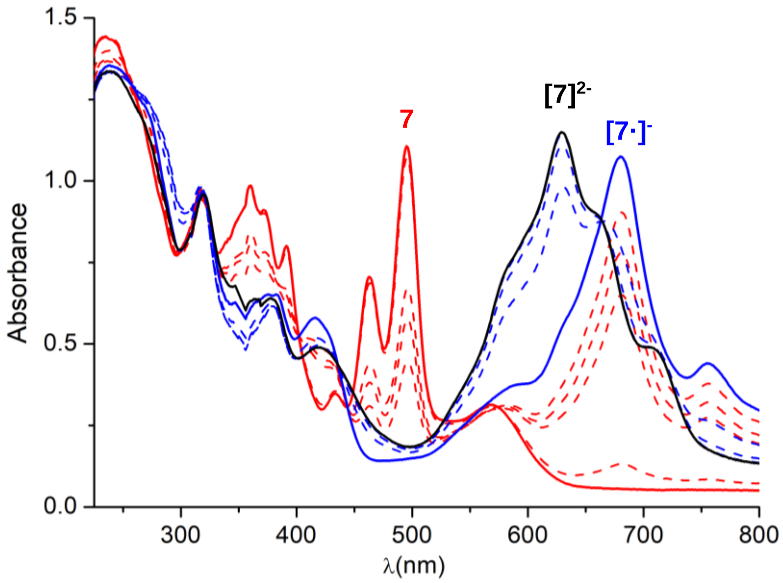

Figure S37. UV-vis SEC monitoring reduction of **7** in CH_2Cl_2 (0.1 M [N(*n*Bu)$_4$][PF_6]). The solid lines represent the spectra of the starting (red), singly-reduced (blue) and doubly-reduced (black) species

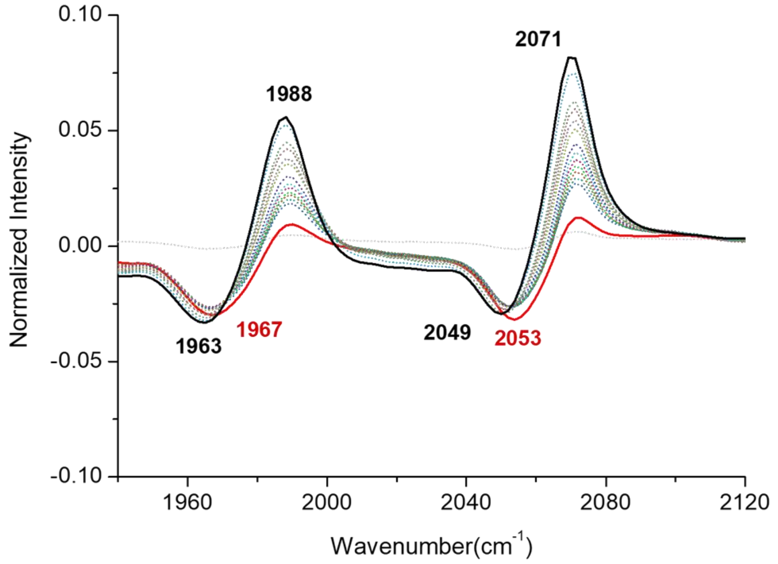

Figure S38. Infrared difference spectra resulting from the IR-SEC reduction of **6** in dry CH_2Cl_2 (0.1 M [N(*n*Bu)$_4$][PF$_6$]).

7. Photophysical properties

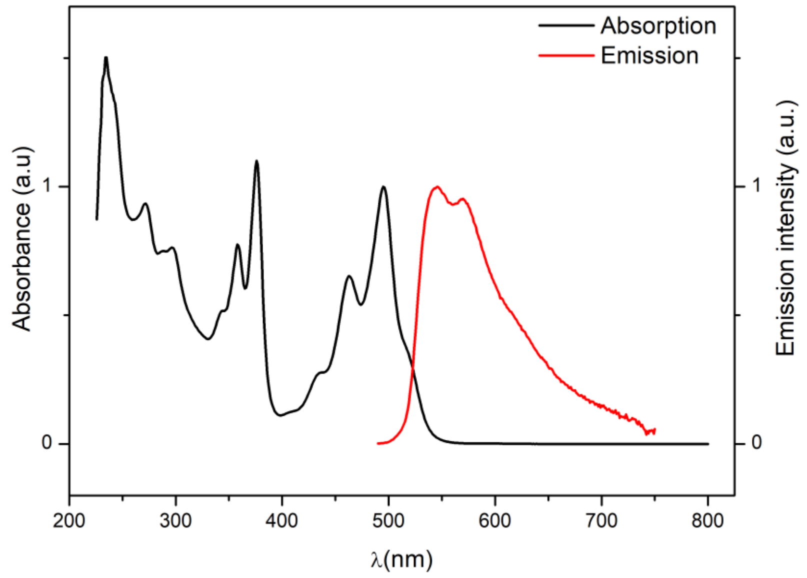

Figure S39. UV-Vis and emission spectra excited at 470 nm of **1**, recorded in CH_2Cl_2 at a concentration of 50 µM. Molar extinction coefficients (ε, in M^{-1} cm^{-1}) were determined from Beer's law plots: log (ε) at 463 nm = 4.57

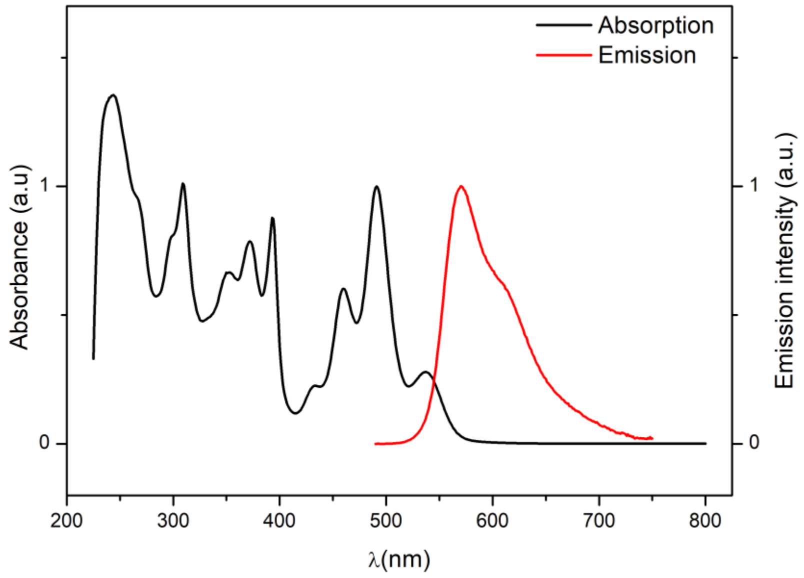

Figure S40. UV-Vis and emission spectra excited at 470 nm of [**3**](BF_4), recorded in CH_2Cl_2 at a concentration of 50 µM. Molar extinction coefficients (ε, in M^{-1} cm^{-1}) were determined from Beer's law plots: log (ε) at 459 nm = 4.47

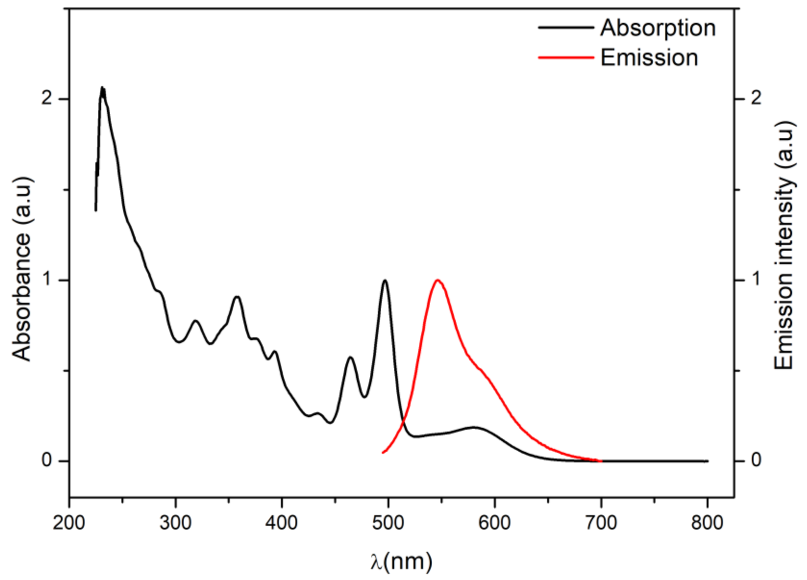

Figure S41. UV-Vis and emission spectra excited at 470 nm of **4**, recorded in CH_2Cl_2 at a concentration of 50 µM. Molar extinction coefficients (ε, in M^{-1} cm^{-1}) were determined from Beer's law plots: log (ε) at 466 nm = 4.37

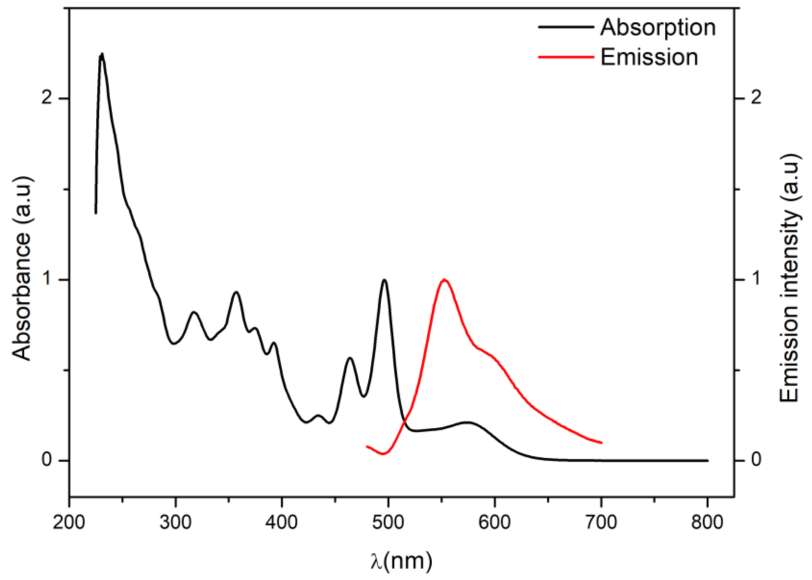

Figure S42. UV-Vis and emission spectra excited at 470 nm of **5**, recorded in CH_2Cl_2 at a concentration of 50 µM. Molar extinction coefficients (ε, in M^{-1} cm^{-1}) were determined from Beer's law plots: log (ε) at 463 nm = 4.31

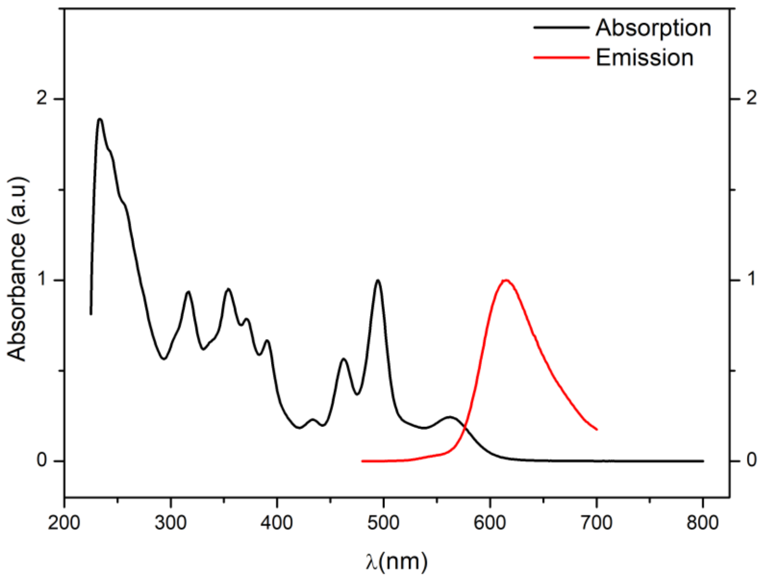

Figure S43. UV-Vis and emission spectra excited at 470 nm of **6**, recorded in CH_2Cl_2 at a concentration of 50 µM. Molar extinction coefficients (ε, in M^{-1} cm^{-1}) were determined from Beer's law plots: log (ε) at 465 nm = 4.29

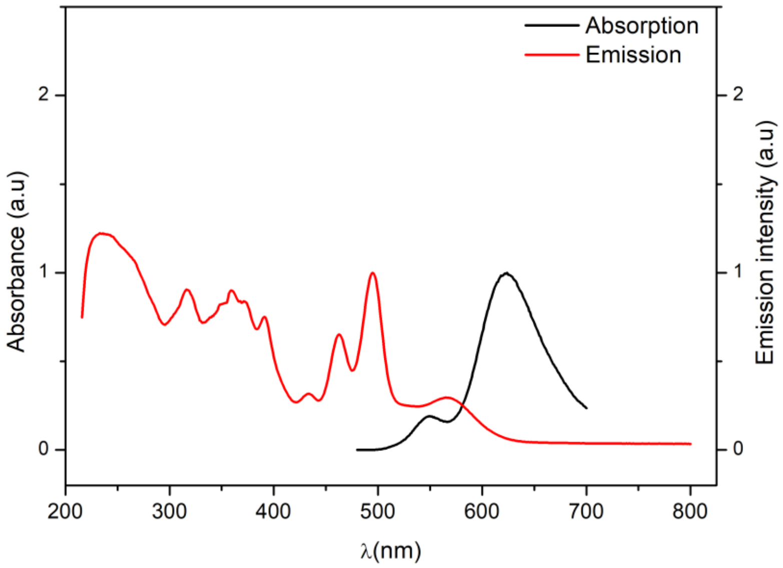

Figure S44. UV-Vis and emission spectra excited at 470 nm of **7**, recorded in CH_2Cl_2 at a concentration of 50 µM. Molar extinction coefficients (ε, in M^{-1} cm^{-1}) were determined from Beer's law plots: log (ε) at 463 nm = 4.27

Table S2. Photophysical properties of compounds

Compound	λ_{abs} [nm][a]	$\lambda_{em, max}$ [nm][a]	ϕ_f[b]
**1**	359, 377, 463, 496	546, 570	0.02
**3**	353, 373, 393, 459, 491, 538	570	0.54
**4**	359, 375, 394, 466, 497,582	550	0.02
**5**	359, 375, 393, 463, 496, 581	549	0.05
**6**	355, 372, 391,465, 495, 564	615	0.28

[a]Measurements were performed in dry and degassed dichloromethane under ambient conditions. [b]Emission quantum yields were measured using a Hamamatsu integration sphere irradiating at 470 nm.

Table S3. Optical and electronic properties of the compounds **3-6** in solution

Compound	$E_{g\ (opt)}$ (eV)[a]	E_{LUMO} (eV)[b]	E_{HOMO} (eV)[c]
**3**	2.30	-3.52	-5.82
**4**	2.13	-3.59	-5.72
**5**	2.13	-3.59	-5.72
**6**	2.20	-3.66	-5.86

[a]Optical band gap calculated using equation $E_g = hc/\lambda_{ae} \approx 1240/\lambda_{ae}$ (nm); Where λ_{ae} denotes the absorption edge wavelength in nm, obtained from offset wavelength derived from the lowest energy absorption band. [b]The LUMO levels were estimated from the onset of the first reduction potential $E_{LUMO} = -(E_{1red(ONSET)} + 4.8eV)$. [c]Estimated from $E_{HOMO} = E_{LUMO} - E_g$.

8. Catalytic studies

8.1. Cycloaddition of diphenylcyclopropenone with methylphenylacetylene

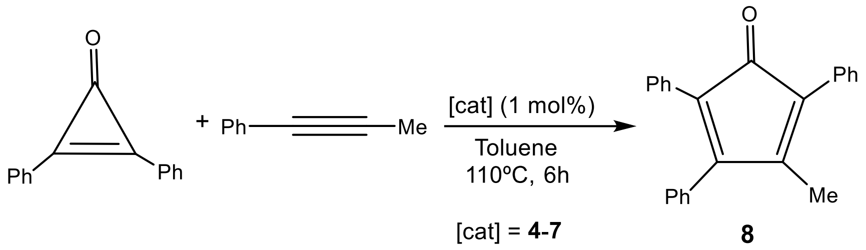

Scheme S3. Cycloaddition of diphenylcyclopropenone with methylphenylacetylene

Cycloaddition reactions of diphenylcyclopropenone with methylphenylacetylene. A thick-walled Schlenk tube fitted with a Teflon cap containing a stirring bar was charged with diphenylcyclopropenone (0.27 mmol, 1.5 eq.), 1,3,5-trimethoxybenzene as internal reference (0.18 mmol, 1 eq.) and the corresponding catalyst **5-7** (0.0018 mmol, 1 mol %; 0.0045 mmol, 2.5 mol % or 0.009 mmol, 5 mol %). Cobaltocene (1.5 eq. respect to the catalyst) was added to the mixture in the indicated reactions. The mixture was deaerated using vacuum and filled with nitrogen three times. After this, methylphenylacetylene (0.18 mmol, 1 eq.), and 0.6 mL of toluene-d_8 were added to the Schlenk under nitrogen. The reaction mixture was stirred at 110ºC until the maximum yield was reached. Yields were determined by ^{1}H-NMR spectroscopy using 1,3,5-trymethoxybenzene as internal standard. The time-dependent reaction profile is plotted in the Figure S45. In addition, Figure S46 shows the rate constant calculated by plotting the ln[methylphenylacetylene] vs. time assuming first order reaction, using catalyst **5**.

The reaction order with respect to the catalyst was determined by plotting ln[rate constant] vs. ln[**5**], where the slope of the straight line indicates that the reaction is half-order with respect to the catalyst (Figure S47). The reaction order with respect to the catalyst was also determined by plotting the concentration of the product against the normalized time scale t[cat]^n (being n the order of the catalyst), according to the method developed by Dr. Burés.[6] Visual analysis of the reaction profiles depicted in Figure S48 indicated half-order in **5** (Figure S48b).

Table S4. Catalytic [3+2] cycloaddition of diphenylcyclopropenone with methylphenylacetylene

Entry	Catalyst	Cat. load. (mol%)	Yield (%) (6h)	Yield (9h)
1	**4**	1	0	0
2	**5**	1	23	29
3	**6**	1	0	0
4	**7**	1	24	32
5	**5** + [$CoCp_2$]	1	0	0
6	**7** + [$CoCp_2$]	1	0	0
7	**5**	2,5	25	30
8	**5**	5	39	46
9	**5**+ [$CoCp_2$]	5	0	0

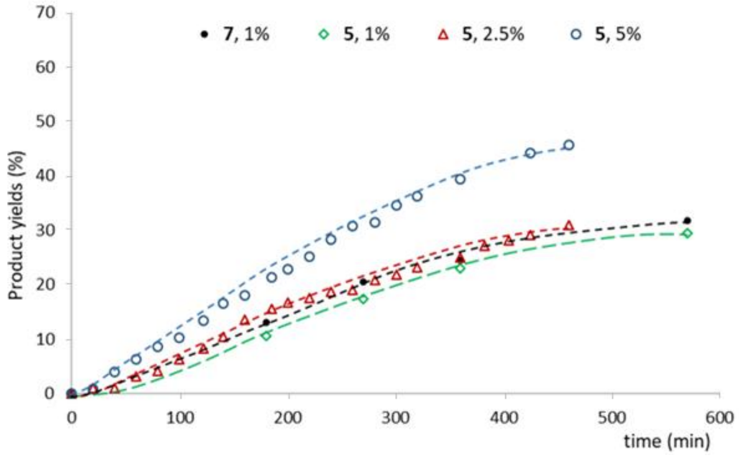

Figure S45. Time-dependent reaction profile of the cycloaddition of diphenylcyclopropenone with methylphenylacetylene with catalysts **5** (at 1, 2.5 and 5 mol %) and **7** (at 1 mol %).

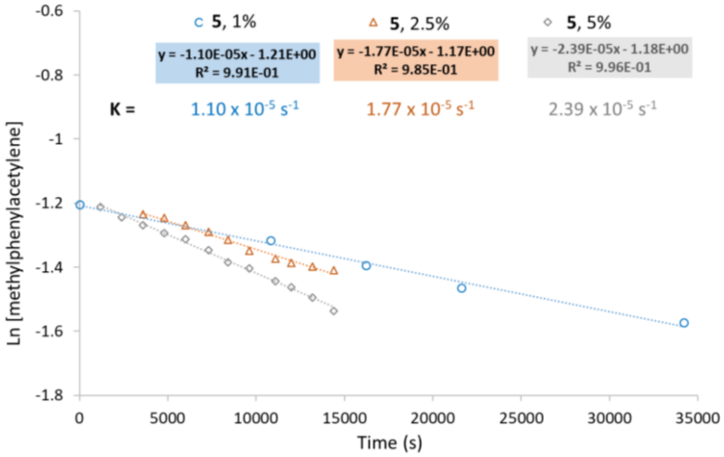

Figure S46. Non-linear least squares fit of the kinetic data assuming first order reaction.

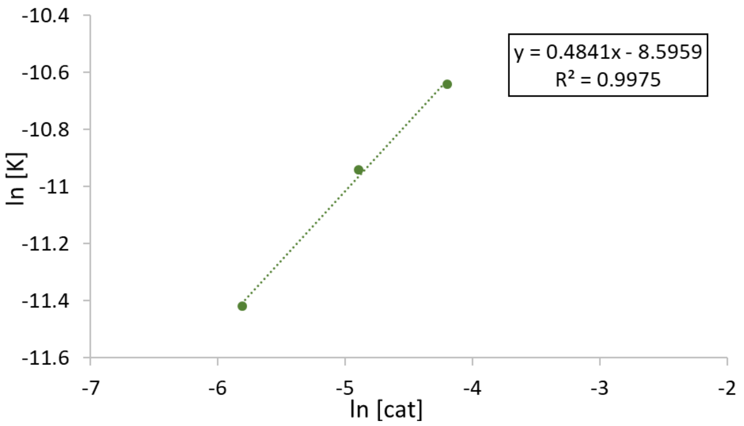

Figure S47. Dependence of the ln[constant rate] on the ln[**5**].

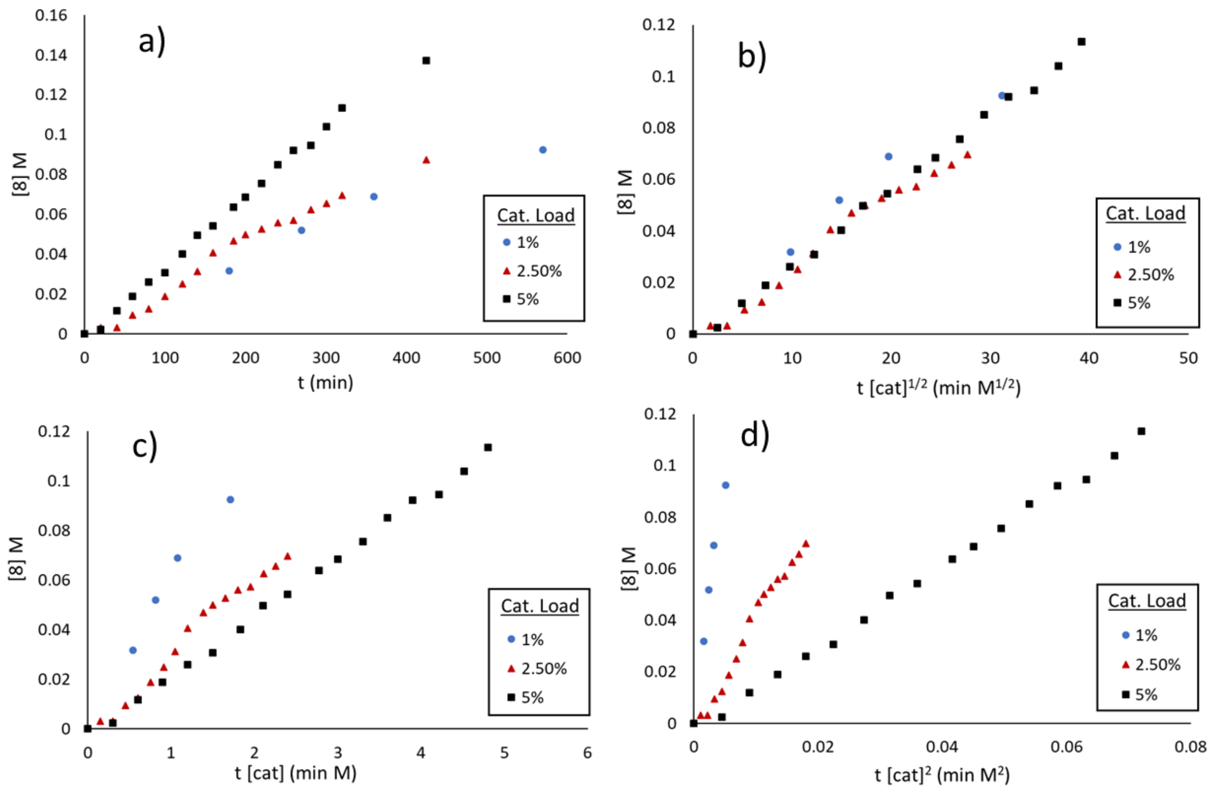

Figure S48. (a) Time-dependent reaction profile of the cycloaddition of diphenylcyclopropenone with methylphenylacetylene with catalysts **5**. (b) Reaction profile with normalized time scale assuming a catalyst order of 1/2. (c) Reaction profile with normalized time scale assuming a catalyst order of 1. (d) Reaction profile with normalized time scale assuming a catalyst order of 2.

8.2. Redox switching experiments

A stock solution containing complex **5** (2 x 0.0045 mmol, 2.5% mol), diphenylcyclopropenone (2 x 0.27 mmol, 1.5 equiv.), methylphenylacetylene (2 x 0.18 mmol, 1 equiv) and 1,3,5-trimethoxybenzene as internal reference (2 x 0.18 mmol, 1 equiv.) in 1.2 mL of toluene-*d8* was prepared in a Schlenk tube under anaerobic conditions. This solution was then distributed in two (2 x 0.6 mL) different thick-walled Schlenk tubes fitted with a Teflon cap. Cobaltocene (2 x 1.5 equiv. respect to the catalyst) was added only to one of the two Schlenk tubes. The resulting mixtures were heated at 110 ºC. The reaction without cobaltocene was monitored for 7.5 h collecting a small aliquot each 20 min (control reaction). The other one was monitored during 2 hours without appreciating the formation of the product. Afterward, 1.5 equivalent (with respect to catalyst) of a chemical oxidant, namely $[Fe(\eta_5\text{-}C_5H_4COCH_3)Cp][BF_4]$, was added to the reaction mixture under nitrogen. The activity of the catalyst was completely restored by addition of the oxidant. After collecting data for the following 2.5 hours, the reductant cobaltocene was again added to the mixture (1.5 equivalent with respect to catalyst). The addition of cobaltocene inhibits the cycloaddition reaction, but triggers the isomerization of the cyclopentadienone product (Scheme S4).

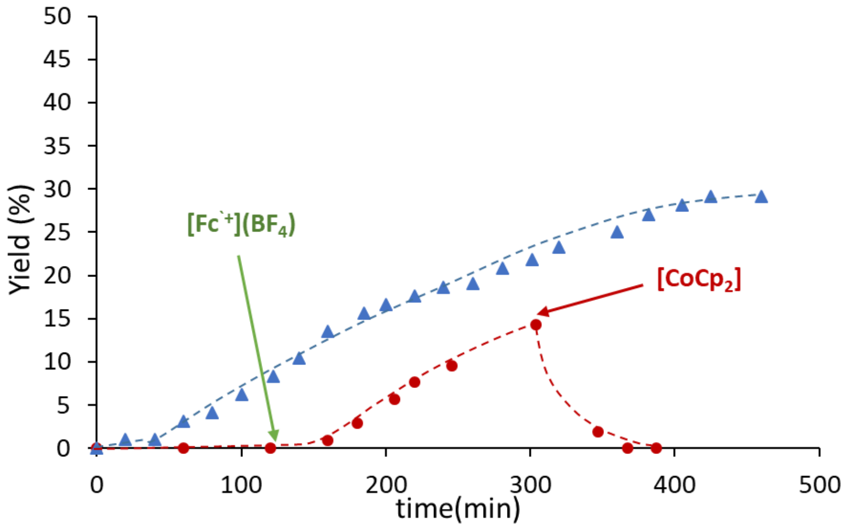

Figure 49. Switching experiment of the cycloaddition of diphenylcyclopropenone with methylphenylacetylene using 2.5 mol % of **5**, with sequential additions of $[Fe(\eta_5\text{-}C_5H_4COCH_3)Cp][BF_4]$ ($[Fc'^+](BF_4)$) and cobaltocene ($[CoCp_2]$).

In another experiment, we prepared the same solution as before but with a 5 mol % of complex **5**. This solution was then distributed in two (2 x 0.6 mL) different thick-walled Schlenk tubes fitted with a Teflon cap. The resulting mixtures were heated at 110 ºC. The

control reaction was monitored for 7.5 h collecting a small aliquot each 20 min. The other one was monitored during 3 hours. After this time, the reductant cobaltocene was added to the mixture under anaerobic conditions (1.5 equivalent with respect to catalyst). We observed the rapid disappearance of the product of cycloaddition (**8**), with the concomitant formation of the isomerization product (**9**). When all compound **8** was converted into **9**, we added [Fe(η_5-$C_5H_4COCH_3$)Cp][BF_4] (1.5 equivalent with respect to catalyst) and observed that the activity of the catalyst was drastically recovered.

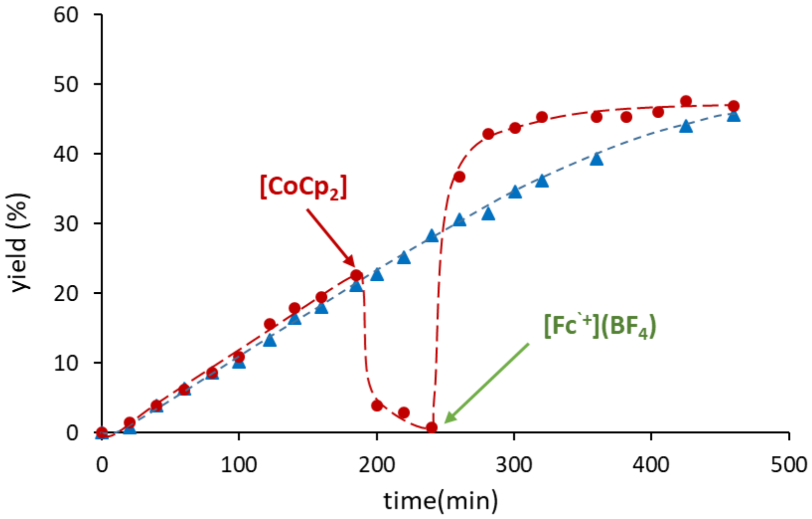

Figure 50. Switching experiment of the cycloaddition of diphenylcyclopropenone with methylphenylacetylene using 5 mol % of **5**, with sequential additions of Fe(η_5-$C_5H_4COCH_3$)Cp][BF_4] ([Fc'$^+$](BF_4)) and cobaltocene ([$CoCp_2$]).

8.3. Isomerization of cyclopentadienone

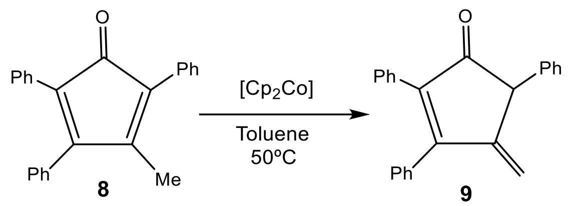

Scheme S4. Isomerization of 3-methyl-2,4,5-triphenylcyclopenta-2,4-dien-1-one (**8**) to 4-methylene-2,3,5-triphenylcyclopent-2-en-1-one (**9**) catalysed by [$CoCp_2$] at 50ºC.

In the previous experiments, we observed that the product of the cycloaddition of diphenylcyclopropenone with methylphenylacetylene (**8**) disappeared when we added cobaltocene to the reaction mixture. For this reason, the isomerization of cyclopentadienone **8** was studied in an independent experiment, by monitoring the reaction of isolated **8**. For this purpose, an NMR tube were charged with 3-methyl-2,4,5-triphenyl-2,4-cyclopentadien-1-one (**8**) (0.05 mmol, 1 eq.), 1,3,5-trimethoxybenzene

(0.05 mmol, 1 eq.) and cobaltocene (0.0005 mmol, 1 mol%). The mixture was deaerated using vacuum and filled with nitrogen three times. After this, 0.6 mL of toluene-d_8 were added to the NMR tube under nitrogen. The reactions were monitored by ^{1}H-NMR at 50ºC. Yields were determined by ^{1}H-NMR spectroscopy using 1,3,5-trimethoxybenzene as internal standard.

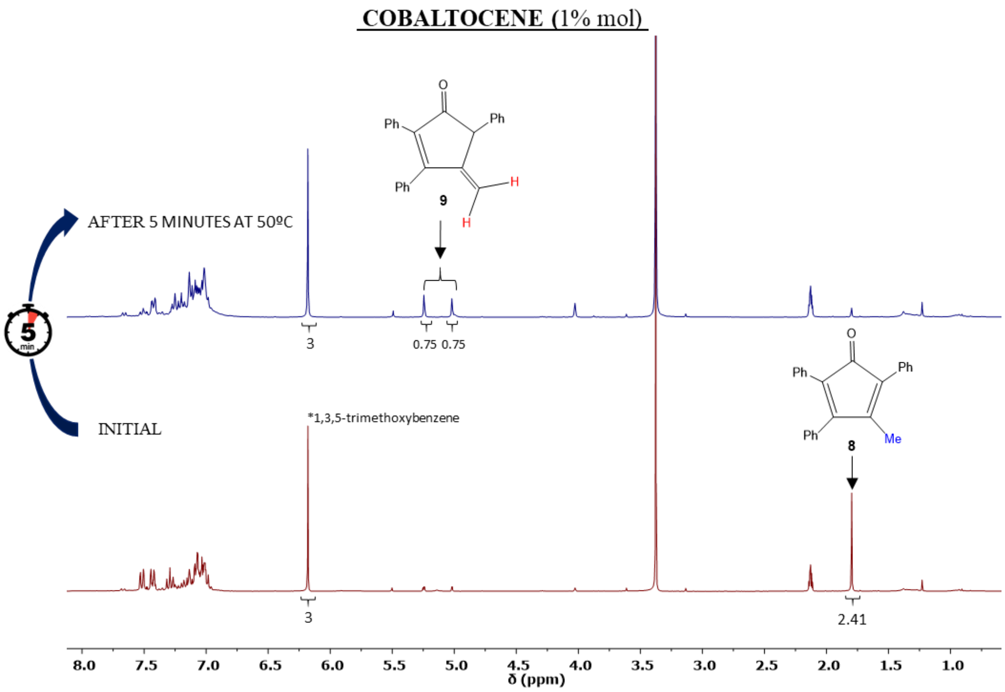

Figure 51. Selected region of the initial ^{1}H NMR spectrum (bottom) and after 5 minutes (top) of the isomerization reaction of 3-methyl-2,4,5-triphenylcyclopenta-2,4-dien-1-one (**8**) to 4-methylene-2,3,5-triphenylcyclopent-2-en-1-one (**9**) catalysed by [$CoCp_2$]. ^{1}H NMR spectra recorded at 50ºC (500 MHz) in toluene-d_8.

9. References

[1] M. W. Holman, R. C. Liu, D. M. Adams, *J. Am. Chem. Soc.* **2003**, *125*, 12649-12654.

[2] Q. F. Yan, D. H. Zhao, *Org. Lett.* **2009**, *11*, 3426-3429.

[3] G. R. Fulmer, A. J. M. Miller, N. H. Sherden, H. E. Gottlieb, A. Nudelman, B. M. Stoltz, J. E. Bercaw, K. I. Goldberg, *Organometallics* **2010**, *29*, 2176-2179.

[4] R. K. Dubey, M. Melle-Franco, A. Mateo-Alonso, *J. Am. Chem. Soc.* **2021**, *143*, 6593-6600.

[5] H. L. Wang, L. C. Chen, Y. Xiao, *J. Mater. Chem. C* **2017**, *5*, 8875-8882.

[6] a) J. Bures, *Angew. Chem., Int. Ed.* **2016**, *55*, 16084-16087; b) J. Bures, *Angew. Chem., Int. Ed.* **2016**, *55*, 2028-2031.
